# Patient-derived model capturing hypoxia and extracellular matrix remodelling of immunologically cold high-grade serous tumours

**DOI:** 10.1038/s41467-026-75262-8

**Published:** 2026-07-11

**Authors:** Simona Plesselova, Hailey Axemaker, Kristin Calar, Oduduabasi Isaiah, Jared Wollman, Somshuvra Bhattacharya, Etienne Z. Gnimpieba, Darci M. Fink, Congzhou Wang, Hiruni Sumanasiri, Kristina W. Thiel, Maria Bell, Pilar de la Puente

**Affiliations:** 1 Center for Cancer Biology Research, Sanford Research Institute, Sioux Falls, SD USA; 2 Functional Genomics and Bioinformatics Core, Sanford Research Institute, Sioux Falls, SD USA; 3 Flow Cytometry Core, Sanford Research Institute, Sioux Falls, SD USA; 4 Department of Biomedical Engineering, University of South Dakota, Vermillion, SD USA; 5 Department of Chemistry, Biochemistry & Physics, South Dakota State University, Brookings, SD USA; 6 Department of Nanoscience and Biomedical Engineering, South Dakota School of Mines and Technology, Rapid City, SD USA; 7 Department of Obstetrics and Gynecology, University of Iowa, Iowa City, IA USA; 8 Holden Comprehensive Cancer Center, University of Iowa, Iowa City, IA USA; 9 Sanford Health, Sanford Gynecologic Oncology Clinic, Sioux Falls, SD USA; 10 Department of Obstetrics and Gynecology, University of South Dakota Sanford School of Medicine, Sioux Falls, SD USA; 11 Department of Surgery, University of South Dakota Sanford School of Medicine, Sioux Falls, SD USA

**Keywords:** Cancer microenvironment, Immune evasion, Tissues, Transforming growth factor beta

## Abstract

High-grade serous carcinoma tumours present poor survival rates, often associated with immunologically excluded environments driven by hypoxia and extensive extracellular matrix remodelling that disrupt tumour-stromal-immune interactions. Current experimental models fail to fully capture these microenvironmental features, limiting understanding of tumour-immune dynamics and drug development. Here, we present bioengineered patient-derived tumour-immune models to mimic physiologically relevant oxygen levels and extracellular matrix remodelling. Cancer cells are co-cultured with cancer-associated fibroblasts within human plasma-3D matrices or grown on decellularized human ovaries. Immune cells are either included within the 3D constructs to study multi-cellular interactions or challenged to infiltrate the matrices. We demonstrate that intratumoural hypoxia acts as a friend and a foe enhancing the activation and cytotoxicity of CD8 + T cells while inducing stromal/matrix dysregulation associated with impaired immune infiltration. Targeting TGF-β signalling attenuates the hypoxia-driven stromal-mediated immune exclusion. These relevant models may aid the development of targeted therapies to transform immunologically cold tumours into immunogenic to benefit female patients.

## Abstract (web summary)

High-grade serous carcinoma of the ovaries is a hard-to-treat tumour with poor prognosis, characterized by an immunologically hostile microenvironment. Here the authors present an in vitro model of these carcinomas, which accurately capture the main features of the tumours, including hypoxia, extracellular matrix remodelling and immune exclusion.

## Introduction

High-grade serous carcinoma (HGSC) is the most common, aggressive, and lethal gynaecological malignancy, accounting for 70–80% of ovarian cancer deaths^1^. The 5-year survival rate for advanced-stage HGSC patients is <30%^2^. The standard-of-care treatment for HGSC typically involves primary cytoreductive surgery followed by platinum/taxane-based chemotherapy^3^. While the initial response to the chemotherapy regimen is usually good, the majority of HGSC patients progress during the initial treatment or recur in <6 months after completing treatment^3^. Interestingly, ovarian cancer was among the first cancers where the positive correlation between tumour-infiltrating lymphocytes (TIL) and improved clinical outcome was identified in patients with advanced stage^4^. Similarly, the presence of intraepithelial CD3^+^ and CD8^+^ TILs is associated with a prognostically favourable survival benefit in HGSC patients^5,6^. Long-term HGSC survivors present enhanced immune activity with activated memory CD4^+^ T cells, activated natural killer (NK) cells, and higher densities of CD8^+^ T cells^7,8^.

Unfortunately, most HGSC patients are refractory to immunotherapies^9^ and present immunologically cold tumours characterised by immune exclusion from a complex heterogenous immunosuppressive tumour microenvironment (TME) defined by an intricate interplay between the cancer cells, cancer-associated fibroblasts (CAF), and the extracellular matrix (ECM)^10,11^. The ECM is the acellular component of the TME that undergoes constant remodelling and provides biochemical and structural support to the cells^12^. CAFs are key players in the dysregulated secretion of ECM proteins through transforming growth factor-β (TGF-β) signalling, which causes a stiff physical barrier hindering immune cell and drug penetration into the TME^13–15^. Furthermore, the composition and biomechanical cues of the ECM can alter the cellular behaviour and drug response of the ovarian cancer cells^16–18^ as well as of the immune cells infiltrated into the TME^19^. Hypoxia, or low oxygen levels, is a key hallmark of tumours that governs CAF activation and ECM remodelling, interferes with drug resistance, and promotes tumour growth, immune evasion, and metastatic potential^20^. Conversely, hypoxia correlates with enhanced CD8^+^ T cell activation, inducing a higher cytotoxic effect of TILs on cancer cells^21^. Although there are neither tumour nor healthy ovarian tissue empirical measurements of the partial pressure of oxygen (pO_2_), it has been reported that the mean oxygen saturation of human ovarian epithelial cancers is 9.1% lower than normal and benign ovaries^22^. Ovarian tumours ranked among the most hypoxic tumour types according to transcriptomic data^23^, and oxygen levels lower than 0.2 kPa were measured in mouse ovarian cancer xenografts^24^. In general, intratumoural hypoxia in all tumour types is characterised by median oxygen levels lower than 2 kPa^25^. Importantly, tumours initiate in tissue characterised by physoxic oxygen levels, low ECM remodelling and higher levels of immune infiltration^1,26^. Physoxic oxygen levels in the healthy human ovary revealed arterial and venous pressures of 14.63 kPa and 11.73 kPa respectively^27^, with follicular fluid dissolved oxygen levels in healthy human follicles ranging from 7.2 kPa to 16.7 kPa^27–29^. Unfortunately, the majority of preclinical cancer models inaccurately compare intratumoural hypoxia to normoxic ambient air 21% O_2;_ however, these levels are hyperoxic with respect to the tissue of origin. Such comparisons fail to account for the physoxic oxygen levels present in the tissue microenvironment in which HGSC initiation and early progression occur, as well as the progressive hypoxic conditions characteristic of advanced-stage disease. As a result, critical oxygen-dependent tumour–stroma–immune interactions are often inadequately modelled. To more accurately study these processes, hypoxic conditions representative of late-stage ovarian cancer should be evaluated in comparison to physoxic oxygen levels that reflect the early-stage HGSC microenvironment, rather than to atmospheric oxygen.

In this study, we bioengineered 3D HGSC models that recapitulate disease-relevant oxygen tensions and ECM remodelling features associated with early and advanced stages of tumour progression, enabling the investigation of cell-cell, cell-ECM interactions, and immune infiltration patterns in a controlled yet physiologically informed context. Peripheral blood mononuclear cells (PBMC) were incorporated into the system using two distinct and independent experimental configurations. In the first configuration, PBMCs were included within the 3D construct as a triculture, together with HGSC tumour cells and CAFs, allowing direct assessment of tumour–immune interactions within the engineered microenvironment. In the second configuration, PBMCs were challenged to infiltrate either the engineered 3D HGSC models or decellularized human ovarian ECM, enabling analysis of immune infiltration dynamics and immune–ECM interactions. We demonstrated that intratumoural hypoxia drives ECM remodelling through activated CAFs via TGF-β signalling and alters tumour-immune interactions through the collagen signalling pathway with enrichment of immunosuppressive CD4^+^ T cells and cytotoxic granzyme B^+^ CD8^+^ T cells. Our bioengineered tumour models recapitulated hypoxia-induced CAF-driven ECM remodelling and reduced CD8⁺ T cell infiltration, which are hallmarks of immunologically cold tumours. Importantly, TGF-β signalling inhibition with galunisertib attenuated immune exclusion and promoted T cell infiltration in this model. Moreover, hypoxia induced higher expression of pro- and anti-inflammatory cytokines involved in tumour progression, which was reversed by targeting TGF-β signalling. Validation studies confirmed low CD8^+^ T cell presence in areas with high collagen expression in HGSC patients, as well as significantly impaired immune infiltration associated with hypoxia-induced CAF-driven ECM remodelling in decellularized human ovaries, which was reduced by preincubation with galunisertib. Our work provides a paradigm-shifting understanding of intratumoural hypoxia in advanced HGSC by contrasting hypoxic TMEs with physoxic oxygen levels characteristic of early-stage disease. This approach provides mechanistic insight into hypoxia-induced CAF-driven ECM remodelling and its impact on immune evasion and cellular interactions, and facilitates screening of targeting vulnerabilities to reprogramme immunologically cold HGSC tumours into hot ones in this model system.

This study establishes bioengineered tumour–immune models that recapitulate hypoxia-driven ECM remodelling and enable dissection of tumour–stromal–immune interactions under physiologically relevant conditions. The results show that hypoxia exerts a dual role by enhancing CD8⁺ T cell activation while simultaneously promoting stromal-mediated immune exclusion through TGF-β signalling, which can be mitigated by pathway inhibition. These findings identify a mechanistic link between hypoxia, matrix remodelling, and immune infiltration dynamics in HGSC. These insights highlight actionable opportunities to overcome immune exclusion. The approach provides a versatile platform to guide the development of strategies aimed at converting immunologically cold tumours into clinically responsive disease.

## Results

### Engineered tumour models align transcriptomically with tumours and functionally recapitulate hypoxia- and ECM-associated phenotypes

**Fig. 1:**
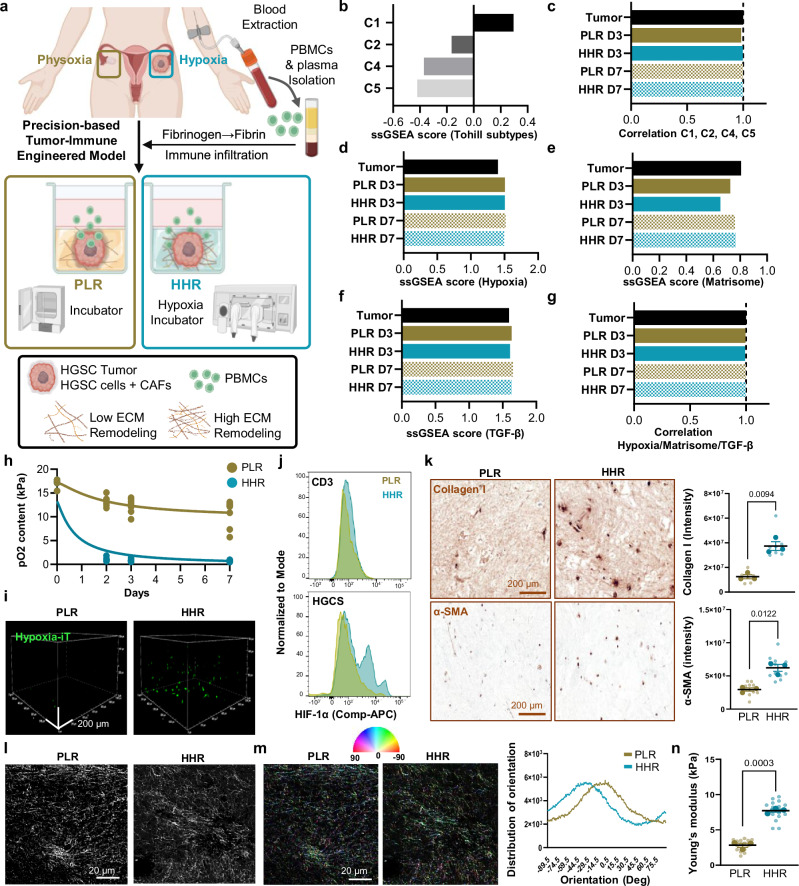
Transcriptomic concordance and functional divergence across hypoxia and ECM remodelling in patient-derived 3D models. **a** Schematic of patient-derived 3D models PLR (physoxia-low remodelling of ECM) and HHR (hypoxia-high remodelling of ECM) mimicking O_2_ levels and ECM biomechanical cues in the early and late stage HGSC, respectively. Created in BioRender. de la Puente, P. (2026) https://BioRender.com/hw5q2l8. **b** Single-sample Gene Set Enrichment analysis (ssGSEA) scores for molecular subtypes of HGSC defined by Tothill et. al (2008): C1 (mesenchymal), C2 (immunoreactive), C4 (differentiated) and C5 (mesenchymal-like) in HGSC tumour detected by bulk RNA-seq. **c** Similarity of gene set expression for Tothill subtypes in fresh tumour and dissociated tumour grown in PLR or HHR for 3 (PLR&HHR D3) or 7 days (PLR&HHR D7) expressed by Pearson correlation. **d** ssGSEA scores for hypoxia, **e** matrisome, **f** TGF-β signalling gene sets enrichment and **g** similarity of gene enrichment for the three pathways between the tumour and tumour grown in the 3D models. **h** Oxygen levels measured inside PLR and HHR models (0, 2, 3, and 7 days) by an optical sensor. *n* = 7 cell lines, four technical replicates each. **i** Confocal microscopy images in 3D view of hypoxic cells detected with Image-iT™ Green Hypoxia dye. *z* = 500 μm, scale = 200 μm. Same results were identified for three independent models. **j** Histograms of HIF-1α expression in co-cultured primary HGSC tissue with autologous PBMCs. *n* = 2 patient samples **k** Representative IHC images for collagen I and α-SMA in co-culture of KURAMOCHI with CAFs and their quantification, scale = 200 μm. **l** SHG images of collagen fibres in co-culture and **m** quantification of distribution of orientation. Scale = 20 μm. Same results were identified for three independent models. **n** Stiffness quantification by AFM represented by Young’s Modulus (kPa) in co-cultures. **k**, **n** Mean ± SD, Welch’s *t* test two-tailed. Data are represented as superplots with biological replicates shown as larger, darker symbols (*n* = 3 PLR/HHR cultures from different days) and technical replicates as smaller, lighter symbols (*n* = 3 for collagen, *n* = 4 for α-SMA and *n* = 5 for stiffness). Source data are provided as a Source Data file.

To investigate the role of physiologically relevant oxygen levels and ECM remodelling states in regulating immune infiltration and immune-cancer-stroma interactions across HGSC progression, we bioengineered a pair of patient-derived 3D models, termed PLR (physoxia–low ECM remodelling) and HHR (hypoxia–high ECM remodelling). These models are based on the crosslinking of fibrinogen into fibrin naturally present in peripheral blood plasma, generating biologically relevant ECM that recapitulates key features of HGSC tissue contexts^30,31^. HHR is a 3D scaffold designed to model the aberrant, highly remodelled ECM characteristic of advanced-stage HGSC TMEs when cultured under hypoxic conditions. In contrast, PLR reflects features of early-stage HGSC microenvironments with low ECM remodelling and physoxic oxygen levels when incubated in a standard incubator (Fig. 1a). Both PLR and HHR 3D models were generated under two experimental settings. In the patient-derived setting, dissociated primary HGSC tumour cells were cultured in the presence of autologous plasma and PBMCs, preserving patient-specific tumour-immune interactions. In parallel, immortalised HGSC cancer cell lines and CAFs were incorporated into 3D scaffolds generated with healthy donor–derived plasma and PBMCs, reflecting the lack of autologous material for established cell lines and providing a controlled immune background. Importantly, all models, regardless of plasma origin, were cultured under both physoxic and hypoxic conditions, allowing direct comparison of oxygen-dependent effects across healthy and cancer plasma contexts.

First, transcriptomic profiling of a dissociated HGSC tumour grown in PLR or HHR for 3 or 7 days was performed using bulk RNA sequencing, using fresh native HGSC tumour (not grown in 3D scaffolds) as a control for benchmarking analysis. Simple sample gene enrichment analysis (ssGSEA) revealed that both the native HGSC tumour (Fig. 1b) and the tumour grown in the 3D models (Supplementary Fig. 1a), presented the highest net score for molecular HGSC subtype C1 (mesenchymal) compared to C2 (immunoreactive), C4 (differentiated) or C5 (mesenchymal-like) defined by Tothill^32^. All 3D patient models exhibited strong positive correlation (Pearson *r* ≥ 0.98) with the native tumour, indicating that the overall pattern of subtype-specific transcriptional activity observed in the native tumour is highly conserved across the 3D models (Fig. 1c and Supplementary Fig. 1b). In addition, gene signatures for key microenvironmental features in the TME related to hypoxia, matrisome, and TGF-β signalling were evaluated across all samples showing consistently high enrichment in both the native tumour and the 3D models. Hypoxia (Fig. 1d), matrisome (Fig. 1e), and TGF-β signalling (Fig. 1f) ssGSEA scores present in the patient tumour were broadly preserved in the PLR and HHR models at both time points. All 3D tumour models showed strong positive correlation with native tumour for these features (Pearson *r* ≥ 0.99), indicating that hypoxia/matrisome/TGF-β signalling transcriptomic profiles were conserved (Fig. 1g). Together, these findings demonstrate that the molecular fingerprints defining the HGSC subtype and key microenvironmental and signalling pathways are consistently maintained across the 3D tumour models compared to native tissue.

Second, to functionally characterise the PLR and HHR models, we performed studies to assess the oxygen levels by measuring the partial pressure of oxygen (pO_2_) inside the 3D scaffold using an optical sensor. When the 3D scaffold with ovarian cancer cell lines co-cultured with CAFs and PBMCs was incubated in a 21% O_2_ incubator, the average oxygen levels inside the PLR were approximately 12-13 kPa on days 2–3 and 10 kPa on day 7, recreating the physoxic O_2_ levels at the site of tumour initiation. When incubated in a 1.5% O_2_ hypoxic chamber, the oxygen level in HHR was significantly lower, reaching 0.8–1 kPa on days 2–3 and decreasing to 0.7 kPa on day 7, recreating the hypoxic advanced-stage HGSC-TME (Fig. 1h). Moreover, the hypoxic status of KURAMOCHI multi-cultured with CAFs and PBMCs inside PLR and HHR was confirmed using a hypoxia-sensitive green dye. Green hypoxic cells were observed in multi-cultures (Fig. 1i) as well as monocultures (Supplementary Fig. 1c) in HHR compared to PLR. Similar results were observed when dissociated HGSC tumour was grown in monoculture or co-culture with CAFs using hypoxia-sensitive green dye (Supplementary Fig. 1d) and physiologically relevant O_2_ levels were confirmed by the optical sensor reaching ~14 kPa in PLR and 1.2 kPa in HHR (Supplementary Fig. 1e). Furthermore, the expression of HIF-1α, a classical transcription factor overexpressed in hypoxia, was slightly higher in CD3^+^ T cells and robustly elevated in HGSC cells in HHR compared to PLR when primary HGSC tissue was co-cultured with autologous PBMCs and assessed by flow cytometry (Fig. 1j and Supplementary Fig. 1f). Similar results were observed in a multi-culture of KURAMOCHI with CAFs and PBMCs by IHC staining (Supplementary Fig. 1g). Therefore, this functional analysis revealed that PLR and HHR biomimetic models can recapitulate the physiologically relevant oxygen levels in the site of tumour origin and the intratumoural hypoxia in advanced stage HGSC-TME, respectively.

Third, to assess the impact of oxygen tension on ECM remodelling, immunohistochemical analysis revealed significantly higher deposition of collagen I and increased expression of α‑SMA, a marker of CAF activation, in KURAMOCHI cells co‑cultured with CAFs under HHR conditions compared to PLR (Fig. 1k). CAF monocultures also exhibited increased collagen I deposition under HHR, although at lower levels than observed in co‑cultures, while no collagen I or α‑SMA expression was detected in KURAMOCHI monocultures. In contrast, when PBMCs were included in multicultures, collagen I deposition was reduced despite sustained CAF activation (Supplementary Fig. 2a, b). Consistent with increased matrix remodelling activity, MMP‑9 expression was significantly elevated in hypoxic multicultures containing PBMCs, but not in monocultures or HGSC–CAF co‑cultures (Supplementary Fig. 2c, d). Second harmonic generation microscopy confirmed that collagen fibre formation occurred only in the presence of CAFs, independent of oxygen condition. Collagen fibres in co-cultures displayed differences in orientation, with a preferred fibre direction in HHR defined by the shift of the peak towards ~−35^o^ compared to PLR (0^o^) and slightly more aligned organization in HHR defined by a broader distribution of orientation in PLR (Fig. 1l, m). No differences were observed in the distribution of collagen fibres in CAF monocultures between PLR and HHR (Supplementary Fig. 2e, f). Atomic force microscopy measurements performed on HGSC–CAF co‑cultures demonstrated significantly higher matrix stiffness under HHR compared to PLR (Fig. 1n), consistent with increased collagen deposition in the absence of immune‑mediated matrix remodelling. Together, these results indicate that hypoxia promotes CAF activation and collagen‑driven ECM stiffening, while the presence of immune cells introduces additional matrix remodelling dynamics that modulate net collagen accumulation without abrogating hypoxia‑induced stiffening.

By modulating oxygen availability and ECM composition, we were able to generate culture conditions that align transcriptomically to HGSC tumours and functionally recapitulate oxygen and ECM remodelling associated with early and advanced stages of ovarian cancer initiation and progression. This system therefore enables us to systematically study how oxygen levels and ECM remodelling influence tumour cell behaviour and tumour-immune interactions within a physio- and pathologically relevant 3D context.

### Intratumoural hypoxia drives ECM remodelling and immune dysfunction in physiologically relevant tumour-immune HGSC models

**Fig. 2:**
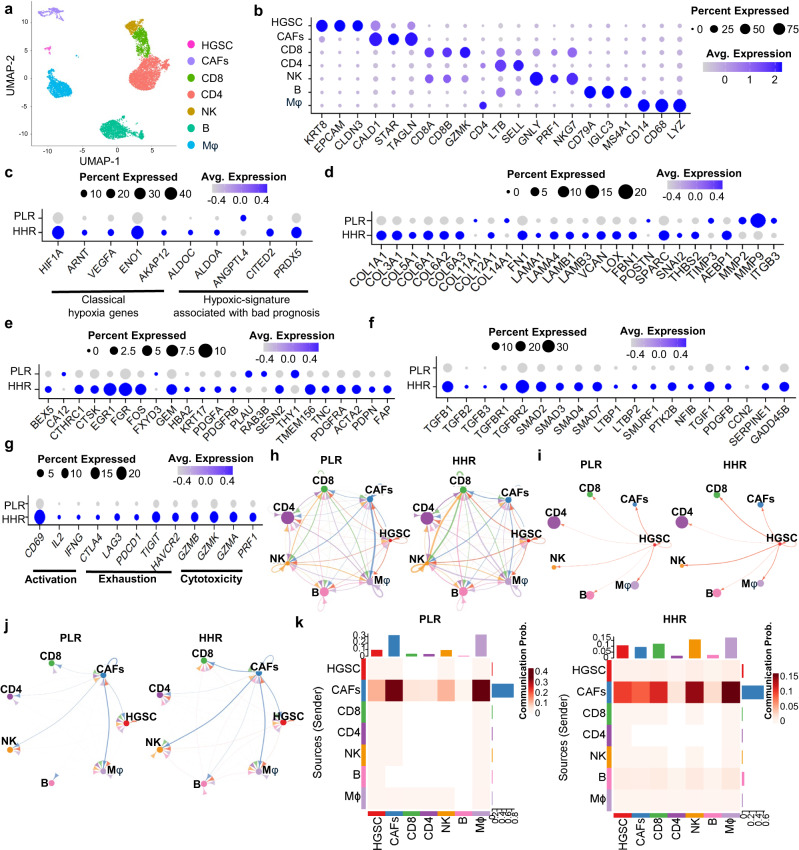
Single-cell RNA sequencing identifies intratumoural hypoxia as a driver of ECM remodelling, immune functionality, and tumour-immune interactions. **a** UMAP plot showing clusters of annotated cell populations. **b** Bubble plot representing the expression of specific marker genes in each detected cell cluster. **c** Bubble plots showing the gene expression of classical hypoxia genes and hypoxic signature correlated with bad HGSC prognosis, **d** matrisome, **e** CAF-associated genes, **f** TGF-β signalling genes, and **g** T-cell functionality genes (activation, exhaustion, cytotoxicity) in co-culture of HGSC tumours with PBMCs inside the PLR vs HHR. **h** Circle plots of interactions (weights/strength) among all cell populations, and **i** HGSC cells with the rest. **j** Circle plots of interactions (weights/strength) among cells in the collagen pathway. **k** Heatmap representing the probability of communication among cells in the collagen pathway.

To explore the role of physoxia and hypoxia on ECM and the immune landscape at the single-cell transcriptomic level, single-cell RNA sequencing (scRNA-seq) was performed on co-cultures of dissociated HGSC tumour with matching PBMCs in PLR and HHR using native PBMCs as a control. We identified seven single-cell clusters corresponding to different cell types in the TME (Fig. 2a, b and Supplementary Fig. 3a, b), including HGSC epithelial cells (HGSC), CAFs, CD8^+^ and CD4^+^ lymphocytes (CD8 and CD4), natural killer cells (NK), B cells (B), and macrophages (Mφ). Immune cell type populations were identified in all experimental conditions (PBMCs, PLR, and HHR), while HGSC and CAF cells were only present in PLR and HHR, as expected (Supplementary Fig. 3c). Marker genes were conserved in both engineered models when compared to native PBMCs (Supplementary Fig. 3d), suggesting that both models largely capture the transcriptomic profile of immune populations present in PBMCs. Therefore, only PLR and HHR models were further compared to determine the influence of physoxic and hypoxic oxygen levels in ECM dynamics and immune cell behaviour. Classical hypoxia genes and hypoxic gene signatures correlated with bad prognosis in HGSC patients^33^ corroborated hypoxic features in HHR when compared to PLR (Fig. 2c and Supplementary Fig. 3e). Similarly, intratumoural hypoxia in HHR influenced ECM remodelling at the transcriptomic level including enriched matrisome (ECM-related) genes (Fig. 2d), CAF-associated genes (Fig. 2e), and TGF-β pathway genes (Fig. 2f). While matrisome and CAF-associated genes were mostly exclusively expressed by CAFs, TGF-β signalling genes were expressed by all cell types (Supplementary Fig. 4a). To explore the effect of physiological oxygen levels on immune functionality, we evaluated activation, exhaustion and cytotoxicity genes and found them highly expressed in HHR when compared to PLR, specifically expressed by cytotoxic effector cells including CD8^+^T and NK cells (Fig. 2g and Supplementary Fig. 4b). Moreover, when the transcriptional profile of HHR was compared to PLR, we found 11,695, 11,645, 11,212, 11,993, 10,368, 9,665, 11,395, and 8927 differential expressed genes (DEG) for all populations, HGSC, CAFs, CD8, CD4, NK, B and Mφ, respectively (Supplementary Fig. 4c and Supplementary Data 1). Importantly, ECM remodelling (matrisome and TGF-signalling), as well as immune activation, exhaustion and cytotoxicity genes, were significantly upregulated in HHR compared to PLR (Supplementary Fig. 4c and Supplementary Data 1). Furthermore, we investigated the probability and the strength of cancer-stroma-immune signalling interactions using CellChat. Stronger intercellular interactions were detected in HHR compared to PLR (Fig. 2h). A closer look at the specific interactions in each cell type with the rest of the cell populations confirmed that there were stronger interactions of HGSC cells with CAFs, CD8^+^, and NK cells (Fig. 2i), and of CAFs with CD8^+^ and NK cells (Supplementary Fig. 5a) in HHR compared to PLR. Similar trends were observed among other immune cells with NK and CD8 + T cells in the HHR compared to PLR, and strong interactions of all cells with macrophages were observed independently of oxygen conditions (Supplementary Fig. 5a). To further explore the role of hypoxia and ECM remodelling on the cellular interactions in the TME, we studied the interactome of all cell types in the collagen pathway. The analysis of the strength and probability of the interactions of all cell populations in the collagen pathway indicated that CAFs are the main contributors with stronger interactions with CD8^+^ T cells, NK cells, B cells, and HGSC cells in HHR when compared to PLR, and also, they have a strong and high probability of interactions with Mφ independently of oxygen levels (Fig. 2j, k). Under close examination, we observed that CAFs acted as the main senders, receivers, mediators, and influencers of the collagen pathway signals in both PLR and HHR, confirming their key role in ECM remodelling and interactions with the rest of the cells through collagen signalling. Also, HGSC, CD8, and NK cells gained more roles as receivers of the signals in the collagen pathway in HHR compared to PLR (Supplementary Fig. 5b, c). Moreover, we identified important ligand-receptor pairs and their contribution on collagen pathway interactions such as the ligands (COL1A2, COL1A1, COL6A1) and receptors (CD44, SDC4, ITGA1 + ITGB1, ITGA2 + ITGB1, etc.) highlighting COL1A2-(ITGA1 + ITGB1) and (ITGA2 + ITGAB1) as the main contributors under intratumoural hypoxia (Supplementary Fig. 5d). Specifically, the probability of ligand-receptor interactions with the rest of the cells was higher in CAFs (Supplementary Fig. 5e) and in HGSC cells (Supplementary Fig. 5f) in the HHR when compared to PLR, even though there was a lower number of possible interactions correlated with the collagen signalling pathway in the HGSC cells than in CAFs, indicating that CAFs are key players in ECM remodelling. Altogether, the data suggest that, at the single-cell transcriptomic level, intratumoural hypoxia, when compared to physoxia is a key driver of ECM remodelling through CAFs, sustains the functionality of cytotoxic effector cells via activation, exhaustion, and cytotoxicity, and influences cancer-stroma-immune interactions through the collagen pathway, enabled by our physiologically relevant HGSC models.

### The HHR model recapitulates T cell effector functions of HGSC tumours

**Fig. 3:**
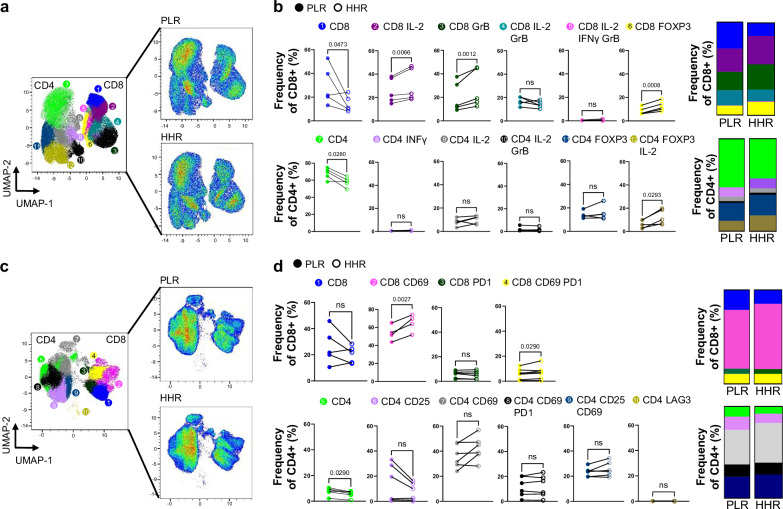
HHR model recapitulates T-cell effector functions of HGSC tumours. **a** UMAP plot showing clusters of detected populations and density plots comparing PLR vs HHR in co-culture of HGSC tumours with autologous PBMCs inside the 3D models. **b** Donor-paired dot plots representing the expression of intracellular markers of activation (IL-2, IFNγ), exhaustion (FOXP3) and cytotoxicity (granzyme B, GrB) represented as frequency (percentage of parent) in CD4+ and CD8 + T cells. **c** UMAP, density plots, and **d** quantification of activation (CD25, CD69) and exhaustion (PD1, LAG3) surface markers represented as frequency (percentage of parent) of CD4+ or CD8 + T cells. *n* =  5 PBMCs from different donors. **b**, **d** Paired *t* test two-tailed. ns not significant. Source data are provided as a Source Data file.

To evaluate the impact of oxygen levels and ECM remodelling features on immune-cancer interplay and immune effector functions within the TME, primary tumour cells from dissociated HGSC tumours were co-cultured with autologous PBMCs within PLR or HHR matrices made with matching plasma. Unbiased dimensionality reduction analysis using a UMAP algorithm for an intracellular panel on T cells revealed that intratumoural hypoxia and high remodelled ECM induced significantly higher activation (CD8^+^IL-2^+^) and cytotoxicity (CD8^+^GrB^+^) of CD8^+^ T cells and significantly higher frequency of T regulatory (T-reg) cells (CD4^+^FOXP3^+^IL2^+^ and CD8^+^FOXP3^+^) in HHR compared to PLR at the expense of reduced CD8^+^ and CD4^+^ T cells (Fig. 3a, b and Supplementary Fig. 6a). Moreover, using a panel for activation and exhaustion surface markers on T cells, we identified that while the frequency of activated (CD8^+^CD69^+^) and exhausted (CD8^+^CD69^+^PD-1^+^) CD8 + T cells was significantly greater in HHR compared to PLR, CD4^+^ T cells only had a modest, not significant, trend to activation (CD4^+^CD69^+^) (Fig. 3c, d and Supplementary Fig. 6b). To functionally corroborate cytotoxic potential of the immune cells, apoptosis assays were performed in KURAMOCHI cells in monoculture or co-culture with PBMCs inside the 3D scaffold and the percentage of live, early apoptotic and late apoptotic/dead cells were assessed by flow cytometry. While the KURAMOCHI apoptotic stage remained unaffected by oxygen conditions, the presence of PBMCs in co-culture with cancer in HHR had a significant increase in both early and late apoptosis/death when compared to PLR (Supplementary Fig. 6c). Furthermore, when PBMCs were co-cultured with KURAMOCHI at increasing PMBC:HGSC ratios in 2D, we confirmed that the frequency of CD8^+^GrB^+^ increased in a concentration-dependent manner under hypoxia compared to normoxia, which translated to a higher cytotoxic effect in hypoxia by reducing significantly the percentage of live HGSC cells (Supplementary Fig. 6d). In conjunction, these results indicate that intratumoural hypoxia and ECM remodelling induce higher effector functions in CD8+ cytotoxic T cells, by enhancing expression of activation and cytotoxicity markers, and also the activation of T-reg-like immunosuppressive cells in the HHR model when compared to physiological PLR.

### The HHR model preserves hypoxia-induced CAF-driven ECM remodelling associated with impaired immune infiltration

**Fig. 4:**
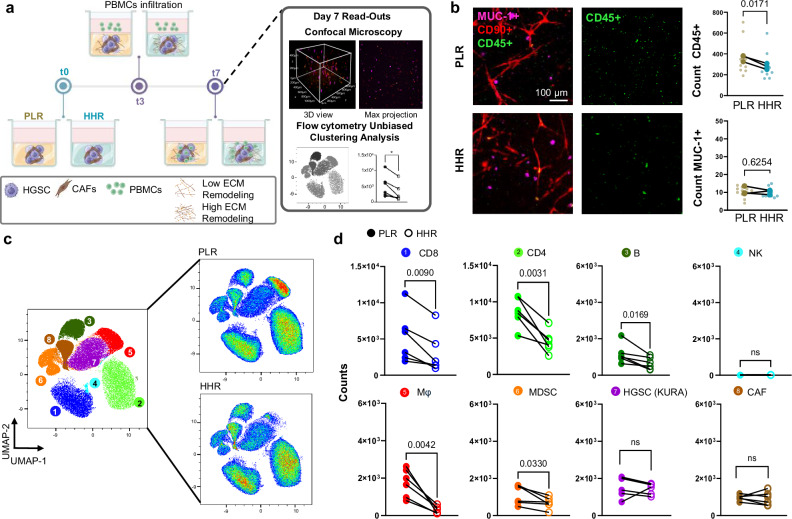
Hypoxia-induced CAF-driven ECM remodelling preserved in HHR model is associated with impaired immune infiltration. **a** Schematic representation of methods used in immune infiltration studies. Created in BioRender. de la Puente, P. (2026) https://BioRender.com/wk2de6b. **b** Representative images of confocal microscopy z-stack view as maximum projection for CD45+ PBMCs (green) infiltrated into the scaffold with KURAMOCHI (MUC-1 + , magenta) and CAFs (CD90 + , red) and donor-paired dot plots showing the number of infiltrated CD45+ and resident MUC-1 + HGSC cells. Scale = 100 μm. Data are represented as donor-paired superplots with biological replicates shown as larger, darker symbols (*n* = 3 PLR/HHR cultures from different PBMC donors) and technical replicates as smaller, lighter symbols (*n* = 3). **c** UMAP plot, density plots, and **d** donor-paired dot plots representing the number (counts) of infiltrated PBMCs (CD3 + , CD4 + , and CD8 + T cells, Mφ (CD14 + ), NK (CD56 + ), B (CD19 + ) and MDSC (CD11b + )), and HGSC and CAFs cultured inside the 3D scaffolds using dimensionality reduction analysis by flow cytometry. *n* = 6 PBMCs from different donors. **b**, **d** Paired *t* test two-tailed, ns not significant. Source data are provided as a Source Data file.

Given that the majority of HGSC patients present immune-deserted or “cold” tumours characterised by immune exclusion^9^, we assessed the effects of hypoxia-induced CAF-driven ECM remodelling on immune infiltration. KURAMOCHI cells were monocultured or co-cultured with CAFs for three days to ensure physiologically relevant oxygen levels and to allow ECM remodelling inside the PLR or HHR. On day 3, PBMCs were added on top and challenged to infiltrate the 3D cultures and then evaluated by confocal microscopy and flow cytometry on day 7 (Fig. 4a). Co-cultures vs monocultures were established to delineate the individual effects of hypoxia vs hypoxia-induced CAFs. Confocal microscopy revealed a significantly decreased infiltration of CD45^+^ lymphocytes (green) and no significant changes to KURAMOCHI counts in HHR compared to PLR when HGSC was co-cultured with CAFs (Fig. 4b). Similar results were observed in KURAMOCHI monocultures (Supplementary Fig. 7a). An immunophenotyping study of infiltrated cells identified eight populations including six immune cell populations (CD8^+^ T cytotoxic cells, CD4^+^ T helper cells, CD14^+^ Mφ, CD19^+^ B cells, CD56^+^ NK cells and CD11b^+^ myeloid-derived suppressor cells (MDSC)), HGSC (B7H3^+^), and CAFs (fibroblast activation protein, FAP^+^) by dimensionality reduction analysis using a UMAP algorithm (Fig. 4c and Supplementary Fig. 7b). Significantly impaired immune infiltration of CD8^+^ T cells, CD4^+^ T cells, B cells, Mφ, and MDSCs was observed in HHR compared to PLR while no significant changes were detected in NK cells, HGSC nor CAFs when KURAMOCHI was co-cultured with CAFs (Fig. 4d). When KURAMOCHI was monocultured, there was significantly impaired infiltration of Mφ in HHR compared to PLR, however no significant differences among the rest of the immune cell populations (Supplementary Fig. 7c). To distinguish between impaired immune infiltration due to reduced chemotactic signalling versus increased physical barriers, we quantified immune-attractant chemokines secreted by tumour and stromal cells under PLR and HHR conditions prior to PBMC addition. CXCL10, CCL2, and MIF were significantly increased in tumour-CAF co-cultures compared to either monoculture, indicating that tumour-stromal interactions enhance immune-attractant potential. Notably, CXCL10, a key chemoattractant for T cells and NK cells^34^, was significantly elevated in HHR relative to PLR co-cultures, despite reduced immune infiltration under hypoxic conditions (Supplementary Fig. 7d). These results indicate that hypoxic tumour-CAF co-cultures retain, and in the case of CXCL10 enhance, the production of immune-attractant chemokines despite reduced immune infiltration. Together with the increased ECM remodelling observed under HHR conditions, these findings are consistent with a model in which hypoxia-associated, CAF-driven ECM remodelling contributes to impaired immune cell infiltration independently of chemokine availability. Once reduced immune infiltration was established by flow cytometry, we next assessed whether these effects were reflected in the spatial organization of immune cells using imaging-based analyses in both HHR/PLR models and HGSC patient tumours. Multiplex immunofluorescence (IF) analysis of HGSC tumours with high or low immune infiltration revealed distinct spatial organization of CD45⁺ immune cells (green) relative to cytokeratin⁺ tumour cells (magenta) and vimentin⁺ stromal compartments (red), with increased immune infiltration observed in immunologically hot tumour areas (Supplementary Fig. 8a). To benchmark the engineered 3D models, dissociated HGSC tumour tissues were cultured under PLR or HHR conditions and challenged with autologous PBMCs. Confocal z‑stack imaging for CD45 (green), cytokeratin (magenta), and FAP (red) demonstrated significantly impaired immune infiltration in HHR compared to PLR models, recapitulating spatial features observed in immunologically cold tumour areas (Supplementary Fig. 8b). Altogether, these data reveal that the hypoxia-induced CAF-driven ECM remodelling preserved in the HHR model is associated with impaired immune infiltration and further highlight the feed-forward loop effect of intratumoural hypoxia on CAFs influencing immune evasion mechanisms.

### Galunisertib reduces hypoxia-induced CAF-driven TGF-β signalling and collagen I expression in the HHR model

In CAFs, TGF-β signalling plays a crucial role in driving collagen I secretion and ECM remodelling^35,36^. Intratumoural hypoxia upregulates TGF-β and its receptors, leading to increased SMAD activation, promoting ECM remodelling through activation of CAFs, stimulating the expression of collagen (COL1) and lysyl oxidase (LOX) genes, and repression of MMP genes, aiding immune escape^35,37^.

**Fig. 5:**
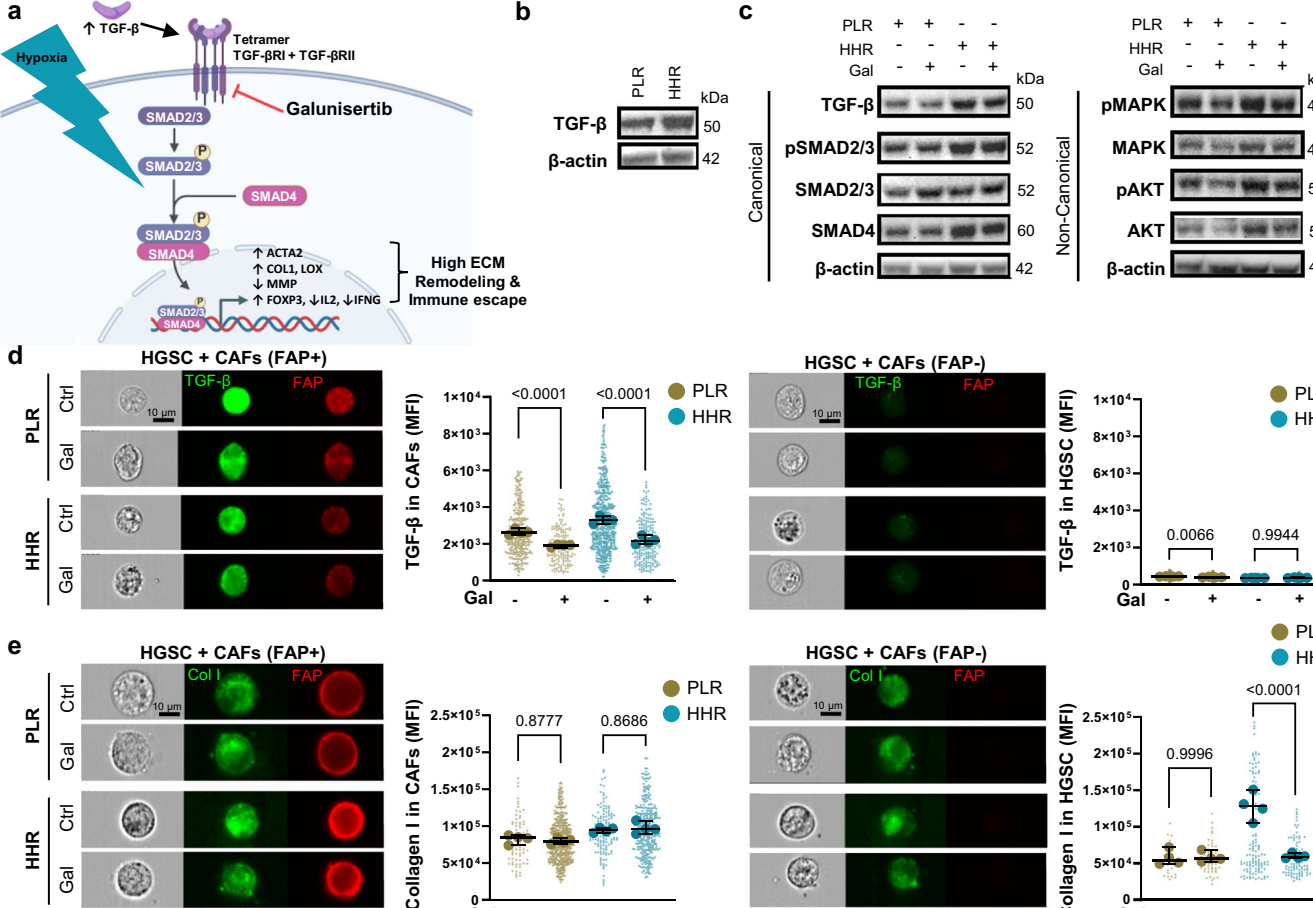
Preincubation with galunisertib reduces hypoxia-induced CAF-driven TGF-β signalling and collagen I expression recapitulated in HHR model. **a** Schematic of TGF-β signalling pathway involved in ECM remodelling in the hypoxic TME and the mechanism of action of the TGF-βRI inhibitor, galunisertib. Created in BioRender. de la Puente, P. (2026) https://BioRender.com/08y0mue. **b** Representative images of TGF-β expression detected by western blot in co-culture. *n* = 3. **c** Expression of signalling molecules involved in canonical (left) and non-canonical (right) TGF-β signalling pathways in the presence or absence of galunisertib (Gal) detected by western blot in co-culture. *n* = 3. **d** Representative images of intracellular TGF-β (green) and **e** collagen I (green) in imaging flow cytometry in co-culture of CAFs (FAP + , red) and HGSC (FAP−), and quantification of green mean fluorescence intensity (MFI). Scale = 10 μm. **d**, **e** Median ± CI95%, one-way ANOVA. Data are represented as superplots with biological replicates shown as larger, darker symbols (*n* = 4 PLR/HHR cultures from different days) and technical replicates as smaller, lighter symbols (*n* = 20 individual cells/images minimum). Source data are provided as a Source Data file.

To prevent the hypoxia-induced dysregulated ECM remodelling involved in impaired immune infiltration in our 3D model, we pharmacologically targeted TGF-β signalling using preincubation with a model drug galunisertib (Gal), a small-molecule inhibitor of the TGF-β receptor I (TGFβRI) kinase (Fig. 5a). Higher expression of TGF-β in HHR compared to PLR was validated by western blot in co-cultures (Fig. 5b and Source Data 2a), which aligned with the transcriptomic data from Figs. 1f and 2f. When monocultures were interrogated, it was found that CAFs were primarily responsible for the TGF-β upregulation (Supplementary Fig. 9a and Source Data 2a). Pre-incubation with Gal induced downregulation of downstream canonical signalling (TGF-β, pSMAD2/3, SMAD4) and non-canonical pathways (pMAPK and pAKT) when compared to the untreated control in both PLR and HHR in co-cultures (Fig. 5c and Source Data 2b) and more strongly in the CAFs than the HGSCs cells in monocultures (Supplementary Fig. 9b and Source Data 2b). Intracellular staining using imaging flow cytometry quantitatively validated that pre-incubation with Gal significantly reduced TGF-β expression in both PLR and HHR in co-cultures and confirmed CAFs as the main contributors to intracellular TGF-β expression (Fig. 5d and Supplementary Fig. 9c). Monocultures further supported the CAFs robust implication in intracellular TGF-β expression with significant downregulation by Gal treatment compared to KURAMOCHI (Supplementary Fig. 9d). Of note, while western blot analysis reflects total TGF-β levels in whole 3D construct lysates including cellular and ECM-associated protein, imaging flow cytometry represents intracellular TGF-β expression.

In light of the relationship between TGF-β and collagen type I, we ascertained whether galunisertib treatment affected collagen expression and its deposition. Intracellular collagen staining aligned with the results of intracellular TGF-β expression where Gal significantly decreased intracellular collagen in HHR when compared to untreated control in HGSC in co-culture, although did not affect the intracellular collagen expression in CAFs nor monocultures (Fig. 5e and Supplementary Fig. 9e). Furthermore, cellular and secreted collagen type I expression was evaluated by IHC staining and showed that pre-incubation with galunisertib significantly reduced collagen deposition in HHR in CAF monocultures and their co-cultures with KURAMOCHI, however, increased in PLR co-cultures (Supplementary Fig. 9f). Collectively, our results demonstrate that preincubation with galunisertib robustly reduces the hypoxia-induced CAF-driven TGF-β signalling and decreases collagen I deposition recapitulated in HHR models, opening ways to normalise the dysregulated ECM remodelling in HGSC tumours.

### Galunisertib attenuates the hypoxia-induced CAF-driven impaired immune infiltration

**Fig. 6:**
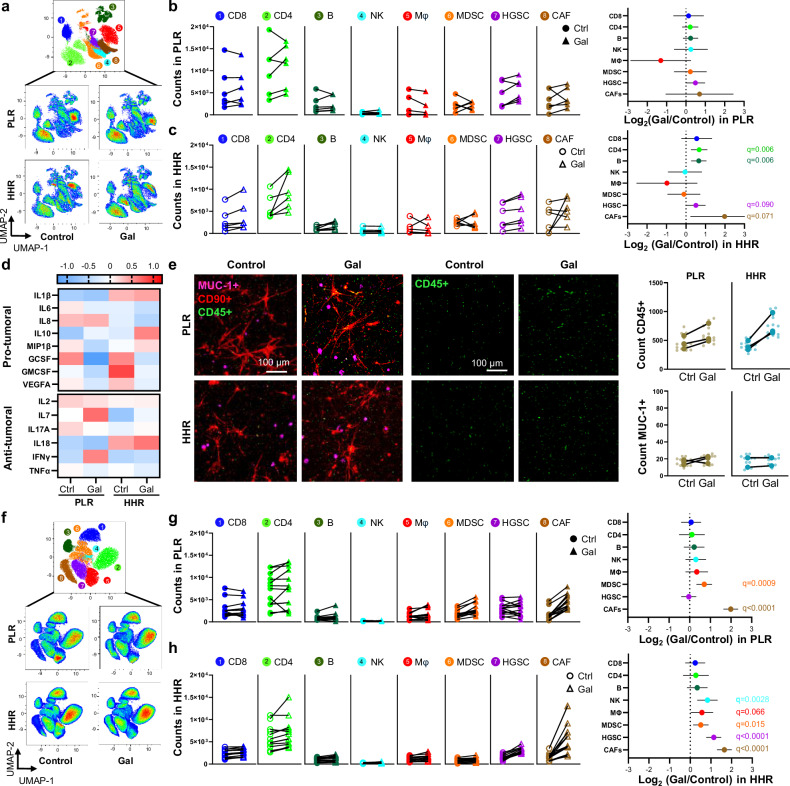
Preincubation with galunisertib attenuates the hypoxia-induced CAF-driven impaired immune infiltration. **a** UMAP plot, density plots, and **b**, **c** donor-paired dot plots representing the number (counts) of infiltrated PBMCs cell types (CD3 + , CD4 + , CD8 + T cells, Mφ (CD14 + ), NK (CD56 + ), B (CD19 + ) and MDSC (CD11b + )), and HGSC cells from dissociated tumours in co-culture with CAFs inside PLR (b) or HHR (**c**) in the presence or absence of galunisertib (Gal) in dimensionality reduction analysis by flow cytometry. n = 6 PBMCs from different donors. Forest plots show the log_2_ fold changes ± 95% confidence intervals of Gal/Control in PLR or HHR. **d** Heatmaps showing the expression of secreted pro-tumoural and anti-tumoural cytokines in HGSC patient samples. Median of z-score for each analyte. *n* = 6. **e** Confocal microscopy images of z-stack view as a maximum projection of infiltration of PBMCs (CD45 + , green) into the scaffold with KURAMOCHI (MUC-1 + , magenta) and CAFs (CD90 + , red) in the presence or absence of galunisertib and quantification of the number of CD45+ and HGSC. Scale = 100 μm. Data are represented as donor-paired superplots with biological replicates shown as larger, darker symbols (*n* = 3 PLR/HHR cultures from different PBMC donors) and technical replicates as smaller, lighter symbols (*n* = 4). **f** UMAP, density plots, and **g** donor-paired dot plots showing the number (counts) of infiltrated PBMCs phenotypes and KURAMOCHI and CAFs inside the scaffold in the presence or absence of galunisertib in dimensionality reduction analysis by flow cytometry and forest plots representing log_2_ fold changes ± 95% confidence intervals of Gal/Control in PLR or **h** HHR. *n* = 13 PBMCs from different donors. **b**, **c**, **e**, **g**, **h** linear mixed-effect model analysis with Benjamini–Hochberg FDR. Source data are provided as a Source Data file.

To further explore whether galunisertib could reverse the hypoxia-induced CAF-driven impaired immune infiltration, PLR and HHR models were preincubated for 3 days in the absence or presence of galunisertib and infiltration studies were performed as previously described (Supplementary Fig. 10a). Autologous PBMCs were challenged to infiltrate the 3D matrix with primary HGSC cells from dissociated tumours co-cultured with CAFs. A broader spread of UMAP clusters was identified when using autologous PBMCs from HGSC patients, revealing underlying biological variability or inter-patient heterogeneity that could be related to variable treatment response or divergence in the cellular landscape (Fig. 6a, Supplementary Fig. 10b and Table 1). Treatment with galunisertib significantly increased CD4^+^ cells and B cells in HLR, while the effect was smaller in the rest of the immune cells and in PLR (Fig. 6b, c and Supplementary Table 1). No significant model x treatment interaction was observed (Supplementary Fig. 10c and Supplementary Table 1), demonstrating model-independent drug activity rather than hypoxia/ECM-specific enhancement. Analysis of the secreted tumour milieu revealed a higher expression of pro-tumoural cytokines such as IL-1β and growth factors such are GM-CSF and VEGF-A in HHR compared to PLR and no significant changes in anti-tumoural cytokines based on the oxygen conditions except for IL-18 (Fig. 6d). Preincubation with galunisertib decreased the expression of pro-tumoural growth factors (G-CSF, GM-CSF, and VEGF-A) in PLR and HHR but also enhanced pro-tumoural IL-10 and MIP-1β and anti-tumoural IL-18 cytokines.

**Table 1:** Demographics of patients used in the study

Demographics	Characteristics	*N* (Total *N = *22)	% (Total = 100%)
Age (mean ± SD)	64.77 ± 13.158		
Race/ethnicity	White/Caucasian/Non-Hispanic	22	100
Diagnostics	HGSC	18	81.8
	Serous borderline	3	13.64
	Adnexa Adenocarcinoma	1	4.54
Stage	IIIC	8	36.36
	IV	3	13.63
	Unspecified	11	50.0
Treatment	Untreated	6	27.27
	Platinum/taxane-based	5	22.72
	Platinum/taxane-based + other	7	31.81
	Unspecified	4	18.18
Treatment response	Complete	1	4.54
	Partial	7	31.81
	Unspecified	14	63.63

Complementary experiments were performed in the KURAMOCHI cell line co-cultured with CAFs with infiltrating PBMCs to validate the observed effects of galunisertib on immune infiltration while minimising confounding effects of inter-patient heterogeneity by confocal imaging and flow cytometry. Confocal microscopy results showed that preincubation with Gal significantly increased the infiltration of CD45^+^ immune cells into the 3D matrix under PLR and HHR having significant model x treatment interaction. The cancer signal showed modest, non-significant shifts in both PLR and HHR and no significant interaction (Fig. 6e, Supplementary Fig. 11a, Supplementary Table 1). Similar directional effects were observed in CD45^+^ T cell infiltration in KURAMOCHI monocultures, although lacking statistical significance while cancer cells showed a trend toward a hypoxia-enhanced drug effect with higher interaction (Supplementary Fig. 11b).

Immunophenotyping results by flow cytometry in KURAMOCHI cells co-cultured with CAFs showed that galunisertib pre-treatment significantly increased the infiltration of MDSCs in PLR and NK cells, macrophages and MDSCs in HHR (Fig. 6f–h and Supplementary Fig. 11c, Supplementary Table 1). In KURAMOCHI monocultures the treatment with Gal significantly increased the infiltration CD8^+^, CD4^+^, B cells, NK cells, Mφ and MDSC in HHR with more modest but still significant effects in PLR in the same immune cell populations, except MDSC, suggesting hypoxia-permissive environment rather than a cell-type restricted effect (Supplementary Fig. 11e–g, and Supplementary Table 1). Nonetheless, no cell population showed a significant model x treatment interaction in either condition (Supplementary Fig. 11d, h). These results suggest that preincubation with galunisertib attenuates the impaired immune infiltration of adaptive immune cells (CD4+ and B cells) in HGSC patients and innate lineage (NK, Mφ and MDSC) in healthy PBMCs in an oxygen/ECM-independent manner in this model. To further determine whether the enhanced immune infiltration by galunisertib aligns with an increased cytotoxic effect, confocal microscopy with live/dead cell staining was performed and verified a significantly higher cell death in HHR in the presence of Gal compared to the control (Supplementary Fig. 11i). Once demonstrated that hypoxia-induced CAF-driven TGF-β signalling and collagen I expression is associated with impaired immune infiltration, and that these effects can be disrupted by TGF-β inhibition, we next asked whether these relationships are preserved within the spatial architecture of human HGSC tumours. Immunohistochemistry was performed on tissue sections from three HGSC patients, staining for CD8⁺ T cells, collagen I, TGF‑β, Masson’s trichrome, and H&E (Supplementary Fig. 12a–c). For each patient, two regions of interest (ROIs) with low CD8⁺ T‑cell abundance and two ROIs with high CD8⁺ T‑cell abundance were quantified, except for HGSC patient 1, which exhibited an immunologically deserted tumour phenotype and lacked regions with high CD8⁺ infiltration. Across samples, regions with low CD8⁺ T‑cell presence were associated with significantly higher collagen deposition compared to immune‑infiltrated regions. Although no statistically significant difference in TGF‑β expression was observed, an increasing trend in TGF‑β levels was detected in collagen‑rich, immune‑excluded areas (Supplementary Fig. 12d). These observations are consistent with an association between ECM deposition and immune exclusion in HGSC tumours.

### Galunisertib improves immune infiltration in native ovarian decellularized ECM

**Fig. 7:**
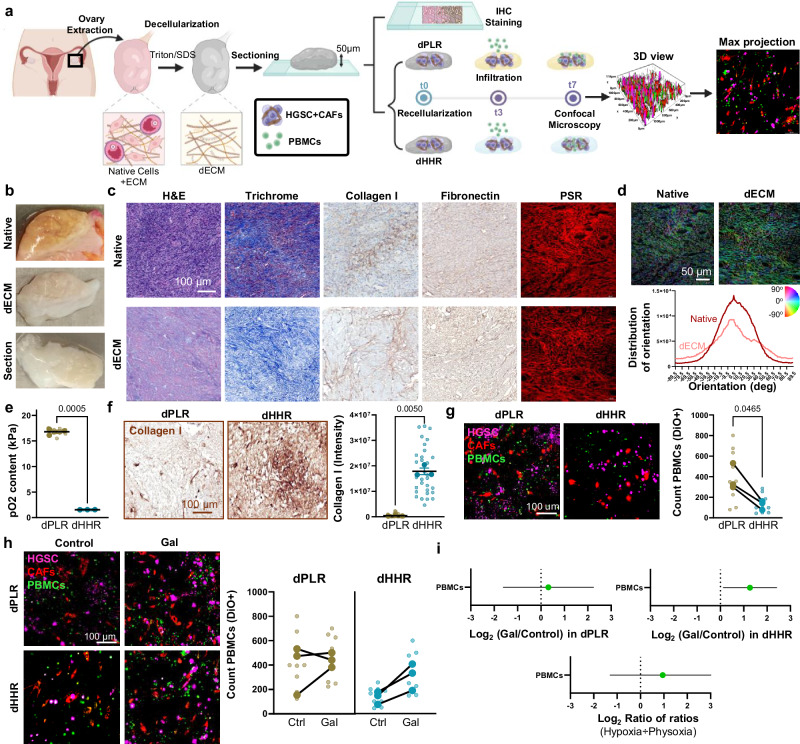
Galunisertib promotes partial recovery of impaired immune infiltration in ovarian native dECM. **a** Schematic representation of decellularization of human ovaries and subsequent recellularization in dPLR (decellularized physoxia-low remodelling of ECM) and dHHR (decellularized hypoxia-low remodelling of ECM) models and immune infiltration studies. Created in BioRender. de la Puente, P. (2026) https://BioRender.com/hfvvr5n. **b** Representative images of the human ovary before (top) and after decellularization (middle) and transversal section of dECM (bottom). **c** Representative images of H&E, Masson’s trichrome, collagen I, fibronectin and picrosirius red (PSR) staining of ovarian native tissue and dECM. Scale = 100 μm. Same results were identified for 3 independent tissues. **d** Quantification of the distribution of orientation of collagen fibres in PSR in native ovary and dECM. *n* = 2. **e** Oxygen levels measured inside the re-cellularized dPLR and dHHR model by an optical sensor at day 3. **f** Quantification and IHC images for collagen I in re-cellularized dECM at day 3. Scale = 200 μm. **g** Confocal microscopy images and the quantification of the number of infiltrated PBMCs (green) into the dECM recellularized with KURAMOCHI (magenta) and CAFs (red) in the absence and **h** in the presence of galunisertib. Scale = 100 μm. Donor-paired superplots *n* = 3 (3 ROIs). **i** Forest plots showing log_2_ fold changes ± 95% confidence intervals of Gal/Control and log_2_ ratio of ratios of interactions model x treatment (hypoxia/physoxia). **e**, **f** Mean ± SD, Welch’s *t* test two-tailed. **g** Paired *t* test two-tailed; **h** linear mixed-effect model analysis with Benjamini–Hochberg FDR. **e**, **f** Data are represented as superplots with biological replicates shown as larger, darker symbols (*n* = 3 PLR/HHR cultures from different days) and technical replicates as smaller, lighter symbols (*n* = 3 for oxygen and 10 for collagen). **g**, **h** Data are represented as donor-paired superplots with biological replicates shown as larger, darker symbols (*n* = 3 PLR/HHR cultures from different PBMC donors) and technical replicates as smaller, lighter symbols (*n* = 4). Source data are provided as a Source Data file.

To evaluate whether key observations from the plasma-derived 3D system are preserved in a native ECM context, we employed a complementary 3D culture platform based on decellularized human ovarian ECM tissue (dECM). Both platforms were cultured under physoxic or hypoxic conditions, enabling assessment of oxygen- and ECM-dependent effects across systems. The decellularized ovary matrices were re-cellularized with KURAMOCHI and CAFs while exposed to physiologically relevant oxygen levels and then PBMCs were challenged to infiltrate (Fig. 7a). Decellularization was confirmed by gross morphological assessment where decellularized tissue became translucent and whitish, consistent with removal of cellular material (Fig. 7b). Histological assessment included confirmation of loss of nuclei by H&E staining and preservation of ECM by Masson’s trichrome, collagen I, fibronectin and picrosirius red (PSR) staining in the decellularized matrix compared to native tissue (Fig. 7c). Collagen fibres orientation in PSR staining showed that dECM and native tissue had similar alignment with dominant orientation in 0^o^ and displayed moderate dispersion of the peaks consistent with structurally organised but not rigid collagen network in native ovary (Fig. 7d). After corroboration of cell removal and ECM integrity, we validated that physiological oxygen levels and ECM remodelling were mimicked in these models at day 3. The partial pressure of oxygen (pO_2_) measurement inside the re-cellularized sectioned tissue revealed ~16.3 kPa and 1.5 kPa levels when grown in 21% and 1.5% O_2_, respectively (Fig. 7e), and significantly higher collagen I deposition was observed in hypoxia when compared to physoxia (Fig. 7f). Therefore, we refer to them as, “decellularized physoxia-low remodelling of ECM” model (dPLR) and “decellularized hypoxia-high remodelling of ECM” model (dHHR). We further verified by confocal microscopy that when immune cells were added onto these models, a significantly impaired immune infiltration in dHHR compared to dPLR was found (Fig. 7g), consistent with the results of the patient-derived 3D models in Fig. 4. Then, preincubation with galunisertib increased the immune infiltration in hypoxia (*q* = 0.12) with smaller, non-significant effect in physoxia in alignment with our previous results (Fig. 7h, i and Supplementary Table 1). The model×treatment interaction analysis indicated a larger hypoxia/ECM effect but did not reach FDR significance with three patients. Replicate-aware plots confirm parallel control-galunisertib shifts within donors, and ICCs document replicate reliability per condition (Supplementary Table 1). Together, these complementary models enabled evaluation of HGSC tumour-immune interactions in both a scalable plasma-derived matrix and a biologically complex native ovarian ECM.

In summary, our findings show that the hypoxia-induced CAF-driven impaired immune infiltration can be recapitulated in the patient-derived tumour-immune models, which were also suitable for dissecting targeting opportunities via TGF-β signalling and collagen by galunisertib, turning immunologically cold HGSC tumours into hot ones in this model system.

## Discussion

In this study, we bioengineered patient-derived tumour-immune models that capture biologically representative microenvironments, enabling translational investigation of tumour-stroma-immune dynamics during tumour initiation and progression. By integrating physiologically relevant oxygen levels and ECM cues, these models overcome fundamental limitations of existing preclinical immune models, which fail to reproduce key tumour characteristics such as physoxia, hypoxia, and stromal-driven matrix remodelling. Specifically, our approach reconstructs the oxygen and ECM landscapes of physoxic early-stage and hypoxia-induced ECM remodelling in advanced HGSC. The platforms incorporate cancer cells co-cultured with CAFs within three-dimensional matrices engineered using autologous patient plasma or decellularized human ovaries, while immune cells are either embedded as multicultures or challenged to infiltrate the matrices. Together, these features establish a grounded framework to interrogate how oxygen and stromal remodelling shape immune exclusion and function in HGSC.

This study demonstrates that hypoxia-induced CAF-driven ECM remodelling impairs immune infiltration, which is in line with the literature^38–41^, while using physoxic ovarian environments as a better control for modelling the gradient between the tumour initiation site towards advanced hypoxic tumours.

Tumours initiate in physoxic regions and progress into hypoxia while remaining surrounded by healthy tissue^26,42^, which has been frequently overlooked in vitro in favour of atmospheric oxygen levels typically used in standard cell culture^43–45^. As an example of the relevance of physoxic oxygen levels in the ovaries, it has been demonstrated that lower oxygen tensions in in vitro cultures yield higher follicle viability and quality than 20% O_2_ tension^46^. Advanced-stage tumours are also characterised by an aberrant stiffer ECM, with a distinct composition from normal tissue where tumour initiates^26,47^. When cells are cultured on or within ECM with composition or abundance that does not reflect their native or disease-specific environment, they can exhibit artificial or misleading behaviours^16,17,19^. Incorporating these physiologically relevant characteristics into in vitro models is essential to faithfully uncovering context-specific mechanisms driving immune evasion^48,49^. Matrix-based platforms such as those containing collagen or basement membrane extracts have enabled the evaluation of tumour invasion and screening of therapies^40,50,51^. However, they neglect either the incorporation of stromal or immune compartments, or oxygen–induced shifts in matrix composition and immune cell distribution or inaccurate comparisons of hypoxia to normoxia. Decellularized omental metastasis importantly demonstrated that the ECM educates an immunoregulatory tumour macrophage phenotype^19^, but this study was performed under a normoxic environment. To the best of our knowledge, these human-relevant tumour-immune models uniquely integrate oxygen conditions of the hypoxic advanced-stage TME alongside the physoxic milieu of tumour initiation, while integrating CAFs and patient-derived materials, including tumour biopsies, PBMCs, and plasma, enabling interrogation of HGSC TME interactions that are largely inaccessible in existing systems. Critically, mixing patient-derived plasma with collagen to grow tumours enhances the physiological relevance of our in vitro models by incorporating systemic, patient-specific factors such as cytokines, metabolites, and growth factors^30,31^. This approach supports precision-based applications by better capturing inter-individual variability in tumour behaviour and therapeutic response^52^. Alternatively, to account for native ECM composition and architecture while reducing intra-assay variability due to the plasma matrix, decellularized ovaries were used as a validation platform, highlighting the important contribution of oxygen levels and CAF-driven ECM remodelling to immune evasion. While both platforms support oxygen-dependent modelling, the plasma-derived system enables broader experimental scalability, whereas decellularized ovarian ECM provides a native validation context constrained by tissue availability. Overall, these models are versatile tools that could be used to explore other cancer types as they are easily modifiable to recreate specific biological, biophysical, and biomechanical properties of other organs^30,31^.

Here we demonstrated that the PLR and HHR 3D models benchmark the molecular fingerprints of the native tumour tissue by conserving the enriched gene signatures of the tumour molecular subtype and key microenvironmental features such as hypoxia, matrisome and TGF- β signalling pathway. Mechanistically, we identified that the HHR model captures classical hypoxic features at transcriptomic levels and via functional read-outs. We functionally characterise that intratumoural hypoxia induced CAF activation leading to aberrant ECM remodelling through overexpression of collagen and other core matrisome genes driven by TGF-β signalling, and densely packed and highly aligned collagen fibres with stiffer matrix mimicking key features of advanced HGSC tumours^47,48^. Other studies also supported that hypoxia induces collagen deposition in human and murine omental metastasis^53^. Remarkably, the presence of PBMCs in the multi-culture with cancer cells and CAFs, decreased the hypoxia-induced CAF-driven collagen I expression which correlated with an increased expression of MMP-9 similar to studies in other cancers^54,55^, highlighting the importance of tumour-stroma-immune interactions^54^ in the hypoxic environment. Based on functional measurements, we have shown that intratumoural hypoxia acts as a friend and foe in modulating immune responses, increasing the effector functions of CD8^+^ T cells with enhanced activation, cytotoxicity, and killing, while simultaneously promoting immunosuppressive cells. Similar findings were observed in other studies in HGSC patients where intratumoural hypoxia induced immunosuppressive cell types^56,57^, but also a higher presence of activated CD8^+^ T cells in the TME^21,58,59^. TILs, particularly CD8^+^ T cells, existed along a functional spectrum simultaneously displaying features of activation, cytotoxicity, and exhaustion, reflecting a dynamic balance between ongoing tumour engagement and progressive dysfunction, which has been featured as a state predictive of immunotherapy response^60,61^. In recent years, CAFs are emerging as important regulators of the immune cell continuum inducing immunosuppression and ovarian cancer progression^62,63^. We showed that intratumoural hypoxia influenced cancer-stroma-immune interactions through the collagen pathway where CAFs were the main contributors which falls in line with other studies^53,63^. Strong communication probabilities were detected between the collagen I ligand and integrins, CD44, and syndecan receptors, which have been previously correlated with an immunosuppressive TME and chemoresistance in different cancers including ovarian cancer^63–66^. Even though hypoxia has been reported to suppress immune-attractant chemokine production in some contexts, our data indicate that in HGSC tumour-CAF co-cultures in hypoxia does not diminish, and may enhance, CXCL10 secretion. This suggests that physical and biochemical properties of the remodelled ECM, rather than loss of chemotactic signalling, represent a dominant barrier to immune infiltration in advanced-stage disease. Consistent with the spatial organization observed in HGSC tumours with high or low immune infiltration areas, imaging analysis revealed that the engineered PLR and HHR models recapitulate immune‑infiltrated and immune‑excluded architectures, respectively, supporting their relevance for studying spatial determinants of immune exclusion. Although limited in sample size, the observed association between collagen-rich regions and reduced CD8⁺ T-cell infiltration in HGSC patient tissues supports the clinical relevance of the CAF-driven ECM remodelling mechanisms identified in vitro. Infiltration studies demonstrated that intratumoural hypoxia alone is not responsible for immune evasion but rather it triggers CAF-driven ECM remodelling, which altogether impairs immune infiltration. Hypoxia induced the activation of CAFs leading to increased collagen and TGF-β expression, confirming CAFs as main orchestrators of TGF-β signalling and its correlation with hypoxia through HIF-1α as previously described^35^. This highlights the complexity of the TME and the need for sophisticated models capturing the feed-forward loop between oxygen content and CAF activation and its influence on immune dynamics.

Several strategies have been developed to target TGF-β signalling to overcome ECM remodelling and immune resistance^67^. Here, we used Galunisertib (Gal) as a model drug, which is a small-molecule inhibitor of TGFβRI phosphokinase activity^68,69^ and has been used in several preclinical models and clinical trials with promising results in different cancers such as hepatocellular carcinoma, myelodysplastic syndrome, rectal, and non-small cell lung cancer^67–69^; although with limited efficacy in recurrent glioblastoma^70^ and metastatic pancreatic cancer^71^. We selected Gal due to its anti-tumour activity having simultaneous effects on both CAFs and immune cells, by reducing CAF activation and promoting antifibrotic effects, and by alleviating immune suppression with higher immune infiltration and activation^69,72,73^. We acknowledge that Gal can present several side effects such as cardiotoxicity and skin toxicity, but they could be overcome by careful dosage, pulsatile therapy or targeted therapy^74^. We mechanistically confirmed the anti-fibrotic potential of Gal decreasing the expression of TGF-β signalling molecules and collagen I in hypoxia-induced CAFs similar to others^69,75,76^. Although galunisertib directly inhibits TGF‑β receptor I rather than ligand production, treatment was associated with reduced intracellular TGF‑β levels. We hypothesise that this effect reflects disruption of TGF‑β–dependent feedback loops and autocrine signalling mechanisms that normally sustain ligand expression and intracellular availability^37,77,78^. In addition, differences between whole‑lysate western blotting and intracellular imaging measurements likely reflect distinct cellular versus extracellular pools of TGF‑β within the 3D constructs. Together, these observations are consistent with context‑dependent regulation of TGF‑β signalling following receptor inhibition. In contrast, under physoxic conditions Gal treatment was associated with increased collagen I levels in tumour–CAF co-cultures, indicating a context-dependent response to TGFβRI inhibition. We hypothesise that under hypoxic conditions collagen production is more tightly coupled to TGF-β–dependent signalling through HIF-1α, rendering it more sensitive to galunisertib treatment, whereas under physoxic conditions compensatory pro-fibrotic responses may offset TGFβRI, resulting in a net increase in collagen expression^35,79^. Together, these findings suggest that oxygen tension modulates fibroblast responses to TGF-β pathway inhibition and highlight the importance of microenvironmental context when interpreting anti-fibrotic therapeutic effects. Importantly, parallel to other studies^80,81^, our data indicate that pre-treatment with Gal attenuates the hypoxia-induced CAF-driven impaired immune infiltration of CD45^+^ immune cells in our plasma 3D models mainly the adaptive immune cells in the HGSC-patient-derived model and innate lymphoid and myeloid lineage in healthy PBMCs using cell lines within the matrices. Even under physoxic oxygen tension and low ECM remodelling, the drug had detectable immune-modulatory activity, but effect sizes were smaller than under hypoxia and high ECM remodelling across all populations acting through oxygen/ECM-agnostic mechanisms. The differences in the responses between PBMCs from healthy donors and HGSC patients can be correlated with acquired immune resistance in primary cells and an altered cell behaviour due to previous chemotherapeutic and immunotherapeutic clinical exposure in the HGSC patients^82^. Similar trends were observed in decellularized human ovaries, although the limited sample availability constrained the number of replicates and immunophenotyping studies.

We acknowledge that our studies might have certain limitations. We used fully characterised reprogrammed HGSC-CAFs generated in our lab^83^ that resemble primary CAFs while exhibiting high CAF and ECM/matrisome-scoring which were linked to the mesenchymal subtype with the worst prognosis in ovarian cancer^32^. However, future studies should confirm the results using primary CAFs, bearing in mind they constitute a heterogeneous population that can lead to pleiotropic responses and present limited cell passage number and growth rate^84^. We investigated the role of hypoxia in inducing ECM remodelling, but we did not address how ECM remodelling affects hypoxia, even though their relationship is reciprocal^85^. The use of non-HLA or donor-matched PBMCs and CAFs may present a potential concern with alloreactivity, but due to the short duration window of the in vitro experiments (3–7 days) we expect minor signs of graft versus host disease, considering those have been identified within 4 weeks of PBMC implantation in humanised mice^86,87^. Therefore, we mitigated this concern by leveraging autologous PBMCs and tumour cells from HGSC patients in part of our studies with the 3D models made from matching plasma to avoid the graft-vs-tumour rejection effects^88^. While the present study does not directly compare plasma composition between healthy donors and HGSC patients, our prior work using human plasma-derived 3D cultures demonstrated comparable fibrinogen content and baseline cytokine profiles between healthy and cancer patient plasma^31^ supporting the robustness of plasma-based matrices and minimising the likelihood that plasma source alone confounds ECM formation or immune infiltration outcomes. Moreover, while the HGSC patient samples share some global transcriptional features, their internal diversity leads to more diffuse projections in low-dimensional space. We note that all participants were White/Caucasian non-Hispanic women, due to higher incidence and sample availability; however, samples from diverse patients should be studied to consider the race/ethnicity factor and prevent health disparities^89^. Another limitation is the lack of spatio-temporal organization of the cells inside of the 3D model through compartmentalisation, which could be implemented using a tumour-on-a chip model as previously described^90^. We also acknowledge that the physoxic O_2_ level in the dECM was higher than in physoxic tissues due to the low thickness of the decellularized matrix for imaging purposes, therefore increasing the thickness of the dECM or using an incubator with controlled O_2_ levels could better recapitulate physoxic levels in this model. In the future, more stromal cell types, such as endothelial cells or adipocytes, should be incorporated into the model to better mimic HGSC-TME. Further studies should also investigate the polarisation of tumour-associated macrophages (TAMs) to decipher specifically how intratumoural hypoxia affects the innate immune response. Furthermore, different ECM-targeting drugs, or combination therapy of Gal, immunotherapy, and hypoxia-targeting agents should be explored but this was outside of the scope of the current study^67,91^.

In summary, we have shown that recapitulation of the hypoxic tumour levels in advanced stage in comparison to the physoxic oxygen levels in tumour initiation as a more physiologically relevant control are essential to gain insights into tumour-stroma-immune interactions and model immunologically cold HGSC tumours. Our findings underscore the pivotal role of hypoxia in driving CAF-driven ECM remodelling, which contributes to immune exclusion sustaining the immunologically cold nature of HGSC tumours. Importantly, our translationally relevant models may assist in identifying therapeutic targets aimed at disrupting stromal barriers and promoting immune infiltration, offering a path toward transforming immunologically cold tumours into hot and responsive ones.

## Methods

### Cell lines and cell culture

KURAMOCHI (RRID:CVCL1345), OVSAHO (RRID:CVCL_3114), OVCAR-3 (ATCC HTB-161), OVCAR-8 (RRID:CVCL_1629), CAOV3 (ATCC HTB-75), OV81.2 and HEYA8 (RRID:CVCL_8878) cell lines were used as models for HGSC and cultured in DMEM /Ham’s F-12 media (1:1) supplemented with 10% (V/V) of fetal bovine serum (FBS) 100 U/ml penicillin, and 100 mg/ml streptomycin. OVCAR-8, CAOV-3, HEYA-8, and OV81.2 cell lines were a kind gift from Dr. Analisa Difeo and OV81.2 was generated from HGSC patient’s ascites re-engrafted into immunodeficient mice^92^. Ovarian CAFs were developed in our laboratory by reprogramming primary human uterine fibroblasts (ATCC PCS-460-010) with conditioned media from KURAMOCHI cells^83^. The reprogrammed CAFs were phenotypically and functionally validated demonstrating an activated CAF-like state characterised by enhanced proliferative, secretory, contractile, and ECM-remodelling properties relative to normal fibroblasts, as well as the ability to promote tumour progression and drug resistance via non-vesicular paracrine signalling.

### Human tissue and primary cells

Tumour biopsy and peripheral blood samples from ovarian cancer patients undergoing a cytoreductive surgery were provided by the Sanford USD Medical Center, Sioux Falls, SD, or the University of Iowa, Iowa City, IA. Paraffin-embedded HGSC tissues were provided by Sanford Biobank, Sioux Falls, SD. Informed consent was signed by all subjects with approval from the Sanford Health Institutional Review Board (STUDY00001315) or University of Iowa’s Institutional Review Board (#201809807), respectively and in accordance with the Declaration of Helsinki. Peripheral blood from de-identified healthy female volunteers was provided by the Sanford Blood Biobank. The findings in this study apply only to female sex. Only female samples were considered, as ovarian cancer affects only female reproductive tract. Ovarian tumour tissue was stored in MACS® tissue storage solution and used fresh or frozen in freezing solution composed of FBS with 10% (V/V) dimethyl sulfoxide. Patient demographic and therapeutic treatment characteristics are included in Table 1. Tumour tissues were dissociated using MACS® Tissue Dissociation kit following the manufacturer’s instructions. Primary cells were cultured in DMEM/Ham’s F-12 (1:1) supplemented with 10% of FBS and 100 U/ml penicillin and 100 μg/ml of streptomycin. PBMCs were isolated from peripheral blood using Leucosep round-bottom centrifugation tubes filled with Ficoll®-Paque Plus density gradient media PBMCs were cultured in CTS™ OpTmizer™ T Cell Expansion SFM supplemented with OpTmizer™ T-Cell Expansion Supplement and 2 mM L-glutamine and, if needed, stimulated in RPMI media with eBioscience™ Cell Stimulation Cocktail (plus protein transport inhibitors, 500×) for 5 hours. In the co-culture of PBMCs with primary tumour cells or KURAMOCHI, T cell expansion media was mixed with DMEM/Ham’s F-12 (1:1). Ovaries from human female cadavers were extracted post-mortem at the Dakota Lions and Health Tissue Donation organization and stored in MACS® tissue storage solution until processed for decellularization.

### Bioengineering PLR and HHR 3D models

Patient-derived 3D models were bioengineered by crosslinking of human plasma fibrinogen into fibrin^30,31^. Specifically, human plasma isolated from healthy female donors or HGSC patients was mixed with Cultrex® collagen I (final concentration 2 mg/ml) and trans-4-(aminomethyl)cyclohexanecarboxylic acid (T-AMCHA) as a stabiliser, and CaCl_2_ as a crosslinker in a final ratio 4:4:1:1, respectively. For HGSC patient-derived models, dissociated primary tumour tissues (10–25 mg) were embedded in 3D scaffolds generated using autologous, patient-matched plasma and PBMCs, thereby preserving patient-specific tumour-immune interactions. Whole tumour digests were used without further cellular fractionation to preserve native tumour heterogeneity; cellular input was standardised by tissue mass and uniform dissociation protocols rather than by predefined tumour cell numbers. In contrast, for models constructed with immortalised cancer cell lines (2 × 10^5^ cells/ml), plasma and PBMCs from healthy donors were used, as autologous immune components corresponding to the original tumour donors are not available for established cell lines, and the use of unrelated cancer patient immune materials would introduce uncontrolled allogeneic immune effects. Unless otherwise noted, HGSC cell lines or primary HGSC cells were cultured inside of the 3D scaffolds in monoculture or co-culture with CAFs (2 × 10^5^ cells/ml). PBMCs (4 × 10^5^ cells/ml) were either added on top of the scaffolds and challenged to infiltrate the matrix or multi-cultured inside the 3D scaffolds. Media was added on top of the scaffold to avoid drying and to penetrate into the matrix to allow cell growth. The biomechanical properties of the model such as height, stiffness, crosslinking and stabilising agents were optimised to recapitulate physiologically relevant oxygen levels. The 3D scaffolds, regardless of plasma or PBMC source, were subsequently cultured under both physoxia and hypoxia conditions. Specifically, scaffolds were incubated either in 1.5% O_2_ hypoxic incubator to recreate the oxygen levels in the hypoxic advanced-stage HGSC-TME with high ECM remodelling originating from the HHR model (hypoxia-high remodelling of ECM) or in a 21% O_2_ incubator recreating the physoxic oxygen levels in the site of tumour initiation or early stage of the disease characterised by physoxic oxygen levels and low ECM remodelling generating the PLR model (physoxia-low remodelling of ECM). When needed, the cells were isolated for further analysis by digesting the 3D scaffolds with 2000 U/ml collagenase I for 2 hours at 37 °C.

### Bulk RNA sequencing

Primary cells isolated from dissociated HGSC stage III tumour were cultured in PLR or HHR for 3 or 7 days. Fresh tumour on day 0, not grown in the 3D model, was used as reference. Total RNA was extracted on respective days using Qiagen RNeasy Mini Kit following the manufacturer’s instructions. Briefly, the tumour and the 3D scaffolds were lysed using RLT lysis buffer supplemented with β-mercaptoethanol and automatic tissue homogeniser. The RNA was eluted in nuclease-free water, and 400 ng were sent for sequencing with Illumina NovaSeq X Plus (Novogen). Messenger RNA was purified from total RNA using poly-T oligo-attached magnetic beads. After fragmentation, the first strand cDNA was synthesised using random hexamer primers followed by the second strand cDNA synthesis. The library was prepared after end repair, A-tailing, adapter ligation, size selection, amplification, and purification. Libraries were quantified using a Qubit fluorometer, with size distribution monitored on a representative subset of samples via the Agilent 5400 Fragment Analyzer. For Illumina sequencing, libraries were pooled based on the required data amount and effective concentration.

Raw gene-level count data were imported into DESeq2 (v1.44.0) in R software. Library size normalisation was performed via size-factor estimation, followed by variance stabilisation using the DESeq2 variance stabilising transformation (VST) with design awareness (blind = FALSE).

To analyse gene enrichment scores for each individual sample reflecting the within-sample biology, ssGSEA was performed in GSVA (v1.52.3) on the variance-stabilised expression matrix as input and gene sets defining the molecular subtypes of HGSC, namely C1 (mesenchymal), C2 (immunoreactive), C4 (differentiated) and C5 (mesenchymal-like)^32^. In addition, gene enrichment scores for hypoxia signatures, matrisome and TGF-β signalling were assessed using the publicly available HALLMARK_HYPOXIA, NABA_MATRISOME and HALLMARK_TGF_BETA-signalling gene set collections (GSEA database). Similarity among the native HGSC tissue and tumour grown in 3D model was assessed comparing the ssGSEA enrichment profiles between each sample and the native tissue using Pearson correlation. For each sample, a vector of ssGSEA scores across C1 subtype or hypoxia/TGF-β signalling/ NABA Matrisome pathways were correlated with the corresponding vector from native tissue.

### Single-cell RNA sequencing analysis

Tumour biopsy from a patient diagnosed with HGSC stage III was dissociated and primary cells were grown in co-culture with matching PBMCs in PLR or HHR models made with autologous plasma for 5 days. scRNA-seq profiling was performed on the engineered 3D models; matched primary tumour tissue from the same specimens was not available for parallel sequencing. PBMCs from the same patient, not grown in the 3D scaffold, were used as a control. The 3D scaffolds were digested, and the cells were resuspended in PBS with 0.04% (w/V) bovine serum albumin. 50 K cells with cell viability higher than 97% were collected to perform scRNA-seq library preparation.

Chromium Next GEM Single Cell 3’ Kit v3.1, 16 rxns (PN1000268, 10x Genomics) was used to prepare the libraries loading 16,500 cells/lane. Paired-end scRNA-sequencing was performed using Illumina NovaSeq6000. The scRNA-seq reads were aligned using CellRanger (10x Genomics, Cell Ranger, v6.0.1)^93^. Initially, CellRanger count was executed on each sample individually, followed by CellRanger aggregate to generate a consolidated expression matrix, a gene vector, and a barcode vector. The resulting expression matrix, along with the gene and barcode vectors, was imported into R (version 4.4.0) for downstream analysis using the Seurat package v. 5.0.2^94^. Quality was evaluated by the percentage of mitochondrial reads, gene counts, and number of reads. Low-quality cells with fewer than 200 and more than 8500 unique feature counts, mitochondrial gene content exceeding 27%, or total RNA counts exceeding, 89,500 were filtered out. The remaining high-quality cells were normalised using the SCTransform function (vst.flavour = “v2”) with cell cycle score regressed out.

Principal component analysis was performed for linear dimensional reduction, and the first 50 principal components were selected for downstream analysis. Uniform Manifold Approximation and Projection (UMAP) was used for non-linear dimensional reduction and visualisation^95^. Graph-based clustering was performed on the UMAP embeddings to identify distinct cell populations. For cluster identification and annotation, the Monocle3 package (v. 1.3.7)^96^ was employed, and the Seurat object was converted using SeuratWrappers (v. 0.3.2). Single-cell clusters were identified and annotated based on a combination of canonical genes, cluster markers using the FindMarkers function, and unbiased cell type recognition using SingleR (v. 2.6.0)^97^. Three specific marker genes were used to confirm the clustering of each cell population using epithelial markers CLDN3, EPCAM, and KRT for HGSC; CALD1, STAR and TAGLN for CAFs; CD8A, CD8B and GZMK for CD8 + T cells; CD4, LTB and SELL for CD4 + T cells; GNLY, PRF1 and NKG7 for NK cells; CD79A, IGLC3 and MS4A1 for B cells; and CD14, CD68 and LYZ for Mφ^63,98^. Dot plots were generated using the DotPlot function in Seurat to visualise the expression of selected marker genes across identified clusters, specifically classical hypoxic genes and hypoxic signature correlated with bad prognosis in ovarian cancer patients^33^, matrisome genes^99,100^, CAFs genes^99,101^, TGF-β signalling genes^102^, and activation, exhaustion and cytotoxicity genes of immune cells^63,103^.

To identify differentially expressed genes (DEGs) in HHR compared to PLR within each cell type, the FindMarkers function in Seurat was used with default parameters. DEGs were defined as genes with an absolute log2-fold change greater than 0.4 and a *p* value threshold lower than 0.05. Previously mentioned genes correlated with matrisome, TGF-β signalling, and activation, exhaustion, and cytotoxicity of immune cells were colour-coded and highlighted in orange, blue, green, purple, and red, respectively in the volcano plots and DEGs tables.

CellChat R package (v.2.1.2)^104^ was used to quantitatively infer intercellular communication networks from scRNA-seq data. Circle plots show putative ligand–receptor interactions between cells, where the thickness of the line represents the strength of the communication. Heatmaps represent the summary of the signalling pathways that contribute to outgoing or incoming communication where the colour bar represents the relative signalling strength of a signalling pathway across cell types, and the bars indicate the sum of the signalling strength of each cell type or pathway. Bubble plots show the probability of interactions between receptors and ligands among different cell types with senders on the y-axis and receivers on the *x* axis. Weighted-directed network in CellChat was used to identify the signalling roles of the cell types represented by a heatmap that shows the senders (cell type sending signals), receivers (cell type receiving signals), mediators (cell type that controls the communication flow between the cells), and influencers (cell type that influences the information flow between cells) of intercellular communication.

### Characterisation of physiologically relevant oxygen levels by optical sensor

KURAMOCHI, OVSAHO, OVCAR-3, OVCAR-8, CAOV3, OV81.2 and HEYA8 cells were multi-cultured with CAFs and PBMCs in PLR and HHR. Alternatively, dissociated HGSC tumours were grown in both 3D models. The partial pressure of oxygen (pO_2,_ kPa) was measured in the middle of the 3D scaffolds on days 0, 2, 3, and/or 7, using the optical sensor PreSens Needle-Type Oxygen Microsensor NTH-PSt7 and manual micromanipulator following the manufacturer’s instructions.

### Immune infiltration assay

For immune infiltration studies, KURAMOCHI cells were monocultured or co-cultured with CAFs in PLR or HHR for 3 days to allow ECM remodelling and study its influence in immune infiltration. Alternatively, the 3D scaffolds were pre-treated with galunisertib (LY2157299, 10 μM), a targeting drug for TGF-β signalling, to prevent collagen I expression and ECM remodelling. After three days, PBMCs were added on top of the 3D scaffold and incubated for 4 days to allow immune infiltration inside the 3D scaffolds. Additionally, dissociated tumour tissue from HGSC patients was co-cultured with CAFs in the 3D model engineered with autologous HGSC plasma and the infiltration studies were performed with matching PBMCs in the same settings to recreate a precision-based preclinical model.

### Decellularization and re-cellularization of human ovaries

Whole human ovaries were decellularized by submersion in 0.1% (w/V) sodium dodecyl sulphate (SDS) in sterile PBS 1× solution with continuous rotation and daily replacement of the solution for 3 days. Ovaries were transferred to 0.05% (V/V) Triton X-100 solution in sterile PBS 1× for 3 hours, followed by several washes with sterile water over 24 hours. The tissue before and after decellularization was characterised by IHC. The dECM was frozen in optimal cutting temperature and cryosectioned in 50-μm-thick sections, which were placed into eight-well confocal trays. For re-cellularization, the dECM was submerged in DMEM/HAM’s F-12 media for 3 hours. Then, the media was removed and KURAMOCHI cells and CAFs were added on top of dECM in 20 µl of media and incubated for 5 hours to allow attachment of the cells to the matrix. More media was added on top and cultured for 3 days in dPLR (decellularized physoxia-low remodelling of ECM) model or in dHHR (decellularized hypoxia-high remodelling of ECM) model in the presence or absence of galunisertib (10 μM). On day 3, PBMCs were added on top of dECM to penetrate for 4 days and immune infiltration was analysed by confocal microscopy.

### Immunohistochemistry and IF

KURAMOCHI cells, CAFs, PBMCs or their co-cultures were seeded inside of the 3D scaffolds in BEEM® Embedding Capsules (1001, Spi Supplies). For re-cellularized dECM assessment, KURAMOCHI cells and CAFs were co-cultured on top of the dECM for 3 days. The 3D scaffolds and dECM were fixed in 10% neutral buffered formalin, paraffin embedded and processed on a Leica 300 ASP tissue processor. The 3D models were oriented with the top facing forward to identify the top and the bottom of the model, and the blocks were serially sectioned at 10 µm. The paraffin-embedded HGSC tissues were processed in similar way. The sections were stained with the Discovery Ultra automated slide staining system with specific antibodies for immunohistochemistry (IHC) or IF (Supplementary Table 2). The antigen retrieval step was performed using the cell conditioning CC1 solution (basic pH Tris based buffer). Omission of the primary antibody served as a negative control.

For IHC, the PLR and HHR scaffolds were stained for HIF-1α (master transcription regulator in response to hypoxia), extracellular and intracellular collagen I, or α-smooth muscle actin antibody (α-SMA) as a marker for CAF activation. The native ovarian tissues and dECM were stained for collagen I and fibronectin and the re-cellularized dECM for collagen I. HGSC tumour tissues were stained for CD8 + T cells, collagen I and TGF-β. The OmniMap HRP detection kit was used with DAB as the chromogen and the slides were counterstained with hematoxylin. Moreover, native ovarian tissues and dECM were stained with Masson’s trichrome, picrosirius red and hematoxylin and eosin (H&E) following standard protocols. The slides with picrosirius red were imaged by confocal microscopy and the rest in brightfield on Aperio VERSA Scanning system with ×20 magnification and analysed with Aperio ImageScope x64 software. Signal intensity and positivity (area of positive staining divided by total tissue area) were measured using the positive pixel count v9 algorithm in at least five randomly picked ROI per section in cell lines or in two ROIs with high or low CD8 T cells abundance in HGSC patients. The positivity for stromal staining (blue) in Masson’s trichrome staining was determined in ImageJ (FIJI) applying first applying the colour deconvolution plugin and the same threshold for all ROIs.

For spatial patterns of immune infiltration, HGSC tissue sections were stained by multiplex IF staining in sequential cycles with target specific primary antibody, then HRP conjugated anti-species secondary antibody and then fluorophores. Epithelial, stromal and immune cells were detected with anti-cytokeratin (magenta), anti-vimentin (red) and anti-CD45+ (green) antibodies (Supplementary Table 2), respectively. The primary-secondary antibodies complex was removed by heat denaturation using BenchMark CC2 buffer. Finally, the slides were counterstained with Hoechst for nuclei detection.

### Fluorescent imaging

Fluorescent images of HGSC tissue sections stained with picrosirius red and multiplex IF were acquired using Nikon NiE microscope with ×10 or ×40 magnification, respectively, detecting at least two ROIs per section. 3D models and dECM were imaged on Nikon A1R Ti2E confocal microscope with ×10 magnification, 1024 × 1024 resolution and performing z-stacks with 0.4-μm step size. The images were processed with NIS-Elements AR Analysis 5.21.03 Software. Hypoxic status of the cells grown in PLR or HHR was detected by using Image-iT^TM^ Green Hypoxia Reagent (5 μM), which fluoresces green in environments with oxygen levels lower than 5%. KURAMOCHI, CAFs, or PBMCs in monoculture or their combinations in multi-culture KURAMOCHI:CAFs, or KURAMOCHI:CAFs:PBMCs or, alternatively, dissociated HGSC tumours were cultured in PLR or HHR for 7 days, and the hypoxia-sensitive dye was added 24 h prior to imaging. To explore cell viability, the cells were stained with NUCLEAR-ID® blue/red cell viability reagent following the manufacturer’s instructions. This reagent stains the live cells in blue (permeable nucleic acid stain) and dead cells in red (impermeable nucleic acid stain). For visualisation purposes, the blue colour of live cells was modified to green colour. The number of live and dead cells was manually counted with the Cell Counter Plugin in ImageJ and the cell death was expressed as the percentage of dead (red) cells out of the total number of cells. For immune infiltration studies in PLR and HHR, the cells were stained 24 hours prior to imaging with antibodies to detect infiltrated PBMCs (CD45 + , green) in either cell lines KURAMOCHI (MUC1 + , magenta) and CAFs (CD90 + , red) or in dissociated tumours stained with cytokeratin+ (magenta) for epithelial cells and FAP+ (red) for primary CAFs (Supplementary Table 2). For immune infiltration studies in decellularized matrix from human ovaries, the cells were pre-stained with lipophilic cell surface dyes, KURAMOCHI (DiD + , magenta), CAFs (DiI + , red), and PBMCs (DiO + , green). Unless otherwise stated, the z-stack image of the 3D view was converted to a 2D maximum projection, which takes all the 3D data and turns it into a single 2D image for better visualisation and quantification purposes. To count the number of infiltrated PBMCs (green), automatic binary thresholding was used to detect the cells in the green channel with area restriction lower than 50% of the maximum area detected for all objects (0–25 μm^2^) to avoid the counting of nonspecific stains. OrientationJ for Hue, Saturation and Brightness (HSB) colour-survey maps and OrientationJ distribution measurement plugins in ImageJ (FIJI) were used to determine the orientation and alignment of the collagen fibres in picrosirius red images of dECM.

### Second harmonic generation microscopy

KURAMOCHI cells or CAFs were monocultured or co-cultured in PLR or HHR for 7 days. Second harmonic generation microscopy (SHG) was performed to detect collagen fibres with FLUOVIEW FVMPE-RS Multi Photon Laser Scanning Microscope (Olympus) with a tunable laser (InSight, SpectraPhysics) at 840 nm. The samples were imaged using ×25 water-immersion objective in two different positions of the 3D model and acquiring z-stacks of 50 μm with a step size of 1 μm. Scan size was set to 800 × 800 px with line averaging of 2. Six iterations of constrained iterative deconvolution were uniformly applied to the images in CellSens analysis software (Olympus). OrientationJ for Hue, Saturation and Brightness (HSB) colour-survey maps and OrientationJ distribution measurement plugins in ImageJ (FIJI) were used to determine the orientation of the collagen fibres.

### Atomic force microscopy mechanical characterisation

KURAMOCHI cells were grown in co-culture with CAFs in PLR or HHR models for 7 days. A BioScope Resolve atomic force microscopy (Bruker) in contact mode and DNP cantilever D (Bruker, nominal spring constant: 0.06 N/m) was used to collect force-indentation curves. The actual spring contact of each cantilever was measured on a flat, hard surface using the thermal noise method. On each sample, a force-volume mode was used to collect 4 × 4 force-indentation curves over areas of 2 × 2 µm^2^. All samples were measured in PBS buffer to minimise capillary force in air. All force curves were recorded by applying a loading force of 3 nN, with a constant retraction speed of 1 µm/s, a ramp size of 5 µm, and 0.1 s of surface delay. The force-indentation curves were analysed using the NanoScope analysis software (v1.9, Bruker). The retract part of the curve was fitted with the Hertz model:

$$F=\frac{4}{3}\frac{E}{(1-{v}^{2})}{R}^{\frac{1}{2}}\cdot {\delta }^{\frac{3}{2}}$$

where E is the Young’s modulus, *δ* is the indentation depth, *v* is the Poisson ratio (0.3 was used here), and *R* is the radius of the tip curvature (20 nm).

### Imaging flow cytometry

Imaging flow cytometry was applied to quantitatively determine the intracellular TGF-β and collagen I expression. KURAMOCHI, CAFs or their co-culture were maintained for 7 days in PLR and HHR in the presence or absence of galunisertib (10 μM), to further explore its mechanistic properties and influence on ECM remodelling through collagen synthesis. On day 7, the 3D scaffolds were digested, and the cells were isolated, fixed and permeabilized using the Cyto-Fast^TM^ Fix/Perm Buffer Kit, and stained with antibodies (Supplementary Table 2) to measure intracellular TGF-β or collagen I (green) and to distinguish CAFs (FAP + , red) from KURAMOCHI cells (FAP-) in the co-culture. Samples were analysed at low speed and ×40 magnification with the Cytek® Amnis® ImageStream®X MkII Imaging Flow Cytometer and INSPIRE software. Images were acquired with a 488 nm laser power of 20 mW (TGF-β assay) or 50 mW (collagen I assay) to detect TGF-β or collagen I, a 642 nm laser at 125 mW (collagen I assay) or 150 mW (TGF-β assay) to detect FAP, and a 785 nm laser at 0.25 mW and in brightfield. The images were analysed using IDEAS v6.2 analysis software. Each sample was processed by first using the area of the brightfield and the brightfield aspect ratio to identify single round cells, discarding debris and speed beads. Then, the brightfield gradient RMS value was used to gate in-focus events for further analysis, and the area versus FAP intensity was used to distinguish CAFs from KURAMOCHI cells. Finally, events with no Mean Pixel FITC intensity were excluded from the analysis, discarding debris. The area of each cell was calculated using a brightfield-based object mask, and the intensity of collagen I or TGF-β was calculated from the pixel intensity within the cell mask. Finally, the mean fluorescence intensity (MFI) was analysed in individual cells in FlowJo_v10.8.1_CL software and representative histograms were generated for each condition (Supplementary Fig. 9c).

### Western blot

KURAMOCHI cells, CAFs, or their co-culture were grown in the PLR or HHR for 7 days in the presence or absence of galunisertib to analyse its influence on TGF-β downstream signalling. To explore the role of oxygen levels and different cell types in the expression of metalloproteases, PBMCs, KURAMOCHI, CAFs, KURAMOCHI:CAFs and KURAMOCHI:CAFs:PBMCs were grown for 7 days in the PLR and HHR. On day 7, the above mentioned conditions were lysed with RIPA Buffer with addition of phenylmethulsulfonyl fluoride 1 mM, dithiotreitol 0.5 mM, phosphatase inhibitor cocktail 2 and phosphatase inhibitor cocktail 3. The samples were sonicated on ice and centrifuged at 16,000 × *g* for 15 min at 4^o^C and the concentration of proteins was determined by Pierce 660 nm Protein Assay Kit following the manufacturer’s instructions. Gel electrophoresis was used in 10 or 15% Mini-Protean TGX Precast Polyacrylamide gels and transferred to PVDF membranes using the Trans-Blot Turbo RTA Mini 0.2 µm PVDF Transfer Kit. The membranes were blocked in Pierce Clear Milk Blocking Buffer and then incubated with antibodies (Supplementary Table 2) for metalloproteinase-9 (MMP-9), canonical (TGF-β, SMAD 2/3, phospho(p)-SMAD2/SMAD3, SMAD4) and non-canonical (Akt/pAkt, MAPK/pMAPK) TGF-β signalling. β-actin was used as a loading control. Anti-rabbit IgG, HRP-linked secondary antibody and Immobilon® Western Chemiluminescent HRP Substrate were used to detect specific bands on the ChemiDoc MP Imaging System and the images were processed in Image Lab Software.

### Flow cytometry

The 3D scaffolds were digested on the day of analysis, and the isolated cells, unless otherwise stated, were stained with LIVE/DEAD^TM^ Fixable blue dead cell stain kit, fixed and permeabilized with the Cyto-Fast^TM^ Fix/Perm Buffer Kit to detect intracellular markers, blocked with the Human TruStain FcX^TM^ and stained with specific antibodies (Supplementary Table 2). To detect HIF-1α expression and intracellular cytokines or activation and exhaustion surface markers in patient-derived cultures, primary HGSC cells were co-cultured with matching PBMCs for 2 days inside the PLR or HHR generated with autologous plasma. The PBMCs were pre-stimulated for 5 hours for immune functional characterisation. In the immunophenotyping studies, unstained cells were stained to detect cancer cells (B7H3 + ), CAFs (FAP + ), CD3 + , CD4 + , CD8 + T lymphocytes, macrophages (CD14 + ), B cells (CD19 + ), NK cells (CD56 + ), and myeloid-derived suppressor cells (MDSC, CD11b + ). To explore the cytotoxic effect of PBMCs on HGSC, KURAMOCHI cells were mono-cultured or co-cultured inside the PLR and HHR with stimulated PBMCs for 5 days and unstained cells were stained with antibodies to detect PBMCs (CD45 + ) and KURAMOCHI (CD45−) and then with propidium iodide (PI) and Annexin V kit to detect live, early apoptotic and necrotic cells. Alternatively, the cytotoxic effect of the immune cells on HGSC was evaluated in 2D cultures of KURAMOCHI cells stained with cell surface marker DiO (green) co-cultured with PBMCs at increasing ratios (1:1, 1:2, 1:3 and 1:4) in normoxia (21% O_2_) or hypoxia (1.5% O_2_) for 2 days. Flow cytometry was performed using the BD FACS Fortessa and FACSDiva v6.1.2 software by acquiring a minimum of ten thousand Precision Count Beads^TM^. The data were analysed using FlowJo v10 software. HIF-1α expression was analysed by manual gating, and cell counts were normalised to counting beads. For immune functional assays and immunophenotyping studies, single live cells were analysed by dimensionality reduction analysis using Phenograph v2.5.0 and UMAP v4.1.1. (default settings) Plugins in the FlowJo software. The level of expression of the markers in functional assays was determined as the frequency (percentage of the CD4+ or CD8+ parent population), and the stacked charts expressed the percentage of each subpopulation in the total population of CD4+ or CD8 + T cells, with a total of 100%. The different cluster populations of infiltrated lymphocytes were identified with different colours and numbers and expressed as counts of cells positive for the selected marker. inside the 3D scaffold. The cytotoxic effect of PBMCs on KURAMOCHI cells in the 3D scaffolds was expressed as a percentage of KURAMOCHI cells in each stage (live, early apoptosis and necrosis), and the cytotoxic effect in 2D was determined as the fold change of live KURAMOCHI cells in the absence of PBMCs. Gating strategies are included in Supplementary Fig. 13.

### Luminex immunoassays

Customised ProcartaPlex (PPX45MXGZF4J, Thermo Scientific) was used following the manufacturer’s instructions to detect extracellular pro-tumoural and anti-tumoural cytokines and growth factors (Supplementary Table 2) from supernatants of digested 3D scaffolds and culture media mixed in a 1:1 ratio and collected from infiltration assays in the patient-derived model. Customised Human Luminex®Discovery Assay (R&D Systems) (Supplementary Table 2) was used to detect chemokines in supernatants of digested 3D scaffolds and culture media mixed in a 1:1 ratio and collected from KURAMOCHI, CAFs and their co-culture. The samples were analysed with the Luminex 200 analyzer and Belysa® Immunoassay Curve Fitting Software using 4-PL Sigmoidal curve extrapolation to detect the concentration (pg/ml) of each analyte based on standards. For patient-derived models, the data were standardised by calculating the z-score, which is accomplished by subtracting the mean of all samples divided by the standard deviation of all samples for each analyte. The heatmaps were generated using the median of the z-score of six patients per condition.

### ELISA

The cultures of PBMCs, KURAMOCHI, CAFs, KURAMOCHI:CAFs or KURAMOCHI:CAFS:PBMCs were grown for 7 days in the PLR or HHR. ECM metalloproteinase 9 (MMP-9) was detected in the supernatants from the digested 3D scaffolds and the culture media (mixed in 1:1) using the Human MMP-9 ELISA Kit-Quantikin following the manufacturer’s instructions. 4-PL Sigmoidal curve extrapolation was used to calculate the concentration (ng/ml) of MMP-9 based on standards.

### Statistical analysis

In cell lines, experiments were performed in triplicates and repeated at least three times, unless otherwise noted. Data are represented as superplots^105^ to capture cell variability and experimental reproducibility, with biological replicates shown as larger, darker symbols and technical replicates as smaller, lighter symbols. All graphs were generated and analysed using GraphPad Prism 9 software. The statistical analysis was performed on the Mean ± SD of biological replicates using a paired *t* test, unpaired *t* test or ANOVA multi-comparisons Tukey test, except for imaging flow cytometry data, where the median was represented with 95% confidence interval (CI) with ANOVA.

Primary cells (dissociated tumours or PBMCs) included a sample size of at least six individuals per experimental group, unless otherwise stated. Paired-t test was used for statistical analysis in immune infiltration assays comparing PPR vs HHR. Linear mixed-effects model was used to assess the statistical significance of the treatment with galunisertib compared to control in i) PLR, ii) HHR and iii) the model x treatment interaction (difference-in-differences, ratio of ratios, ROR). In the confocal microscopy dataset (multiple ROIs per donor; *n* = 3 donors), replicate fields within each model and treatment condition were aggregated on the log scale using a 10% trimmed mean of log(1 + abundance) to reduce the influence of outliers and analysed using a replicate-aware, donor-paired 2 × 2 factorial framework. Replicate reliability was assessed using intraclass correlation coefficients (ICCs) from random-intercept models. For flow cytometry (one measurement per patient), log-transformed abundances [log(1 + abundance)] were analysed using a 2 × 2 factorial linear mixed-effects model with donor as a random intercept. Multiple testing was controlled using the Benjamini–Hochberg false discovery rate (FDR) procedure. Effect sizes were reported on the natural-log scale and as back-transformed ratios and log₂ fold changes with 95% confidence intervals and represented on paired donor-level dot plots and forest plots of log_2_ fold changes (Gal/control) in PLR or HHR and log_2_ ROR (HHR ÷ PLR) for interaction model x treatment. In all studies *p* value lower than 0.05 or *q* value lower than 0.10 were considered significant.

### Reporting summary

Further information on research design is available in the Nature Portfolio Reporting Summary linked to this article.

## Supplementary information

Supplementary Information

Description of Additional Supplementary Files

Supplementary Data 1

Reporting Summary

Transparent Peer Review file

## Source data

Source Data

Source Data Western Blots

## Data Availability

The bulk RNA sequencing data are submitted to the GEO database (GSE327288, https://www.ncbi.nlm.nih.gov/geo/query/acc.cgi?acc=GSE327288). The single-cell RNA sequencing data are submitted to the GEO database (GSE299200, https://www.ncbi.nlm.nih.gov/geo/query/acc.cgi?acc=GSE299200). All relevant data supporting the findings of this study are available within the paper and its Supplementary Information. Source data for the figures are provided with this paper. All relevant raw files have been deposited in Zenodo under DOI 10.5281/zenodo.20514905. Source data are provided with this paper.

## Code availability

This study utilised the open-source R packages Seurat (https://github.com/satijalab/seurat) and CellChat (https://github.com/jinworks/CellChat) for quality control, analysis, and visualisation of single-cell RNA-seq data and cell-cell communication. For bulk RNA-seq analysis ssGSEA (https://rpubs.com/snijesh/ssgsea-analysis-explained) and GSVA analysis were performed in R software.
