# Supplementary material for: Patient-derived model capturing hypoxia and extracellular matrix remodelling of immunologically cold high-grade serous tumours
Source: Nat Commun. 2026 Jul 11;17:8568. doi: 10.1038/s41467-026-75262-8 (PMC13482822; doi:10.1038/s41467-026-75262-8)
Supplement: Supplementary file 1 [file 41467_2026_75262_MOESM1_ESM.pdf]

Supplementary Materials:

Patient-derived model capturing hypoxia and extracellular matrix remodelling of immunologically cold high-grade serous tumours

Simona Plesselova[1], Hailey Axemaker[1], Kristin Calar[1], Oduduabasi Isaiah[2], Jared Wollman[3], Somshuvra Bhattacharya[1], Etienne Z. Gnimpieba[4], Darci M. Fink[5], Congzhou Wang[6], Hiruni Sumanasiri[7], Kristina W. Thiel[7,8], Maria Bell[9,10], Pilar de la Puente[1,3,10, 11 *]

* Corresponding author: pilar.puente@sanfordhealth.org)

Containing:

Supplementary Fig 1-13

Supplementary Table 1-2

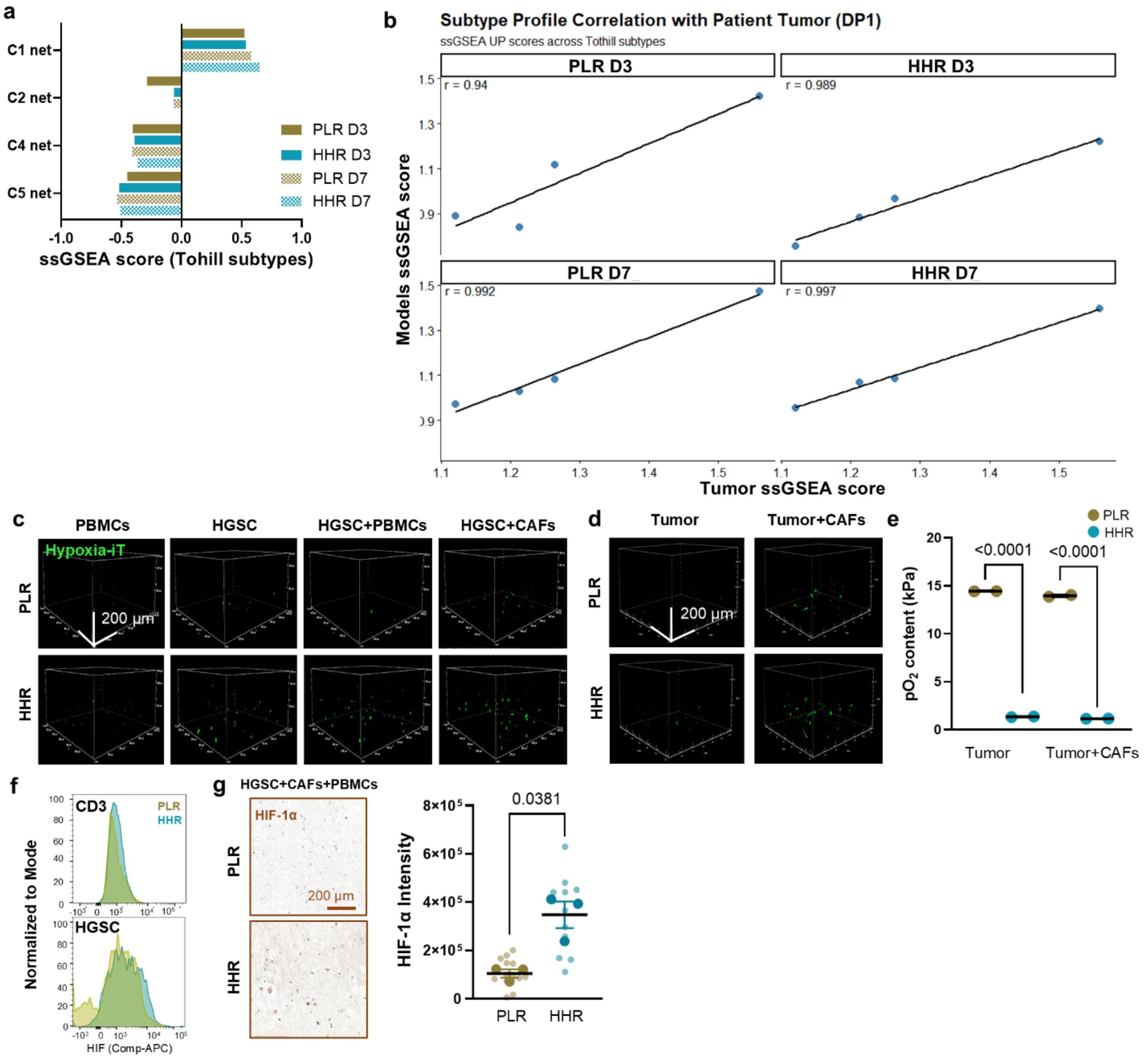

Supplementary Fig. 1. PLR and HHR models conserve the molecular fingerprints of HGSC tumours and recapitulate the physiologically relevant oxygen levels in early vs advanced HGSC. a Single-sample Gene Set Enrichment analysis (ssGSEA) of net scores for molecular subtypes of HGSC defined by Tothill et. al (2008): C1 (mesenchymal), C2 (immunoreactive), C4 (differentiated) and C5 (mesenchymal-like) in HGSC fresh tumours and the tumours grown in PLR or HHR for 3 days (PLR&HHR D3) or 7 days (PLR&HHR D7) detected by bulk RNA-seq. **b** Pearson correlation of gene scores for upregulated gene set across Tothill subtypes in 3D models (PLR and HHR, D3 and D7) correlated with fresh tumours. **c** Representative confocal microscopy images in 3D view of hypoxic cells (green) in monoculture of PBMCs, and HGSC (KURAMOCHI) or in co-cultures of HGSC+PBMCs, HGSC+CAFs or **d** dissociated HGSC tumours in the presence or absence of CAFs in PLR and HHR. Scale=200µm**.** Same results were identified for 3 independent models. **e** Oxygen levels measured at day 7 by an optical sensor in HGSC tumours in monoculture or co-culture with CAFs inside PLR and HHR. Mean±SD, ANOVA two-tailed. n=2 patient samples. **f** HIF-1α detected by flow cytometry in primary HGSC tissue or PBMCs in monocultures, n=2 patient samples and **g** by IHC in multi-culture of KURAMOCHI, CAFs and PBMCs. Scale= 200µm**.** Mean±SD. Welch's t-test two-tailed. Data are represented as superplots with biological replicates shown as larger, darker symbols (n=3 PLR/HHR cultures from different days) and technical replicates as smaller, lighter symbols (n=4). Source data are provided as a Source Data file.

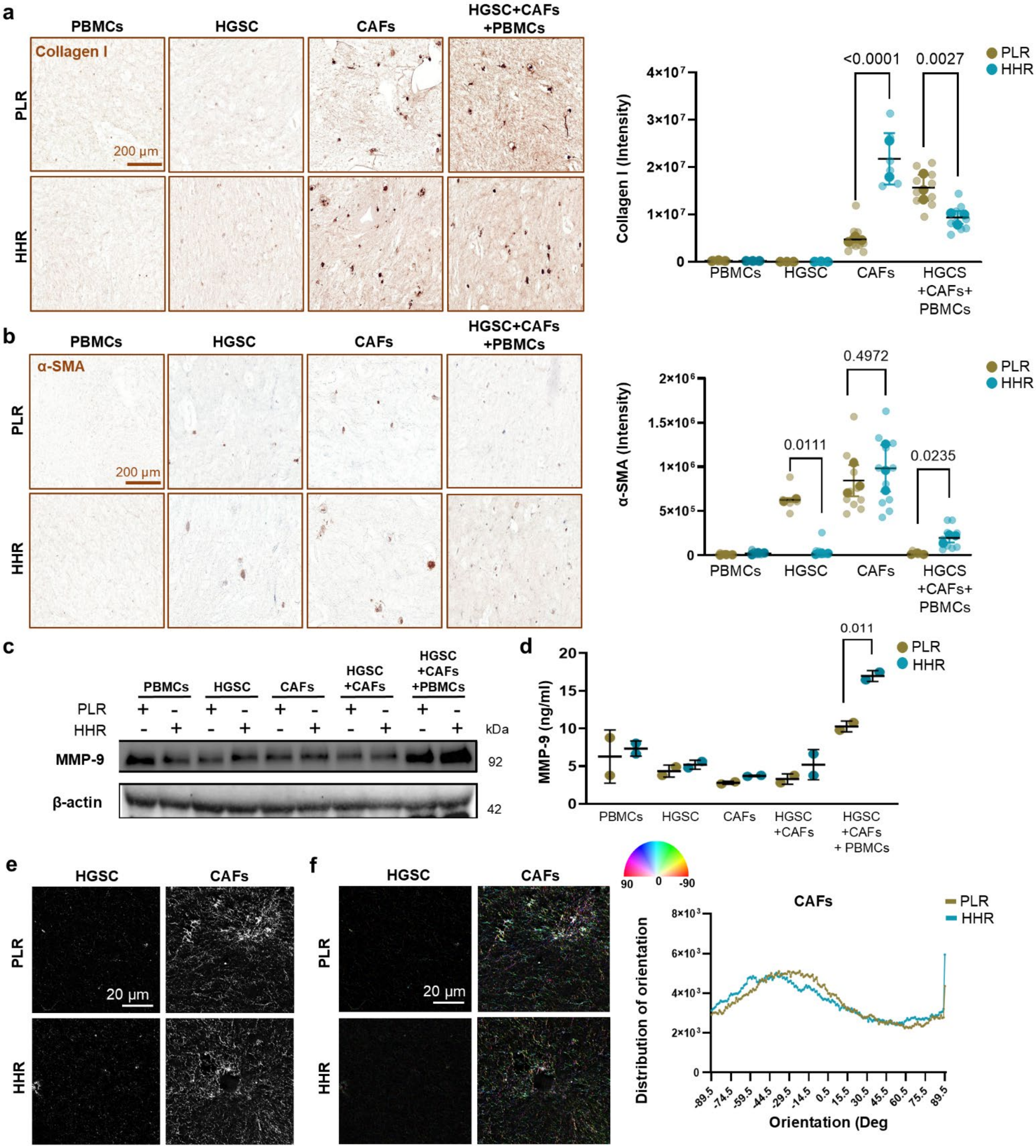

Supplementary Fig. 2. HHR mimics hypoxia-induced CAF-driven ECM remodelling. a-b Representative IHC images and quantification of collagen I and α-SMA intensity in monoculture of PBMCs, HGSC (KURAMOCHI), CAFs or their multi-culture. **c** MMP-9 detected by western blot or **d** ELISA n=2. **e** SHG microscopy images in monocultures and **f** the quantification of the distribution of orientation of collagen fibers. Same results were identified for 3 independent models. a,b,d Mean±SD, ANOVA two-tailed. a, b, Data are represented as superplots with biological replicates shown as larger, darker symbols (n=3 PLR/HHR cultures from different days) and technical replicates as smaller, lighter symbols (n=3). Source data are provided as a Source Data file.

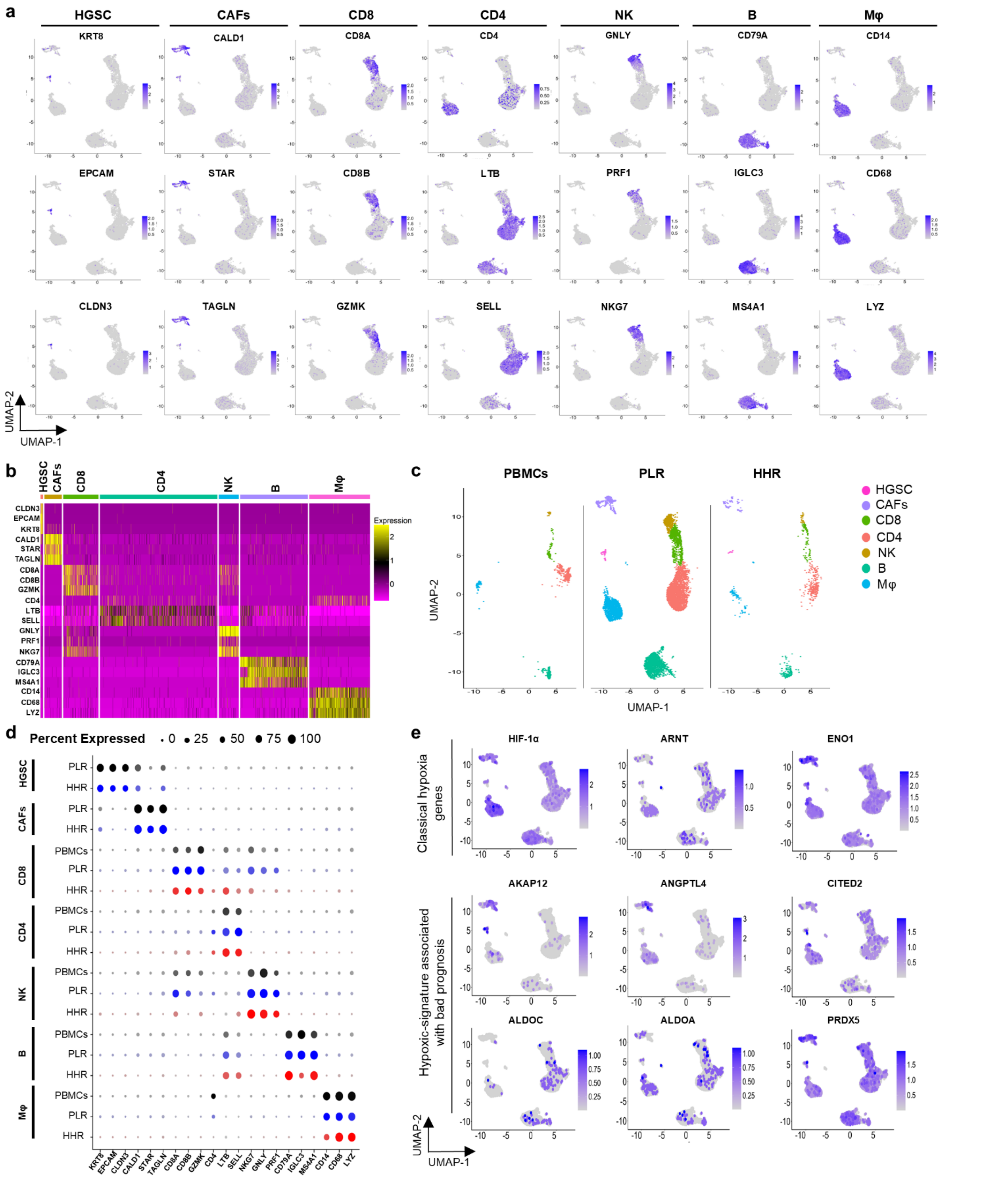

Supplementary Fig. 3. scRNA-seq analysis of PLR vs HHR. a Feature plots and **b** heatmap of marker genes in clustered cell populations. **c** Clusters of cell populations split by conditions. **d** Expression of marker genes in each cell population split by conditions. **e** Feature plots of hypoxia-related genes.

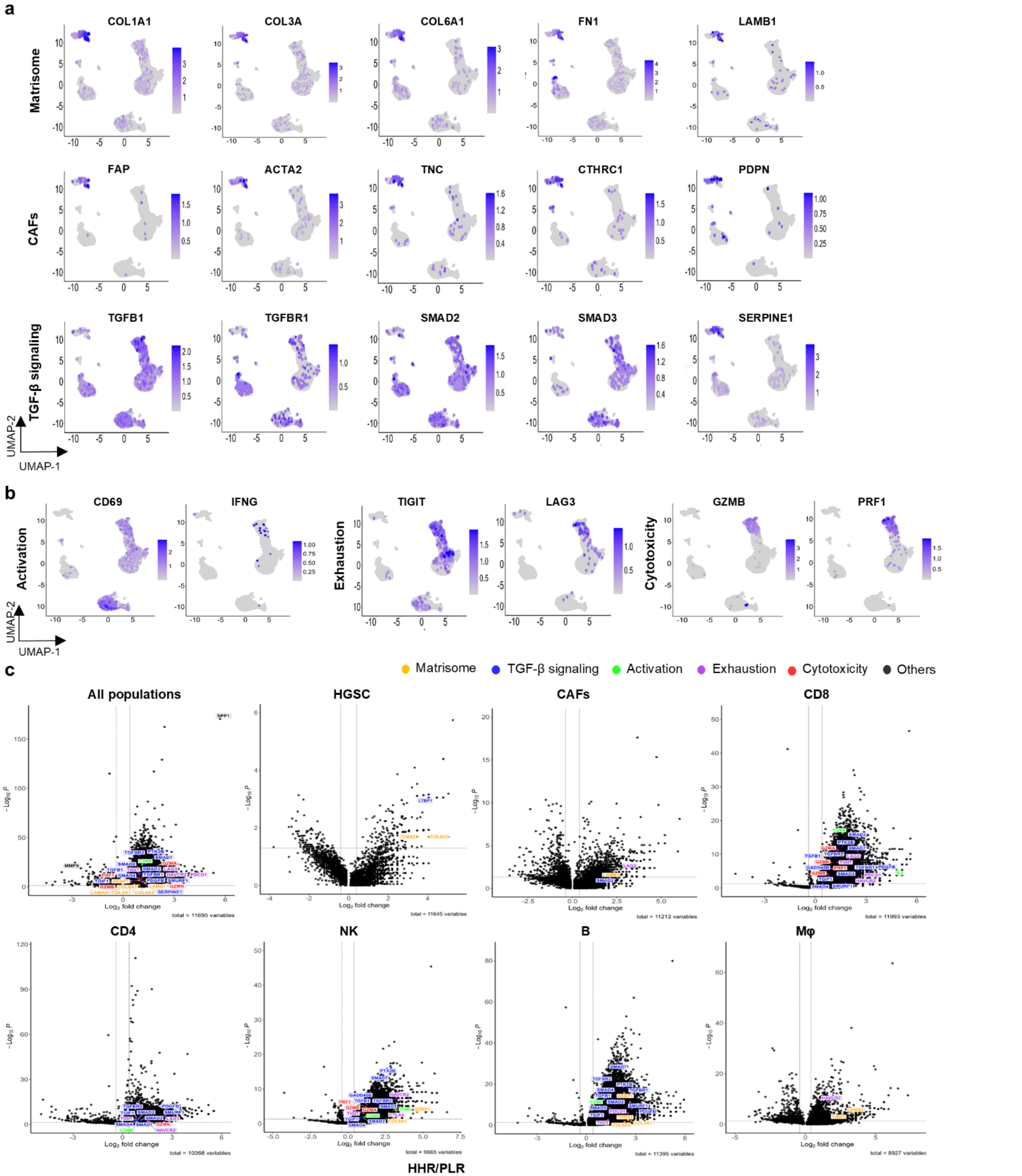

Supplementary Fig. 4. HHR reveals a transcriptional signature with involvement of ECM remodelling and immune functionality. a Feature plots showing the expression of matrisome, CAF-associated, and TGF-β signaling genes, and **b** immune activation, exhaustion, and cytotoxicity genes as identified by scRNA-seq analysis. **c** Volcano plots showing DEGs in HHR compared to PLR.

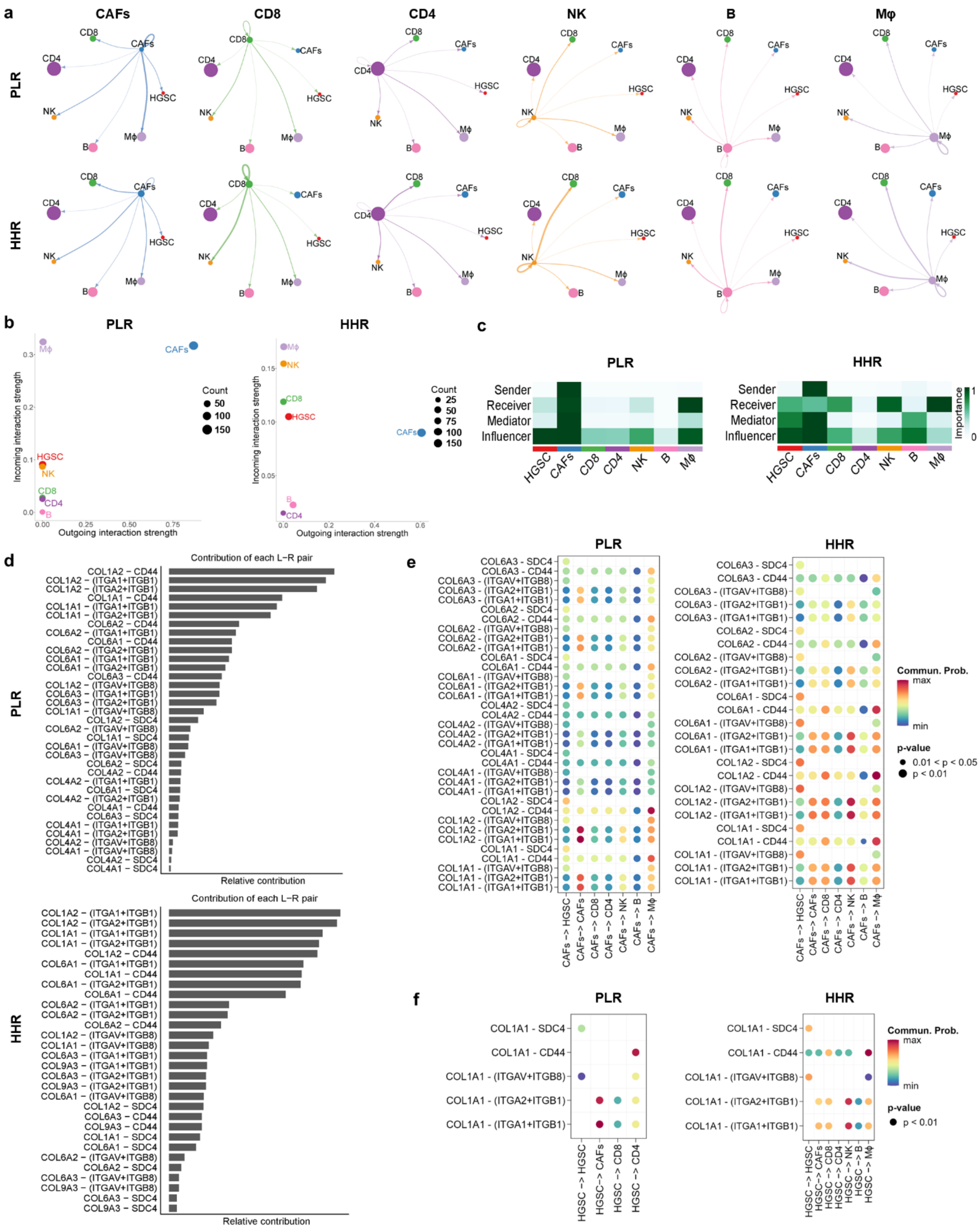

Supplementary Fig. 5. CAFs are the main contributors to the collagen signaling pathway involved in tumours-stroma-immune interactions. **a** Circle plots showing the interactions and strengths among different cell types. **b** Scatter plots identifying dominant sender and receiver cells. **c** Heatmap showing the relative importance of each cell type as sender, receiver, mediator and influencer, based on the computed four network centrality measures. **d** Relative contribution of each ligand-receptor (L-R) pair to the overall collagen signaling network. **e** Bubble plot showing comparison of significant ligand-receptor interactions from CAFs, and **f** HGSC to other cell populations.

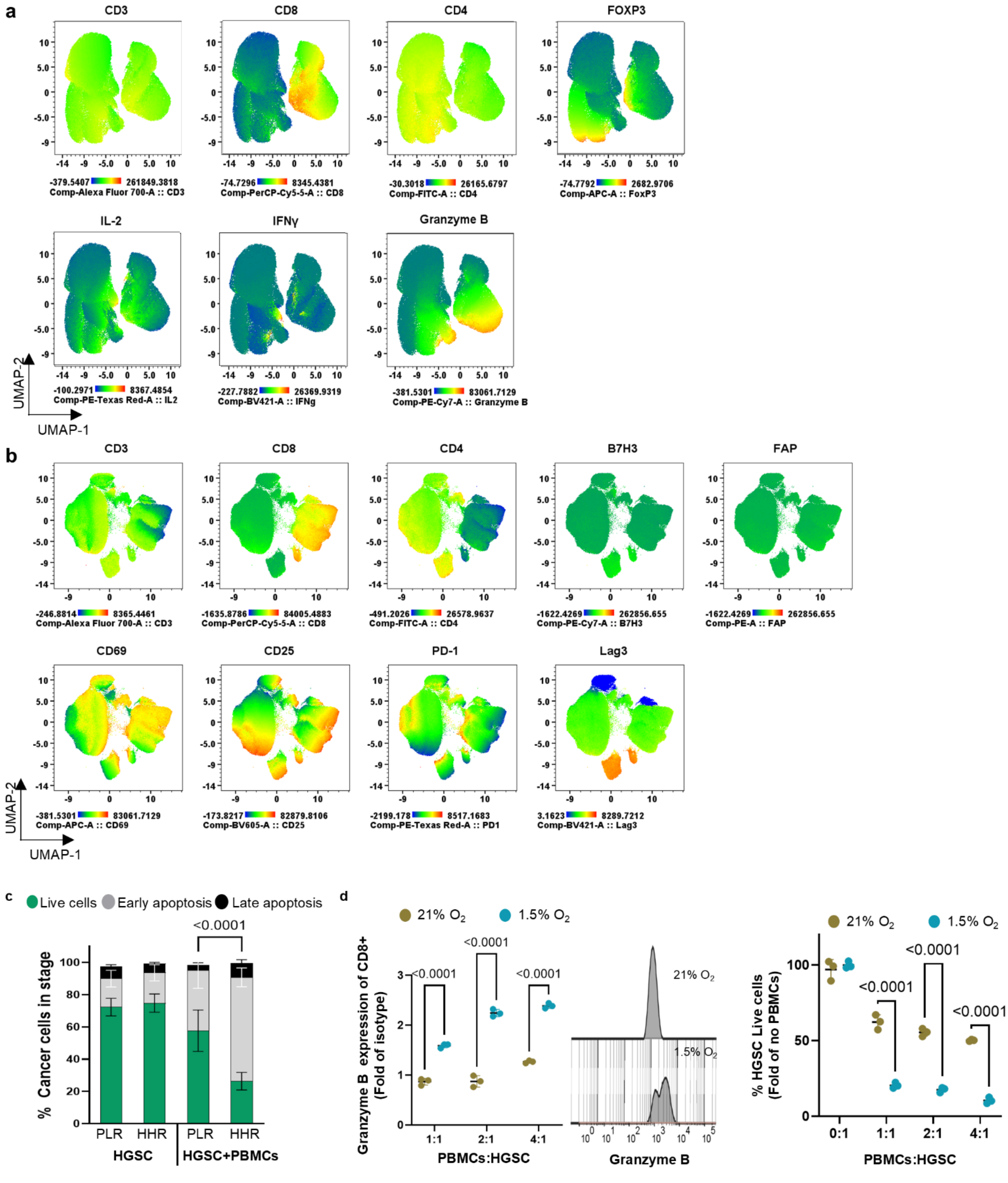

Supplementary Fig. 6. Intratumoural hypoxia induces higher functionality and cytotoxicity of PBMCs. a-b Heatmaps of functionality markers expression. **c** Apoptosis evaluation of KURAMOCHI in the presence or absence of PBMCs in HHR compared to PLRs. **d** Cytotoxicity evaluation and granzyme B expression in PBMCs cocultured with KURAMOCHI in 2D by flow cytometry. n=3 independent models. Two-way ANOVA. Source data are provided as a Source Data file.

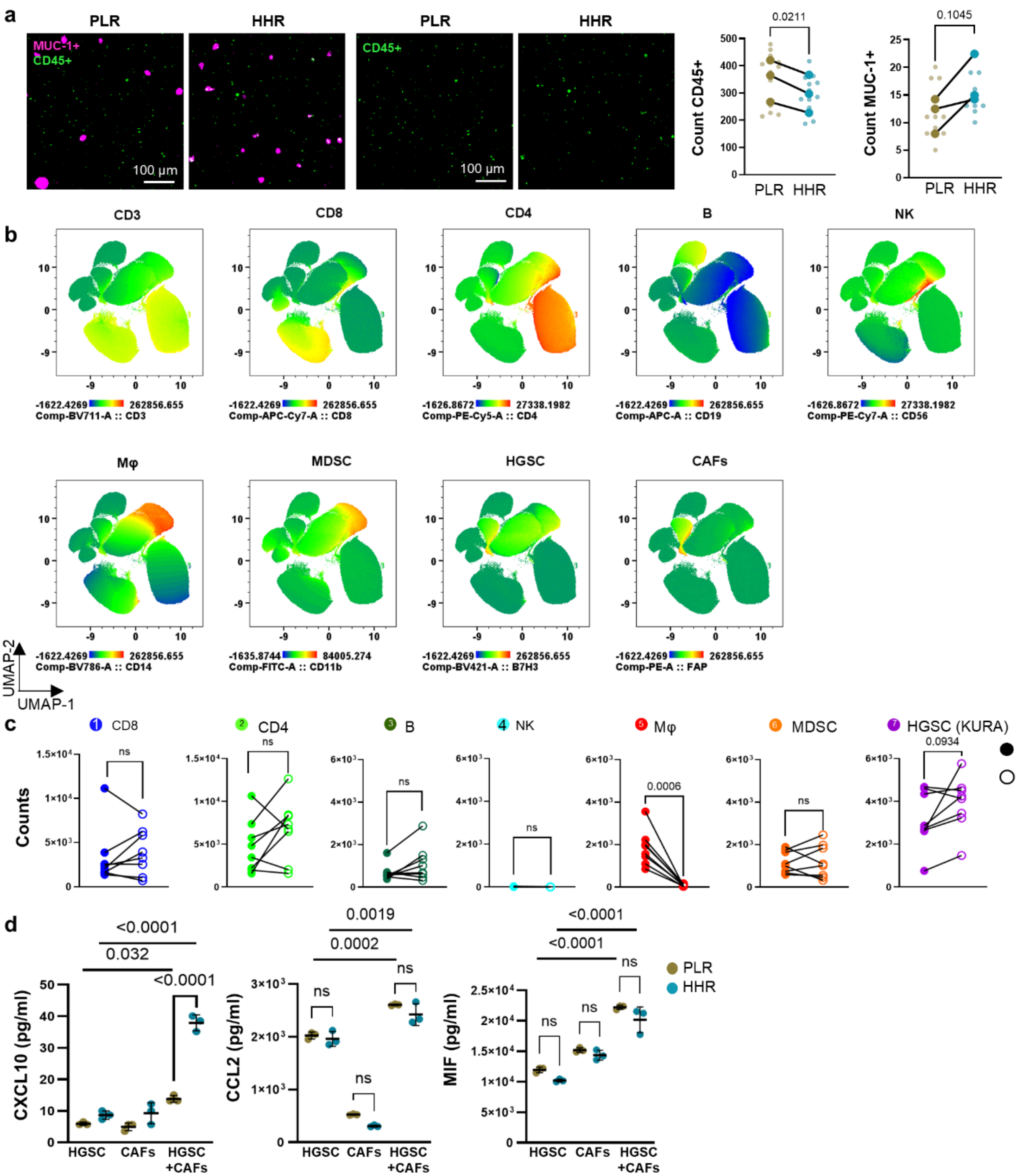

Supplementary Fig. 7. Intratumoural hypoxia induces impaired immune infiltration. a Representative confocal images in maximum projection of infiltrated CD45+ T cells (green) in 3D models with KURAMOCHI (MUC1+, magenta) and their quantifications (counts). Scale=100µm. Data are represented as donor-paired superplots with biological replicates shown as larger, darker symbols (n=3 PLR/HHR cultures from different PBMC donors) and technical replicates as smaller, lighter symbols (n=4). **b** Heatmap showing the expression of surface markers used to determine the cell populations in dimensionality reduction analysis of immune cell infiltration. **c** Donor-paired graphs showing the counts of immune cells infiltrated into 3D models with KURAMOCHI in monoculture. n=8 PBMCs from different donors. **d** Quantification of secreted chemokines from KURAMOCHI, CAFs and their co-culture in PLR or HHR on day 7. Mean±SD, n=3. a&c Paired t-test two-tailed, d one-way ANOVA , ns=not significant. Source data are provided as a Source Data file.

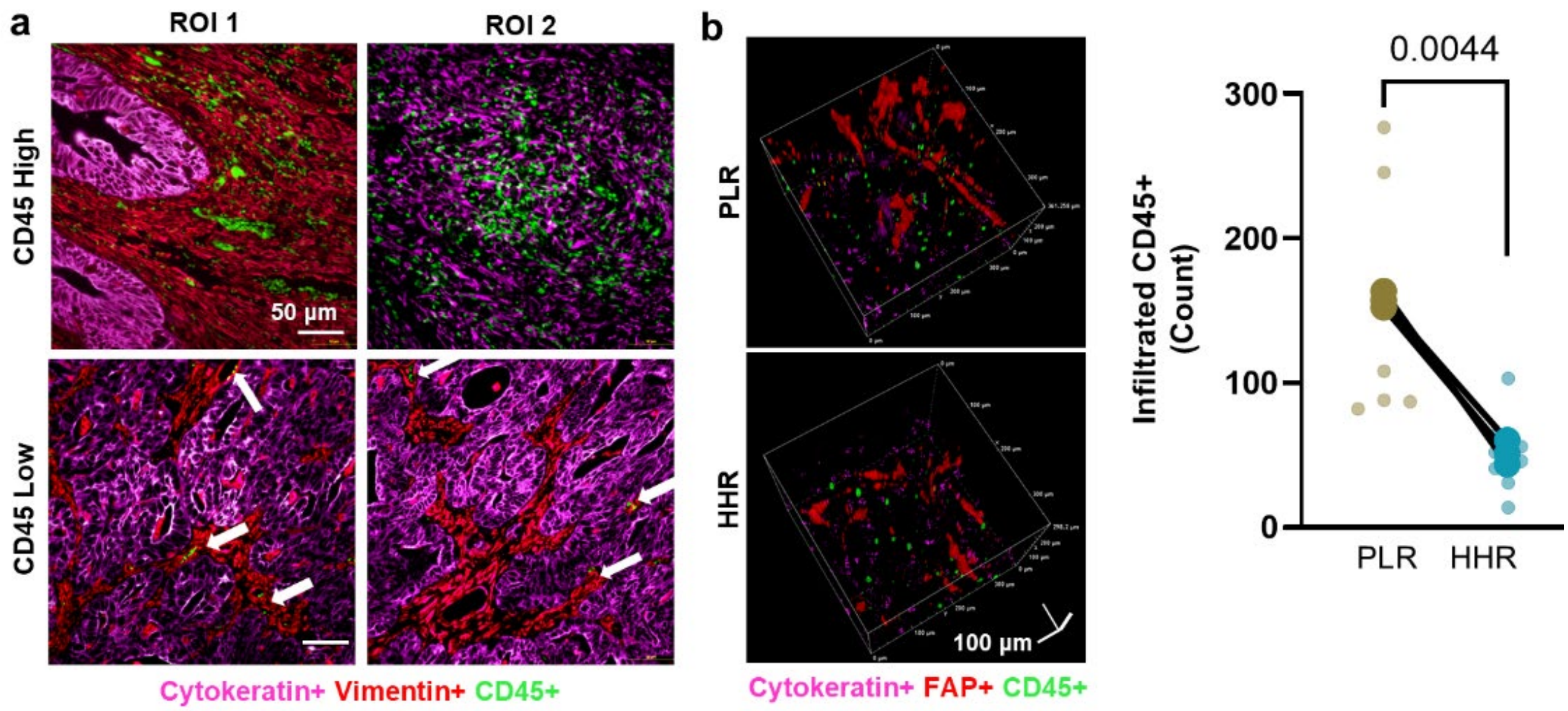

Supplementary Fig. 8. Spatial organization of immune infiltration in HGSC tumours is recapitulated in PLR and HHR 3D models. a Representative immunofluorescence images of two regions of interest (ROIs) from HGSC tumours with areas of low and high CD45 infiltration. Sections were stained for cytokeratin⁺ tumours cells (magenta), vimentin⁺ stromal cells (red), and CD45⁺ immune cells (green). White arrows indicate sparse CD45⁺ immune cell infiltration. Scale=50 µm. **b** Confocal 3D view reconstructions showing infiltration of CD45⁺ immune cells (green) within PLR and HHR 3D models generated from dissociated HGSC tumours, containing cytokeratin⁺ tumours cells (magenta) and FAP⁺ fibroblasts (red) and corresponding quantification of CD45+ cells (counts)**.** Scale=100µm**.** Data are represented as donor-paired superplots with biological replicates shown as larger, darker symbols (n=3 PLR/HHR cultures from different PBMC donors) and technical replicates as smaller, lighter symbols (n=3), paired t-test two-tailed. Source data are provided as a Source Data file.

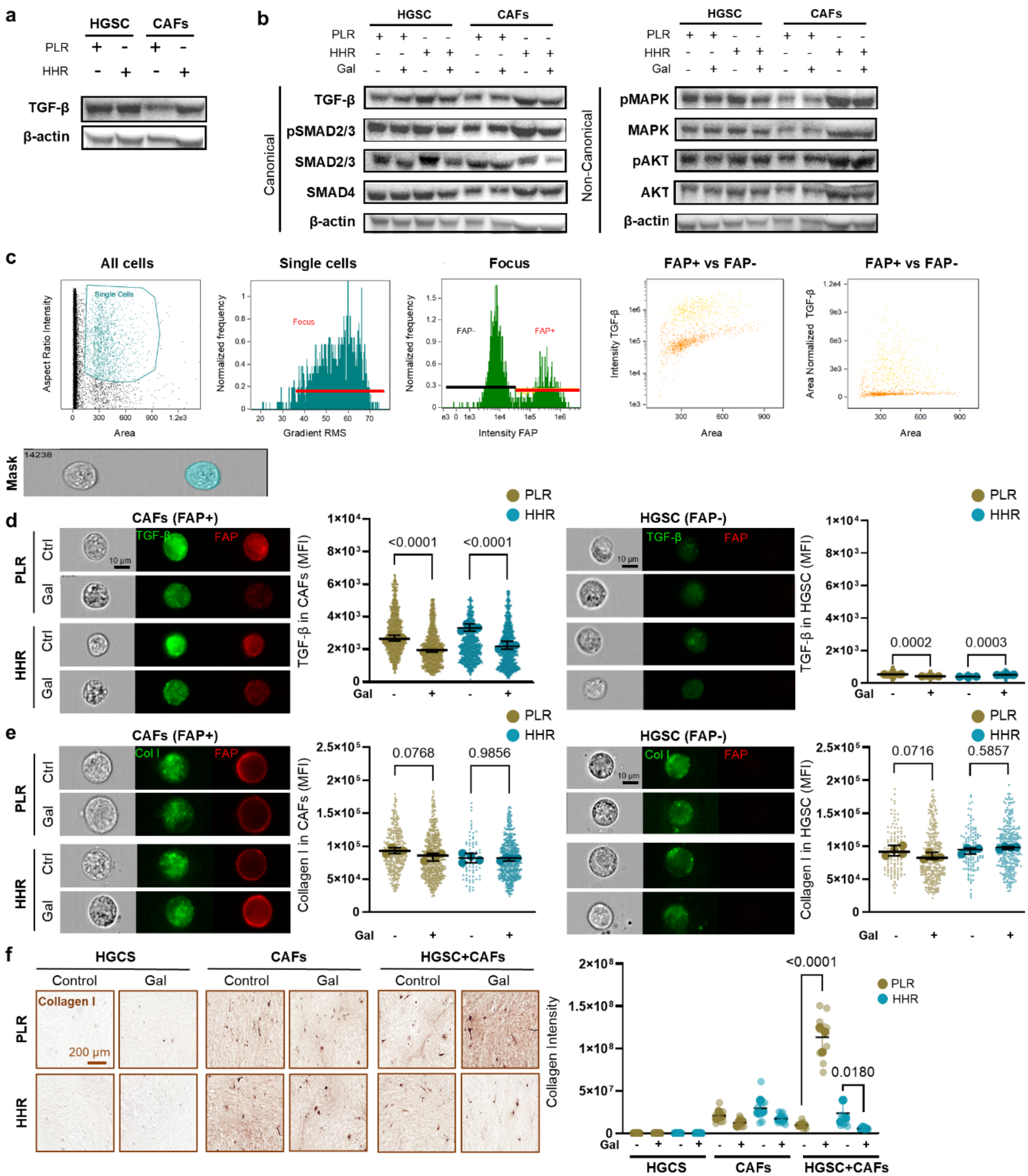

Supplementary Fig.9. Galunisertib decreases hypoxia-induced CAF-driven TGF-β signaling. **a** Western blot images of TGF-β in monocultures. n=3. **b** Western blot of canonical and non-canonical TGF-β signaling molecules in cells cultured in the presence or absence of galunisertib (Gal) in monocultures. n=3. **c** Gating strategy and mask used to detect cells in imaging flow cytometry. **d** Representative images of TGF-β (green) and **e** collagen I (green) detected by imaging flow cytometry in CAFs (FAP+, red) or KURAMOCHI (FAP-) and quantification of green mean fluorescent intensity (MFI). Scale=10μm. Median±CI95%. Data are represented as superplots with biological replicates shown as larger, darker symbols (n=4 PLR/HHR cultures from different days) and technical replicates as smaller, lighter symbols (n=20 individual cells/images minimum). **f** Representative IHC images of collagen I and quantification of positive signal. Mean±SD. Data are represented as superplots with biological replicates shown as larger, darker symbols (n=3 PLR/HHR cultures from different days) and technical replicates as smaller, lighter symbols (n=3). Scale=200μm. d,e ANOVA multi-comparisons Tukey test on the medians and f on the means. Source data are provided as a Source Data file.

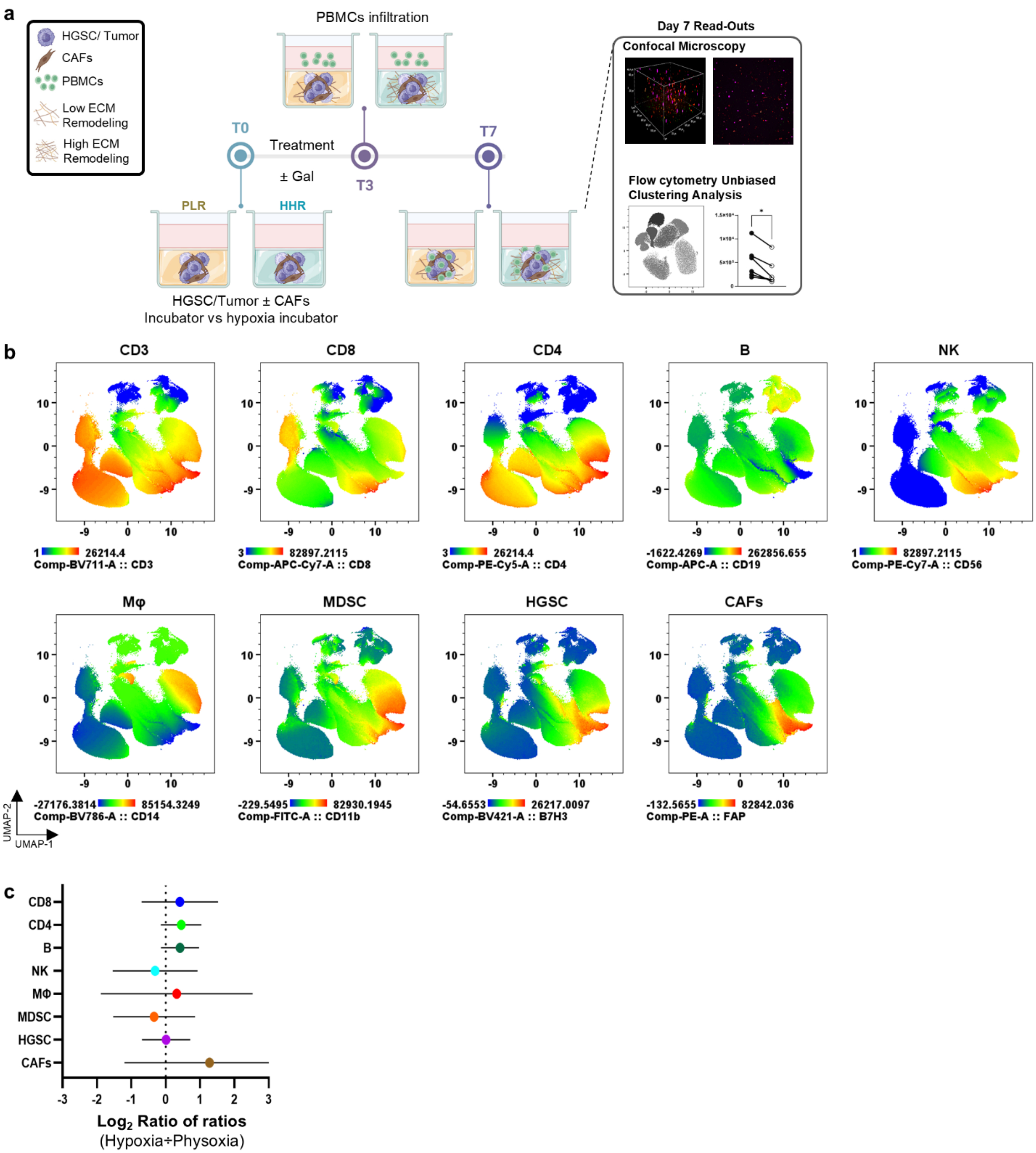

Supplementary Fig. 10. Assessment of galunisertib preincubation in the HGSC patient-derived model. a Schematic representation of infiltration studies in the presence or absence of galunisertib (Gal). Created in BioRender. **b** Heatmaps showing the expression of the surface markers used to detect the immune cell populations in dimensionality reduction analysis by flow cytometry in the infiltration of autologous PBMCs into matching HGSC primary tumours cocultured with CAFs. **c** Forests graphs representing $\log_2$ ratio of ratios of interactions model x treatment (hypoxia/physoxia) determined by linear mixed-effect model statistical analysis with donor as a random interceptor. Benjamini–Hochberg FDR. Source data are provided as a Source Data file.

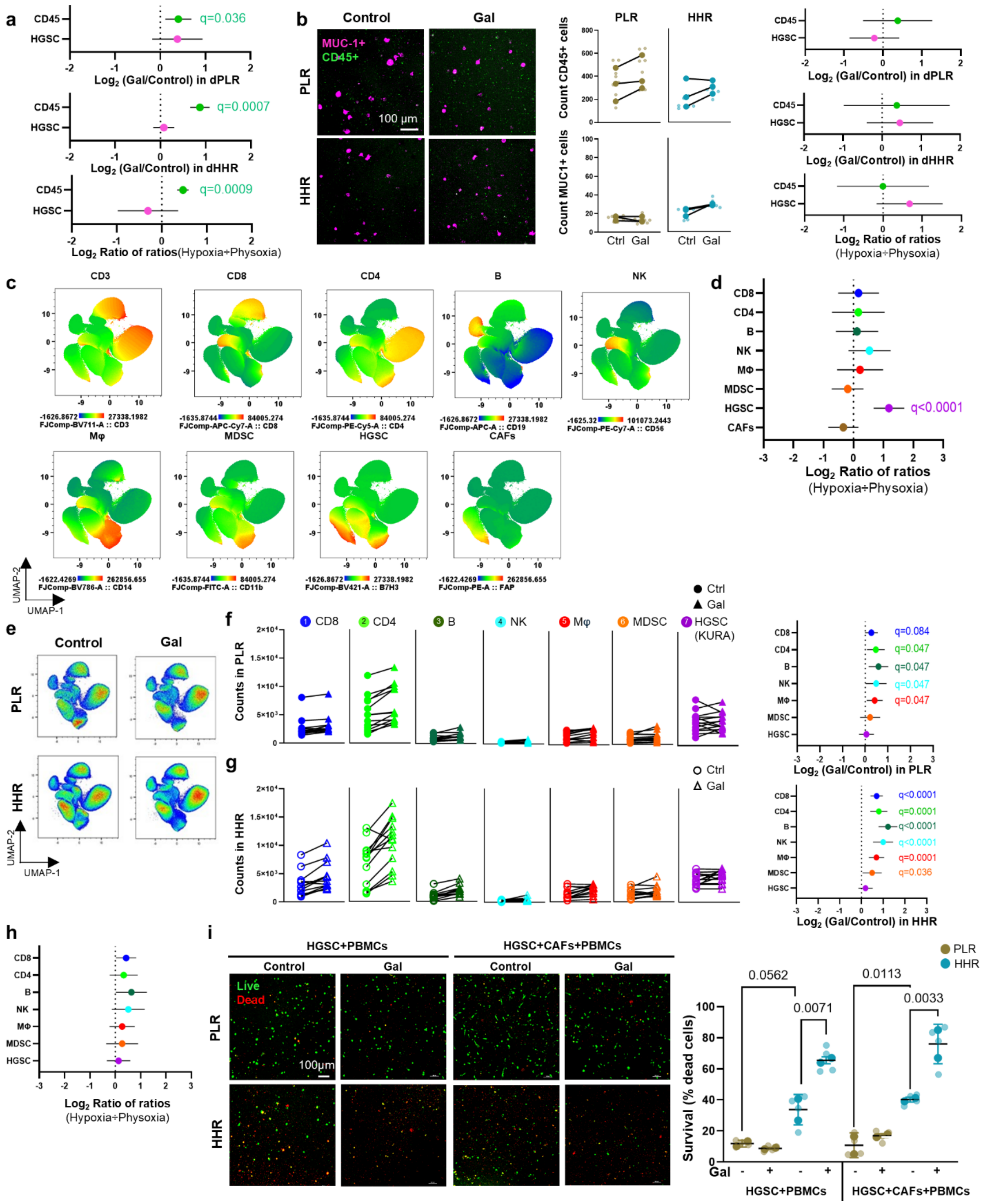

Supplementary Fig.11. Galunisertib attenuates the impaired immune infiltration in the HGSC cell line model. a-b Effects of galunisertib (Gal) on immune infiltration in KURAMOCHI co-culture (a) and monoculture (b) in PLR and HHR assessed by confocal microscopy. Forest plots show log₂ fold changes (Gal/Control) and model × treatment interaction effects (hypoxia/physoxia). Representative confocal images and donor-paired quantifications of infiltrated CD45⁺ PBMCs. Scale=100 µm. Data are represented as paired superplots with

biological replicates shown as larger, darker symbols (n=3 PLR/HHR cultures from different days) and technical replicates as smaller, lighter symbols (n=3). **c-d,** Effects of Gal on immune infiltration in KURAMOCHI co-culture and monoculture in PLR and HHR assessed by flow cytometry heatmaps (c) and forest plot of interaction in co-cultures (d). **e-h** Effects of Gal on immune infiltration in KURAMOCHI assessed by flow cytometry density plots (e), donor paired plots, forest plots log2 fold changes in PLR (**f**) and HHR (**g**), and forest plot of interaction (**h**). n=13 PBMCs from different donors. **i** Representative confocal images of Live/dead stain of infiltrated PBMCS in KURAMOCHI or KURAMOCHI co-cultures and survival quantification. Scale=100 µm; Mean±SD, Data are represented as superplots with biological replicates shown as larger, darker symbols (n=2 PLR/HHR cultures from different days) and technical replicates as smaller, lighter symbols (n=2). i one-way ANOVA. a,b,d,f,g,h linear mixed-effect model with Benjamini–Hochberg FDR. Source data are provided as a Source Data file.

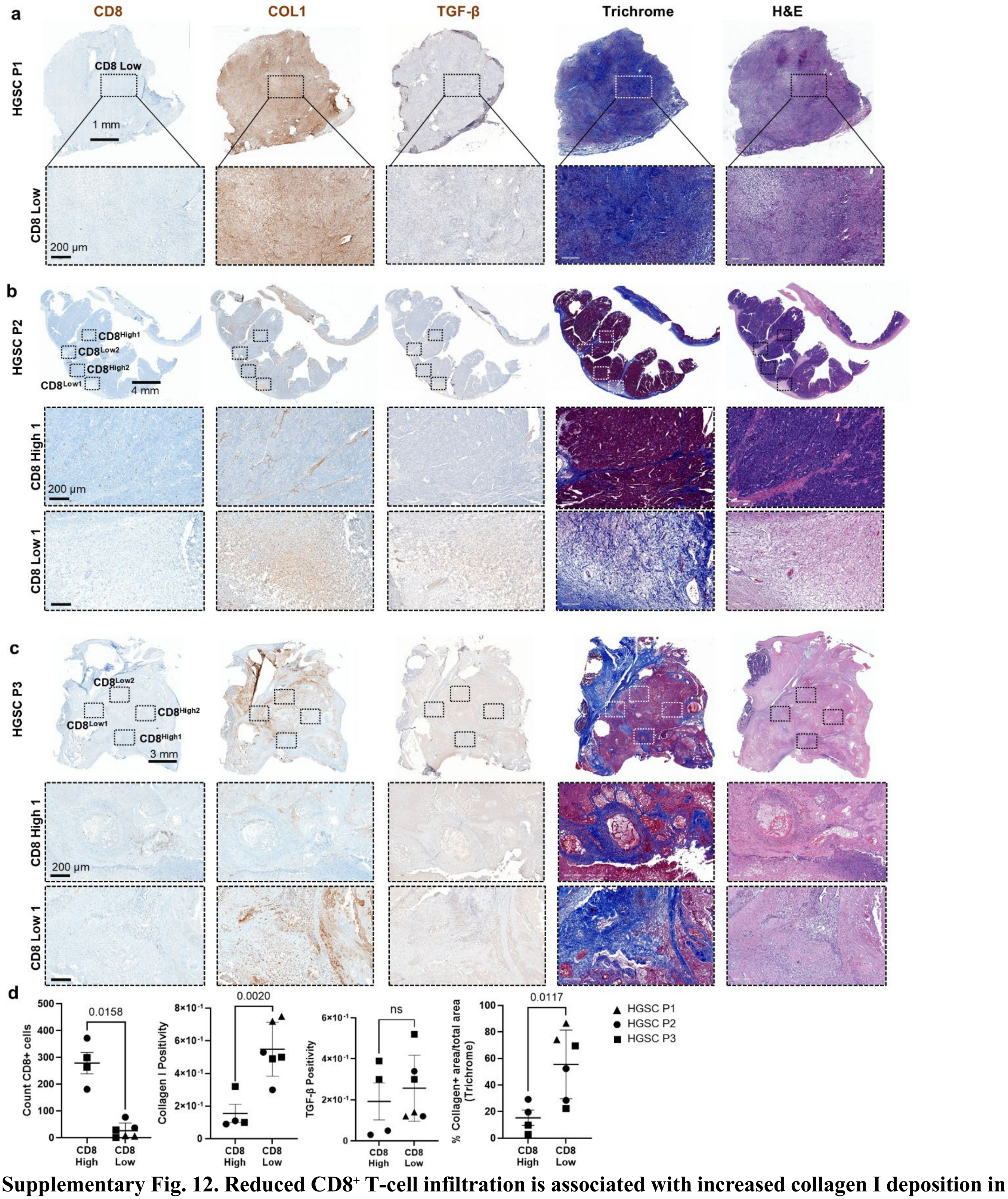

Supplementary Fig. 12. Reduced CD8⁺ T-cell infiltration is associated with increased collagen I deposition in HGSC tumours. a,b,c Representative IHC images of HGSC tumours sections for 3 patients (HGSC P1-3) stained for CD8+ T cells, collagen I, TGF-β, Masson's trichrome and H&E with magnified view of one Region of Interest (ROIs) with low CD8+ and high CD8+ T cell abundance/patient are shown, except for immunologically cold HGSC P1, in which only CD8 low ROI was identified. Scale= 200µm. **d** Quantification of counts (CD8+ T cells), positivity (collagen I and TGF-β), and area of coverage (% blue (collagen)/total area) in $CD8^{Low}$ and $CD8^{High}$ ROIs/patient. n=3, paired t-test two-tailed. ns=not significant. Source data are provided as a Source Data file.

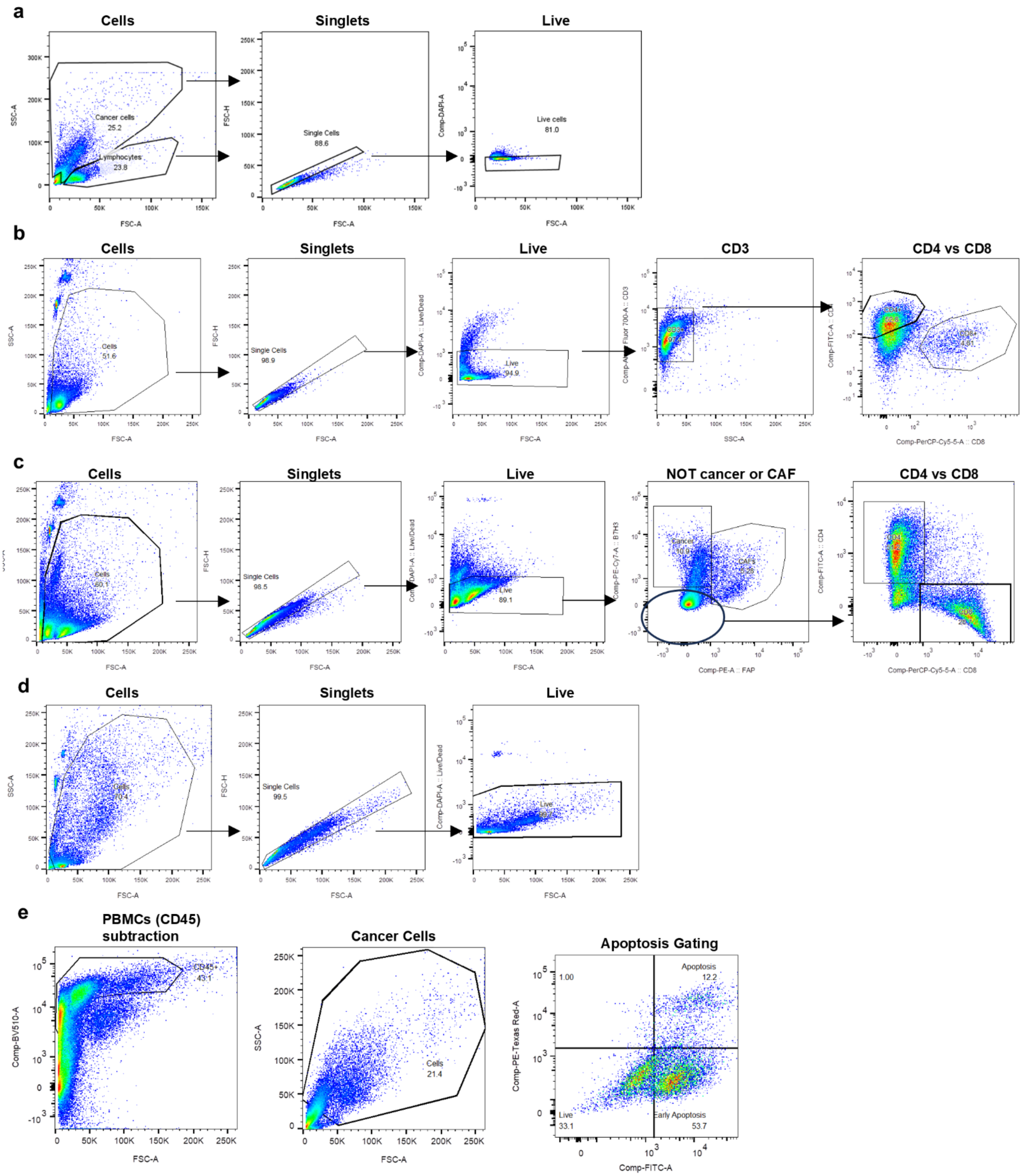

Supplementary Fig. 13. Gating strategies used for flow cytometry analysis. a Gating strategy to identify HIF expression in PLR/HHR studies presented on Fig. 1j and Suppl. Fig. 1f. **b** Gating strategy for intracellular immunophenotyping studies for CD4 and CD8 cells presented on Fig 3a and Suppl. Fig 6a. **c** Gating strategy for immunophenotyping functional studies for CD4 and CD8 cells presented on Fig 3c and Suppl. Fig 6b. **d** Gating strategy for infiltration studies presented on Fig 4c, 6abcfgh and Suppl. Fig 7b, 10b, 11cdefgh. **e** Gating strategy for apoptosis studies presented on Suppl. Fig. 6c.

Supplementary Table 1

Additional data of linear mixed effects models with donor as a random factor and multiplicity control

Fig 6 b,c & Supp Fig10c

	population	n_donors_u	effect_log	se_log	ci95_lo_log	ci95_hi_log	p_value	q_value	ratio	ratio_lo	ratio_hi	log2	log2_lo	log2_hi	model_con	optimizer_	emm_phys	emm_phys	emm_hypo	emm_hypo_drug
**PLR**	B cells	6	0.16570847	0.13853687	-0.105824	0.43724075	0.23164483	0.59495236	1.18022898	0.89958313	1.54842881	0.23906678	-0.152671	0.63080506	TRUE	MixedLM	1695.58195	2001.35519	1020.36465	1608.925
**PLR**	CAFs	6	0.48749964	0.61987005	-0.727446	1.70244494	0.43160121	0.68279688	1.62823995	0.48314152	5.48734727	0.70331332	-1.049482	2.45610888	TRUE	MixedLM	2052.93941	3343.30621	1184.08188	4675.505156440168
**PLR**	CD4	6	0.15378387	0.14760499	-0.135522	0.44308966	0.29747618	0.59495236	1.16623880	0.87326	1.55751197	0.22186322	-0.195517	0.63924325	TRUE	MixedLM	8669.13473	10110.45	5458.40809	8708.889433170632
**PLR**	CD8	6	0.093424	0.27827044	-0.451986	0.638834	0.73707431	0.73707431	1.09792693	0.63636290	1.89427060	0.13478204	-0.652078	0.92164243	TRUE	MixedLM	4524.91449	4968.12342	2016.36814	2948.981133365855
**PLR**	HGSC	6	0.34025379	0.17515591	-0.003052	0.68355938	0.052067	0.40702430	1.40530420	0.99695285	1.98091604	0.49088246	-0.004403	0.98616773	TRUE	MixedLM	3682.93380	5176.04765	2707.37	3844.5738618529285
**PLR**	MDSC	6	0.15701243	0.29732980	-0.425754	0.739779	0.59744727	0.68279688	1.17001016	0.65327704	2.09547205	0.22652106	-0.614233	1.06727528	TRUE	MixedLM_failed: Singular matrix | Fallback: OLS with donor fixed effects				
**PLR**	Macrophag	6	-0.904011	0.552439	-1.986791	0.17876973	0.10175607	0.40702430	0.40494227	0.13713475	1.19574531	-1.304212	-2.866334	0.25791021	TRUE	MixedLM	403.674890	162.869969	713.085580	360.79106150113586
**PLR**	NK cells	6	0.175608	0.30818025	-0.428426	0.77964106	0.56879868	0.68279688	1.19197044	0.65153411	2.18068939	0.25334846	-0.618087	1.12478429	TRUE	MixedLM	301.756287	359.876545	377.812083	364.3037004256766
**HHR**	B cells	6	0.45504770	0.13853687	0.18351548	0.72658	0.001021	0.006211	1.57624867	1.20143357	2.06799604	0.65649515	0.26475688	1.04823342	TRUE	MixedLM	1695.58195	2001.35519	1020.36465	1608.925
**HHR**	CAFs	6	1.37273919	0.61987005	0.15779389	2.58768449	0.02679030	0.071441	3.94614517	1.17092483	13.2989421	1.98044403	0.22764847	3.73323959	TRUE	MixedLM	2052.93941	3343.30621	1184.08188	4675.505156440168
**HHR**	CD4	6	0.46711872	0.14760499	0.17781292	0.756425	0.001553	0.006211	1.59539079	1.19460182	2.13064449	0.67390986	0.25652983	1.09128989	TRUE	MixedLM	8669.13473	10110.45	5458.40809	8708.889433170632
**HHR**	CD8	6	0.38000500	0.27827044	-0.165405	0.92541507	0.17206579	0.27530526	1.46229191	0.84755033	2.52291523	0.54823134	-0.238629	1.335092	TRUE	MixedLM	4524.91449	4968.12342	2016.36814	2948.981133365855
**HHR**	HGSC	6	0.35057590	0.17515591	0.00727	0.69388149	0.045338	0.090675	1.41988503	1.00729680	2.00146917	0.50577411	0.01048884	1.00105939	TRUE	MixedLM	3682.93380	5176.04765	2707.37	3844.5738618529285
**HHR**	MDSC	6	-0.07549	0.29732980	-0.658257	0.50727591	0.79957682	0.90620668	0.92728854	0.51775303	1.66076097	-0.10891	-0.949664	0.731844	TRUE	MixedLM_failed: Singular matrix | Fallback: OLS with donor fixed effects				
**HHR**	Macrophag	6	-0.679936	0.552439	-1.762716	0.40284454	0.21840174	0.291202	0.50664944	0.17157814	1.49607430	-0.98094	-2.543062	0.58118183	TRUE	MixedLM	403.674890	162.869969	713.085580	360.79106150113586
**HHR**	NK cells	6	-0.036311	0.30818025	-0.640344	0.56772209	0.90620668	0.90620668	0.96434014	0.527111	1.76424369	-0.052386	-0.923822	0.81904985	TRUE	MixedLM	301.756287	359.876545	377.812083	364.3037004256766

HHR÷PLR	population	n_donors_u	interaction	se_log	ci95_lo_log	ci95_hi_log	p_value	q_value	ror	ror_lo	ror_hi	log2_ror	log2_lo	log2_hi	model_con	optimizer_note
**HHR÷PLR**	B cells	6	0.28933929	0.19592073	-0.094665	0.673344	0.13972438	0.55889754	1.33554479	0.90967731	1.96078308	0.41742836	-0.136573	0.97142994	TRUE	MixedLM
**HHR÷PLR**	CAFs	6	0.88523955	0.876629	-0.832953	2.60343167	0.31258021	0.83354723	2.42356488	0.43476371	13.5100205	1.27713070	-1.201697	3.755958	TRUE	MixedLM
**HHR÷PLR**	CD4	6	0.31333485	0.20874498	-0.095805	0.72247502	0.13334487	0.55889754	1.36797952	0.90864089	2.05952427	0.452047	-0.138218	1.04231113	TRUE	MixedLM
**HHR÷PLR**	CD8	6	0.28658121	0.39353383	-0.484745	1.05790752	0.46647580	0.83573004	1.33186633	0.61585416	2.88033764	0.41344929	-0.699339	1.52623793	TRUE	MixedLM
**HHR÷PLR**	HGSC	6	0.010322	0.24770786	-0.475185	0.49582953	0.96676137	0.96676137	1.01037556	0.62176982	1.64185965	0.01489165	-0.685547	0.71533081	TRUE	MixedLM
**HHR÷PLR**	MDSC	6	-0.232503	0.42048783	-1.056659	0.59165323	0.580307	0.83573004	0.792547	0.34761522	1.80697329	-0.335431	-1.524437	0.85357518	TRUE	MixedLM_failed: Singular matrix | Fallback: OLS with donor fixed effects
**HHR÷PLR**	Macrophag	6	0.22407481	0.781267	-1.307208	1.75535767	0.774258	0.88486640	1.25116461	0.27057443	5.78551672	0.32327162	-1.885903	2.53244581	TRUE	MixedLM
**HHR÷PLR**	NK cells	6	-0.211919	0.43583269	-1.066151	0.64231310	0.62679753	0.83573004	0.80903025	0.34433128	1.90087272	-0.305734	-1.538131	0.92666193	TRUE	MixedLM

Fig 6e and Suppl 11a

	population	n_donors_u	effect_log	se_log	ci95_lo_log	ci95_hi_log	p_value	q_value	ratio	ratio_lo	ratio_hi	log2	log2_lo	log2_hi	hedges_g	model_use	optimizer_	emm_phys	emm_phys	emm_hypo	emm_hypo_drug
**PLR**	CD45	5	0.27850799	0.072153	0.07818	0.47883582	0.01814750	0.036295	1.32115716	1.08131745	1.61419409	0.40180209	0.11279012	0.690814	1.38098972	donor-level	paired (trim	461.834909	610.477655	379.985854	696.7076452759262
**PLR**	Cancer	5	0.26441774	0.13946552	-0.122801	0.65163612	0.13085088	0.13085088	1.30267227	0.88443997	1.918677	0.38147417	-0.177164	0.940112	0.67831078	donor-level	paired (trim	14.9926504	19.8331822	18.5427099	19.586619188528218
**HHR**	CD45	5	0.60503792	0.053981	0.45516242	0.75491341	0.000361	0.000722	1.83132165	1.57642941	2.12742731	0.87288520	0.656661	1.08910983	4.01001540	donor-level	paired (trim	461.834909	610.477655	379.985854	696.7076452759262
**HHR**	Cancer	5	0.052039	0.057693	-0.108142	0.21222022	0.41806071	0.41806071	1.05341680	0.8975	1.23642015	0.075076	-0.156016	0.30616907	0.32270911	donor-level	paired (trim	14.9926504	19.8331822	18.5427099	19.586619188528218

HHR÷PLR	population	n_donors_u	interaction	se_log	ci95_lo_log	ci95_hi_log	p_value	q_value	ror	ror_lo	ror_hi	log2_ror	log2_lo	log2_hi	hedges_g	model_use	optimizer_	emm_phys	emm_phys	emm_hypo	emm_hypo_drug
**HHR÷PLR**	CD45	5	0.32652992	0.031522	0.23901163	0.41404822	0.00049	0.00098	1.38614973	1.26999331	1.51293008	0.47108310	0.34482089	0.59734532	3.70610924	donor-level	paired (trim	461.834909	610.477655	379.985854	696.7076452759262
**HHR÷PLR**	Cancer	5	-0.212379	0.16718209	-0.676551	0.25179313	0.272825	0.272825	0.80865835	0.50836750	1.28632991	-0.306398	-0.976056	0.36326071	-0.454492	donor-level	paired (trim	14.9926504	19.8331822	18.5427099	19.586619188528218

ICC by condition

population	oxygen	treatment	icc	optimizer_note
CD45	physioxia	vehicle	0.57912022	MixedLM
CD45	physioxia	drug	0.71331233	MixedLM
CD45	hypoxia	vehicle	0.64858145	MixedLM
CD45	hypoxia	drug	0.63101347	MixedLM
Cancer	physioxia	vehicle	0.34358167	MixedLM
Cancer	physioxia	drug	0.51223782	MixedLM
Cancer	hypoxia	vehicle	0.70464766	MixedLM
Cancer	hypoxia	drug	0.76901494	MixedLM

Fig 6 g,h and Supp Fig 11d

	population	n_donors_u	effect_log	se_log	ci95_lo_log	ci95_hi_log	p_value	q_value	ratio	ratio_lo	ratio_hi	log2	log2_lo	log2_hi	model_con	optimizer_	emm_phys	emm_phys	emm_hypo	emm_hypo_drug
**PLR**	B cells	12	0.14782380	0.17635381	-0.19783	0.49347727	0.40190553	0.64304885	1.15930861	0.82050959	1.63800211	0.21326466	-0.285408	0.71193722	TRUE	MixedLM	799.580354	927.119699	814.159235	1026.6437562790572
**PLR**	CAF	12	1.37728542	0.12552246	1.13126140	1.62330945	0	0	3.96412609	3.09956383	5.06984097	1.98700285	1.63206521	2.34194049	TRUE	MixedLM	1032.40166	4095.53449	1437.85452	4515.243
**PLR**	CD4	12	0.072401	0.21981370	-0.358434	0.50323559	0.74187368	0.80177616	1.07508607	0.69876964	1.65406450	0.10445216	-0.517111	0.72601550	TRUE	MixedLM_fa	5655.42040	6080.13880	5036.67816	6074.7628306387405
**PLR**	CD8	12	0.04593931	0.17053811	-0.288315	0.38019402	0.78763837	0.80177616	1.04701087	0.74952515	1.46256833	0.066276	-0.415951	0.54850403	TRUE	MixedLM_fa	2167.02146	2268.94204	2139.21849	2521.875
**PLR**	HGSC	12	-0.032209	0.12829689	-0.283671	0.219253	0.80177616	0.80177616	0.96830436	0.75301455	1.24514637	-0.046467	-0.40925	0.31631534	TRUE	MixedLM	2614.34029	2531.44541	1377.07823	3030.396420640597
**PLR**	MDSC	12	0.48594325	0.13252881	0.22618677	0.74569974	0.000246	0.000983	1.62570774	1.25380982	2.10791591	0.70106793	0.32631853	1.07581732	TRUE	MixedLM	642.1227	1044.52961	637.279443	907.9572117185319
**PLR**	Macrophag	12	0.23647098	0.19182990	-0.139516	0.61245760	0.21768331	0.49507930	1.26677080	0.86977942	1.84496000	0.34115551	-0.201279	0.88358954	TRUE	MixedLM	1028.23452	1302.80424	847.842370	1253.813615814945
**PLR**	NK cells	12	0.20318728	0.17571496	-0.141214	0.54758861	0.24753968	0.49507930	1.22530192	0.86830343	1.72907851	0.29313729	-0.203729	0.79000338	TRUE	MixedLM_fa	166.930314	204.765338	138.556942	247.28968145593439
**HHR**	B cells	12	0.23164037	0.17635381	-0.114013	0.577294	0.18901521	0.25202028	1.26066627	0.89224626	1.78121160	0.33418641	-0.164486	0.83285896	TRUE	MixedLM	799.580354	927.119699	814.159235	1026.6437562790572
**HHR**	CAF	12	1.14383310	0.12552246	0.89780913	1.38985718	0	0	3.13877671	2.45422035	4.01427671	1.65020242	1.29526478	2.00514006	TRUE	MixedLM	1032.40166	4095.53449	1437.85452	4515.243
**HHR**	CD4	12	0.18736225	0.21981370	-0.243473	0.61819712	0.39400960	0.39400960	1.20606411	0.78390094	1.85557965	0.27030659	-0.351257	0.89186992	TRUE	MixedLM_fa	5655.42040	6080.13880	5036.67816	6074.7628306387405
**HHR**	CD8	12	0.16449112	0.17053811	-0.169764	0.49874583	0.334774	0.38259935	1.17879310	0.843864	1.64665479	0.237311	-0.244917	0.71953813	TRUE	MixedLM_fa	2167.02146	2268.94204	2139.21849	2521.875
**HHR**	HGSC	12	0.78833343	0.12829689	0.53687152	1.03979533	8.01638977	3.20655590	2.19972731	1.71064676	2.82863804	1.13732473	0.77454188	1.50010757	TRUE	MixedLM	2614.34029	2531.44541	1377.07823	3030.396420640597
**HHR**	MDSC	12	0.35352183	0.13252881	0.093765	0.61327831	0.007642	0.015283	1.42407408	1.09830199	1.84647481	0.51002419	0.13527480	0.884774	TRUE	MixedLM	642.1227	1044.52961	637.279443	907.9572117185319
**HHR**	Macrophag	12	0.39086882	0.19182990	0.01488220	0.76685543	0.041592	0.066547	1.47826458	1.01499349	2.15298540	0.563905	0.02147048	1.10633853	TRUE	MixedLM	1028.23452	1302.80424	847.842370	1253.813615814945
**HHR**	NK cells	12	0.57612343	0.17571496	0.23172210	0.92052475	0.001043	0.002781	1.77912813	1.26076931	2.51060751	0.83117041	0.33430432	1.32803650	TRUE	MixedLM_fa	166.930314	204.765338	138.556942	247.28968145593439

HHR÷PLR	population	n_donors_u	interaction	se_log	ci95_lo_log	ci95_hi_log	p_value	q_value	ror	ror_lo	ror_hi	log2_ror	log2_lo	log2_hi	model_con	optimizer_note
**HHR÷PLR**	B cells	12	0.083817	0.24940195	-0.405011	0.572644	0.73681792	0.73681792	1.08742940	0.66696929	1.77294924	0.12092174	-0.584308	0.82615123	TRUE	MixedLM
**HHR÷PLR**	CAF	12	-0.233452	0.17751556	-0.581383	0.11447824	0.18847337	0.50259560	0.79179539	0.55912468	1.12128824	-0.3368	-0.838758	0.16515719	TRUE	MixedLM
**HHR÷PLR**	CD4	12	0.11496152	0.31086352	-0.494331	0.72425404	0.71152145	0.73681792	1.12183027	0.60997885	2.06319148	0.16585442	-0.713169	1.04487772	TRUE	MixedLM_failed or singular: Singular matrix
**HHR÷PLR**	CD8	12	0.11855180	0.24117732	-0.354156	0.591259	0.62303428	0.73681792	1.12586519	0.70176560	1.80626170	0.17103410	-0.510939	0.85300693	TRUE	MixedLM_failed or singular: Singular matrix
**HHR÷PLR**	HGSC	12	0.82054224	0.18143920	0.46492140	1.17616308	6.11398471	4.89118777	2.27173134	1.59188907	3.24191138	1.18379223	0.67073980	1.69684465	TRUE	MixedLM
**HHR÷PLR**	MDSC	12	-0.132421	0.18742405	-0.499773	0.23492971	0.47985625	0.73681792	0.87597176	0.60666862	1.26481987	-0.191044	-0.721019	0.33893193	TRUE	MixedLM
**HHR÷PLR**	Macrophag	12	0.15439783	0.27128845	-0.377328	0.68612320	0.56926932	0.73681792	1.16695505	0.68569145	1.98600127	0.22274899	-0.544369	0.98986655	TRUE	MixedLM
**HHR÷PLR**	NK cells	12	0.37293614	0.24849848	-0.114121	0.85999317	0.13341810	0.50259560	1.45199161	0.89215009	2.36314456	0.53803312	-0.164642	1.24070788	TRUE	MixedLM_failed or singular: Singular matrix

Fig 7h

	population	n_donors_u	effect_log	se_log	ci95_lo_log	ci95_hi_log	p_value	q_value	ratio	ratio_lo	ratio_hi	log2	log2_lo	log2_hi	hedges_g	model_use	optimizer_	emm_phys	emm_phys	emm_hypo	emm_hypo_drug
**PLR**	Immune	3	0.21947540	0.31239037	-1.124632	1.56358274	0.55508730	0.55508730	1.24542329	0.32477202	4.77590148	0.31663616	-1.622501	2.25577307	0.23178731	donor-level	paired (trim	324.840364	404.809180	113.646540	274.94535102586747
**HHR**	Immune	3	0.87834901	0.18607448	0.077735	1.67896289	0.04206702	0.12620108	2.40692262	1.08083633	5.35999422	1.26718976	0.11214808	2.42223144	1.55733347	donor-level	paired (trim	324.840364	404.809180	113.646540	274.94535102586747
**HHR÷PLR**	population	n_donors_u	interaction_	se_log	ci95_lo_log	ci95_hi_log	p_value	q_value	ror	ror_lo	ror_hi	log2_ror	log2_lo	log2_hi	hedges_g	model_use	optimizer_note				
	Immune	3	0.65887354	0.36411955	-0.907806	2.22555355	0.21209042	0.31813564	1.93261410	0.40340814	9.25860650	0.95055359	-1.309688	3.21079507	0.596979	donor-level	paired (trimmed mean on log1p)				

ICC by condition

oxygen	treatment	icc	optimizer_note
physioxia	vehicle	0.75628653	MixedLM
physioxia	drug	0.0004	MixedLM
hypoxia	vehicle	0.088625	MixedLM
hypoxia	drug	0.006983	MixedLM

Supplementary Fig 11b

	population	n_donors_u	effect_log	se_log	ci95_lo_log	ci95_hi_log	p_value	q_value	ratio	ratio_lo	ratio_hi	log2	log2_lo	log2_hi	hedges_g	model_use	optimizer_	emm_phys	emm_phys	emm_hypo	emm_hypo_drug
**PLR**	CD45	3	0.26272459	0.14311189	-0.353036	0.87848530	0.20780599	0.27649707	1.300469	0.70255177	2.40725084	0.37903147	-0.509324	1.26738648	0.60565643	donor-level	paired (trim	302.000687	393.042853	232.308842	302.00617371525516
**PLR**	Cancer	3	-0.152751	0.10305767	-0.596172	0.29067028	0.27649707	0.27649707	0.85834333	0.55091624	1.33732356	-0.220373	-0.860095	0.41934857	-0.488996	donor-level	paired (trim	15.1571990	12.8684241	21.0292390	29.232817369128234
**HHR**	CD45	3	0.26139	0.21841275	-0.678364	1.20114431	0.35401718	0.35401718	1.29873420	0.50744643	3.32391837	0.37710619	-0.978673	1.73288495	0.39483211	donor-level	paired (trim	302.000687	393.042853	232.308842	302.00617371525516
**HHR**	Cancer	3	0.31655735	0.13715779	-0.273585	0.9067	0.14734040	0.29468081	1.37239495	0.76064769	2.47613704	0.45669573	-0.3947	1.30809116	0.76143573	donor-level	paired (trim	15.1571990	12.8684241	21.0292390	29.232817369128234

HHR÷PLR	population	n_donors_	interaction_	se_log	ci95_lo_log	ci95_hi_log	p_value	q_value	ror	ror_lo	ror_hi	log2_ror	log2_lo	log2_hi	hedges_g	model_use	optimizer_r	emm_phys	emm_phys	emm_hypo_	emm_hypo_drug
HHR÷PLR	CD45	3	-0.001334	0.1885247	-0.812491	0.8098220	0.9949947	0.9949947	0.998666	0.4437512	2.2475079	-0.001925	-1.172177	1.1683262	-0.002335	donor-level	paired (trim	302.00068	393.04285	232.30884	302.00617371525516
	Cancer	3	0.4693084	0.1357424	-0.114744	1.0533608	0.074438	0.1488769	1.5988881	0.8915943	2.8672715	0.677069	-0.165541	1.5196785	1.1406283	donor-level	paired (trim	15.157199	12.868424	21.029239	29.232817369128234

ICC by condition

population	oxygen	treatment	icc	optimizer_note
CD45	physioxia	vehicle	0.8047916	MixedLM
CD45	physioxia	drug	0.8180492	MixedLM
CD45	hypoxia	vehicle	0.9654058	MixedLM
CD45	hypoxia	drug	0.6875205	MixedLM
Cancer	physioxia	vehicle	0.6707127	MixedLM
Cancer	physioxia	drug	0.2532539	MixedLM
Cancer	hypoxia	vehicle	0.3799254	MixedLM
Cancer	hypoxia	drug	5.1407980	MixedLM

Suppl Fig 11 f,g,h

	population	n_donors_	effect_log	se_log	ci95_lo_log	ci95_hi_log	p_value	q_value	ratio	ratio_lo	ratio_hi	log2	log2_lo	log2_hi	optimizer_note
PLR	B cells	13	0.4063844	0.1533089	0.1058988	0.7068699	0.008031	0.047427	1.5013795	1.1117093	2.0276348	0.5862887	0.1527796	1.0197978	MixedLM
PLR	CD4	13	0.3242187	0.1384206	0.052914	0.5955231	0.019167	0.047427	1.3829497	1.0543392	1.8139796	0.4677487	0.076339	0.8591582	MixedLM
PLR	CD8	13	0.1882796	0.1000155	-0.007751	0.3843102	0.059768	0.083675	1.2071711	0.9922791	1.4686009	0.2716301	-0.011182	0.5544424	MixedLM
PLR	HGSC	13	0.038116	0.1162292	-0.189694	0.2659248	0.7429610	0.7429610	1.0388512	0.8272125	1.3046369	0.0549891	-0.27367	0.383648	MixedLM
PLR	MDSC	13	0.1500098	0.1568844	-0.157484	0.4575033	0.3389810	0.3954778	1.1618456	0.854291	1.5801240	0.2164184	-0.227201	0.6600378	MixedLM
PLR	Macrophag	13	0.2890378	0.1245704	0.04488	0.5331958	0.020326	0.047427	1.3351422	1.0459021	1.7043704	0.4169934	0.064748	0.7692389	MixedLM
PLR	NK cells	13	0.3344627	0.16299	0.0150031	0.6539224	0.040165	0.070288	1.3971895	1.0151162	1.9230691	0.4825277	0.0216449	0.9434106	MixedLM

HHR	population	n_donors_	effect_log	se_log	ci95_lo_log	ci95_hi_log	p_value	q_value	ratio	ratio_lo	ratio_hi	log2	log2_lo	log2_hi	optimizer_note
HHR	B cells	13	0.846215	0.1533089	0.5457297	1.1467008	3.3963262	2.3774283	2.3308087	1.7258672	3.1477908	1.2208306	0.7873215	1.6543396	MixedLM
HHR	CD4	13	0.5534895	0.1384206	0.2821851	0.8247940	6.3716529	0.000112	1.7393119	1.3260242	2.2814107	0.7985166	0.4071071	1.1899262	MixedLM
HHR	CD8	13	0.48499	0.1000155	0.2889597	0.6810208	1.2399353	4.3397736	1.6241592	1.3350379	1.9758937	0.6996930	0.4168807	0.982505	MixedLM
HHR	HGSC	13	0.1295251	0.1162292	-0.098284	0.3573343	0.2651101	0.2651101	1.1382877	0.9063913	1.4295137	0.1868652	-0.141794	0.5155245	MixedLM
HHR	MDSC	13	0.338485	0.1568844	0.030992	0.6459786	0.0309632	0.0361238	1.4028209	1.0314768	1.9078532	0.4883308	0.044711	0.9319502	MixedLM
HHR	Macrophag	13	0.4778148	0.1245704	0.233657	0.7219728	0.000125	0.000175	1.6125468	1.2632109	2.05849	0.6893411	0.3370955	1.0415866	MixedLM
HHR	NK cells	13	0.6914917	0.1629896	0.3720321	1.0109513	2.2099322	5.1565086	1.9966918	1.4506795	2.7482144	0.9976117	0.5367288	1.4584945	MixedLM

HHR÷PLR	population	n_donors_	interaction_	se_log	ci95_lo_log	ci95_hi_log	p_value	q_value	ror	ror_lo	ror_hi	log2_ror	log2_lo	log2_hi	optimizer_note
HHR÷PLR	B cells	13	0.4398308	0.2168116	0.0148800	0.8647816	0.042496	0.1487354	1.5524446	1.0149913	2.3744876	0.6345418	0.021467	1.2476162	MixedLM
HHR÷PLR	CD4	13	0.2292708	0.1957563	-0.154412	0.6129532	0.2415161	0.3974804	1.2576826	0.8569193	1.8458747	0.3307679	-0.222769	0.8843046	MixedLM
HHR÷PLR	CD8	13	0.2967105	0.1414433	0.019482	0.5739396	0.035929	0.1487354	1.3454258	1.0196725	1.7752471	0.4280628	0.0281059	0.8280198	MixedLM
HHR÷PLR	HGSC	13	0.09141	0.1643729	-0.230761	0.4135804	0.5781350	0.5781350	1.0957176	0.7939289	1.5122225	0.1318761	-0.332918	0.5966705	MixedLM
HHR÷PLR	MDSC	13	0.1884752	0.2218681	-0.246386	0.6233367	0.395607	0.4615419	1.2074072	0.7816202	1.8651412	0.2719123	-0.35546	0.8992849	MixedLM
HHR÷PLR	Macrophag	13	0.1887770	0.1761691	-0.156515	0.5340685	0.2839146	0.3974804	1.2077716	0.8551191	1.7058586	0.2723476	-0.225803	0.7704981	MixedLM
HHR÷PLR	NK cells	13	0.3570289	0.2305021	-0.094755	0.808813	0.1214013	0.2832697	1.4290772	0.9095956	2.2452416	0.5150839	-0.136703	1.1668707	MixedLM

Supplementary Table 2. List of antibodies/staining dyes and cytokines used in the study

Technique	Assay	Antibody	Source	Product	Dilution
IHC	3D models	anti-HIF1α	Rabbit	HPA001275, Sigma Aldrich	1:100
		anti-collagen I	Rabbit	HPA011795, Sigma Aldrich	1:50
		anti-α-SMA	Mouse	NBP2-33006, Novus	1:100
	dECM	anti-collagen I	Rabbit	HPA011795, Sigma Aldrich	1:50
		anti-fibronectin	Rabbit	ab 2413, Abcam	1:100
		anti-collagen I	Rabbit	HPA011795, Sigma Aldrich	1:50
	Tumors	anti-CD8	Rabbit	SP16, Cell Marque	1:50
		anti-TGF-β	Mouse	sc 130348, Santa Cruz	1:20
		Anti-collagen 1(COL1A1)	Rabbit	HPA-011795, Sigma Aldrich	1:50
Confocal Microscopy	Infiltration in 3D scaffolds	APC-anti-MUC-1	Mouse	355608, BioLegend	5 µl/sample
		BV605-anti-CD90	Mouse	328128, BioLegend	5 µl/sample
		FITC-anti-CD45	Mouse	304038, BioLegend	5 µl/sample
	Infiltration in dECM	Invitrogen™ DiD	N/A	D307, Invitrogen	1:1000
		Invitrogen™ DiI	N/A	D282, Invitrogen	1:1000
		Invitrogen™ DiO	N/A	D3898, Invitrogen	1:1000
	Infiltration in tumors 3D scaffolds	Anti-cytokeratin	Mouse	NBP2-29429, NOVUS	5 µl/sample
		PE-anti-hFAP	Mouse	FAB3715P-100 R&D system	5 µl/sample
		FITC-anti-CD45	Mouse	304038, Biolegend	5 µl/sample
		APC-Goat-anti-mouse IgG	Goat	405308, Biolegend	1 µl/sample
	Multiplex Immunofluorescence in HGSC tissues	Anti-cytokeratin, cy5	Mouse	NBP2-29429, NOVUS	1:50
		Anti-vimentin, Red 610		MA5-11883, Invitrogen	1:50
		FITC-anti-CD45	Mouse	304038, Biolegend	1:100
Western blot	Canonical signaling	anti-TGF-β (56E4)	Rabbit	3709S, Cell Signaling	1:1000
		anti-SMAD 2/3 (D7G7)	Rabbit	8685, Cell Signaling	1:1000
		anti-phospho-SMAD2 (Ser	Rabbit	8828, Cell signaling	1:1000

		465/467)/ SMAD3 (Ser423/425)			
		anti-SMAD4	Rabbit	38454, Cell Signaling	1:1000
	Non-canonical signaling	anti-Akt (pan) (C67E7)	Rabbit	4691S, Cell Signaling	1:1000
		anti-phospho-Akt (Ser473) (D9E)	Rabbit	4060S, Cell Signaling	1:1000
		anti-p44/42 MAPK (Erk1/2) (137F5)	Rabbit	4695S, Cell Signaling	1:1000
		anti-phospho-p44/42 MAPK (Erk1/2) (Thr202/Tyr204) (D13.14.4E)	Rabbit	4370S, Cell Signaling	1:1000
		Anti-β-actin (13E5)	Rabbit	4970S, Cell Signaling	1:1000
		Anti-rabbit IgG, HRP-linked secondary	Goat	7074, Cell Signaling	1:3000
	Metalloproteinase-9	Anti-MMP-9	Rabbit	MA5-32705, ThermoScientific	1:1000
Imaging flow cytometry	TGF-β	Anti-TGF-β (3C11)	Mouse	sc130348, Santa Cruz	5 µl/sample
		CF™ 488A anti-mouse IgG (H+L) secondary antibody	Goat	SAB4600042, Sigma-Aldrich	0.5 µl/sample
	Collagen I Both assays	AF488-anti-collagen I	Rabbit	ab275996, Abcam	1 µl/sample
		APC-anti-FAP	Mouse	FAB3715A, R&D Systems	1 µl/sample
Flow cytometry	HIF-1a	AF647-anti-HIF-1α antibody	Mouse	359706, BioLegend	5 µl/sample
	Immune Functional Assays	AF700-anti-CD3	Mouse	300424, BioLegend	1 µl/sample
		FITC-anti-CD4	Mouse	300538, BioLegend	1 µl/sample
		Perc/Cy5.5-anti-CD8	Mouse	344710, BioLegend	1 µl/sample
		AF647-anti-FOXP3	Mouse	320114, BioLegend	1 µl/sample
		APC-anti-CD69	Mouse	310910, BioLegend	1 µl/sample
		BV605-anti-CD25	Mouse	356142, BioLegend	1 µl/sample

		BV421-anti-IFNγ	Mouse	502532, BioLegend	1 µl/sample
		PE/Dazzle™ 594 anti-IL-2	Rat	500344, BioLegend	1 µl/sample
		BV421-anti-CD223 (LAG3)	Mouse	369314, BioLegend	1 µl/sample
		PE/Dazzle 594™-anti-PD-1	Mouse	329940, BioLegend	1 µl/sample
		PE-Cy7-anti-granzyme B	Mouse	372214, BioLegend	1 µl/sample
	Immunophenotyping	BV421-anti-CD276 (B7-H3)	Mouse	565829, BD Horizon	1 µl/sample
		PE-anti-FAP	Mouse	FAB3715P, R&D Systems	1 µl/sample
		BV711-anti-CD3	Mouse	300464, BioLegend	1 µl/sample
		PE-Cy5-anti-CD4	Mouse	317412, BioLegend	1 µl/sample
		APC-Cy7-anti-CD8	Mouse	344714, BioLegend	1 µl/sample
		BV785-anti-CD14	Mouse	301840, BioLegend	1 µl/sample
		APC-anti-CD19	Mouse	302212, BioLegend	1 µl/sample
		PE-Cy7-anti-CD56	Mouse	318318, BioLegend	1 µl/sample
		FITC-anti-CD11b	Mouse	301330, BioLegend	1 µl/sample
	Apoptosis Detection kit with PI	FITC Annexin V Apoptosis detection kit with PI	N/A	640914, BioLegend	5 µl/sample
		BV510-anti-CD45	Mouse	304036, BioLegend	1 µl/sample
	Cytotoxic effect in 2D cultures	Invitrogen™ DiO	N/A	D3898, Invitrogen	1:1000
		PerCP/Cy5.5-anti-CD8	Mouse	344710, BioLegend	1 µl/sample
		PE-Cy7-anti-granzyme B	Mouse	372214, BioLegend	1 µl/sample
Cytokine array	Pro-tumoral Cytokines	IL-1β, IL-6, IL-8, macrophage inflammatory protein 1β (MIP-1β), granulocyte colony stimulating factor (G-CSF), granulocyte-macrophage	N/A	PPX45MXGZF4J, Thermo Scientific	N/A

		colony stimulating factor (GM-CSF), vascular endothelial growth factor A (VEGF-A)			
	Anti-tumoral Cytokines	IL-2, IL-7, IL-17A, interferon γ (IFNγ), tumor necrosis factor alpha (TNFα)	N/A	PPX45MXGZF4J, Thermo Scientific	N/A
Chemokine Array	Immune attractant chemokines	CCL2/JE/MCP-1<br>MIF<br>CXCL10/IP-10/CRG-2	N/A	LXSAHM-08<br>R&D Systems	N/A
