# Supplementary material for: Patient-derived model capturing hypoxia and extracellular matrix remodelling of immunologically cold high-grade serous tumours
Source: Nat Commun. 2026 Jul 11;17:8568. doi: 10.1038/s41467-026-75262-8 (PMC13482822; doi:10.1038/s41467-026-75262-8)
Supplement: Supplementary file 2 [file 41467_2026_75262_MOESM2_ESM.pdf]

Description of Additional Supplementary Files

Supplementary Data 1. Differential gene expression dataset.
