# Supplementary material for: Patient-derived model capturing hypoxia and extracellular matrix remodelling of immunologically cold high-grade serous tumours
Source: Nat Commun. 2026 Jul 11;17:8568. doi: 10.1038/s41467-026-75262-8 (PMC13482822; doi:10.1038/s41467-026-75262-8)
Supplement: Supplementary file 3 [file 41467_2026_75262_MOESM4_ESM.pdf]

nature portfolio

Corresponding author(s): Pilar de la Puente

Last updated by author(s): Apr 10, 2026

Reporting Summary

Nature Portfolio wishes to improve the reproducibility of the work that we publish. This form provides structure for consistency and transparency in reporting. For further information on Nature Portfolio policies, see our Editorial Policies and the Editorial Policy Checklist.

Statistics

For all statistical analyses, confirm that the following items are present in the figure legend, table legend, main text, or Methods section.

n/a	Confirmed	
☐	☒	The exact sample size (*n*) for each experimental group/condition, given as a discrete number and unit of measurement
☐	☒	A statement on whether measurements were taken from distinct samples or whether the same sample was measured repeatedly
☐	☒	The statistical test(s) used AND whether they are one- or two-sided<br>*Only common tests should be described solely by name; describe more complex techniques in the Methods section.*
☒	☐	A description of all covariates tested
☐	☒	A description of any assumptions or corrections, such as tests of normality and adjustment for multiple comparisons
☐	☒	A full description of the statistical parameters including central tendency (e.g. means) or other basic estimates (e.g. regression coefficient) AND variation (e.g. standard deviation) or associated estimates of uncertainty (e.g. confidence intervals)
☐	☒	For null hypothesis testing, the test statistic (e.g. *F*, *t*, *r*) with confidence intervals, effect sizes, degrees of freedom and *P* value noted<br>*Give P values as exact values whenever suitable.*
☒	☐	For Bayesian analysis, information on the choice of priors and Markov chain Monte Carlo settings
☒	☐	For hierarchical and complex designs, identification of the appropriate level for tests and full reporting of outcomes
☐	☒	Estimates of effect sizes (e.g. Cohen's *d*, Pearson's *r*), indicating how they were calculated

Our web collection on statistics for biologists contains articles on many of the points above.

Software and code

Policy information about availability of computer code

Data collection	Aperio ImageScope x64 software was used to collect immunohistochemistry images. NIS-Elements AR Analysis 5.21.03 Software was used for collection and analysis of immunofluorescent images. IDEAS v6.2 software was used to collect and analyze imaging flow cytometer data. Image Lab Software was used for the collection and processing of western blot images. FACSDiva v6.1.2 software was used for the collection of flow cytometry data. Belysa® Immunoassay Curve Fitting Software was used for luminex immunoassay collection data.
Data analysis	This study used the open-source R packages Seurat (https://github.com/satijalab/seurat) and CellChat (https://github.com/jinworks/CellChat) for quality control, analysis, and visualization of single-cell RNA-seq data and cell-cell communication. For bulk RNA-seq analysis ssGSEA (https://rpubs.com/snijesh/ssgsea-analysis-explained) and GSVA (https://www.bioconductor.org/packages/release/bioc/vignettes/GSVA/inst/doc/GSVA.html) analysis were performed in R. Publicly available gene set collections were downloaded from GSEA database for HALLMARK_HYPOXIA (https://www.gsea-msigdb.org/gsea/msigdb/human/geneset/HALLMARK_HYPOXIA.html), NABA_MATRISOME (https://www.gsea-msigdb.org/gsea/msigdb/cards/NABA_MATRISOME) and HALLMARK_TGF_BETA_SIGNALING (https://www.gsea-msigdb.org/gsea/msigdb/human/geneset/HALLMARK_TGF_BETA_SIGNALING.html). Image J plugins were used to analyze second harmonic generation iand picrosirius red images. FlowJo_v10.8.1_CL software was used to analyze all flow cytometry data. GraphPad Prism software version 9 and 10 was used to present the data and the statistical analysis

For manuscripts utilizing custom algorithms or software that are central to the research but not yet described in published literature, software must be made available to editors and reviewers. We strongly encourage code deposition in a community repository (e.g. GitHub). See the Nature Portfolio guidelines for submitting code & software for further information.

Data

Policy information about availability of data

All manuscripts must include a data availability statement. This statement should provide the following information, where applicable:

- Accession codes, unique identifiers, or web links for publicly available datasets
- A description of any restrictions on data availability
- For clinical datasets or third party data, please ensure that the statement adheres to our policy

The bulk RNA sequencing data are submitted to GEO database (GSE327288). The single-cell RNA sequencing data are submitted to GEO database (GSE299200).

Research involving human participants, their data, or biological material

Policy information about studies with human participants or human data. See also policy information about sex, gender (identity/presentation), and sexual orientation and race, ethnicity and racism.

Reporting on sex and gender	The findings in this study apply only to female sex. Only female samples were considered, as ovarian cancer affects only female reproductive tract
Reporting on race, ethnicity, or other socially relevant groupings	Tissue samples used in this study were from White/Caucasian Non-Hispanic women. No race-/ ethnicity- or other socially relevant grouping based analyses was performed in this study.
Population characteristics	The covariate-relevant population characteristics from the ovarian cancer patients that tissue/blood was collected are disclosed in the Table 1, including age, diagnosis, and treatment history.
Recruitment	Tumor biopsy and peripheral blood samples from ovarian cancer patients undergoing a cytoreductive surgery were provided by the Sanford USD Medical Center, Sioux Falls, SD, or the University of Iowa, Iowa City, IA.
Ethics oversight	Informed consent was signed by all subjects with approval from the Sanford Health Institutional Review Board (STUDY00001315) or University of Iowa's Institutional Review Board (#201809807), respectively and in accordance with the Declaration of Helsinki.

Note that full information on the approval of the study protocol must also be provided in the manuscript.

Field-specific reporting

Please select the one below that is the best fit for your research. If you are not sure, read the appropriate sections before making your selection.

☒ Life sciences ☐ Behavioural & social sciences ☐ Ecological, evolutionary & environmental sciences

For a reference copy of the document with all sections, see nature.com/documents/nr-reporting-summary-flat.pdf

Life sciences study design

All studies must disclose on these points even when the disclosure is negative.

Sample size	The sample size with primary samples was determined to achieve power of 0.8 with an effect size of 0.5 with a type I error of 0.05 for a two-way ANOVA design.
Data exclusions	Outliers cells in the MFI for TGF-b and collagen 1 signaling detected by imaging flow cytometry were excluded from the study using the ROUT (Q=10%) method in GraphPad Prism.
Replication	Experiments were performed in triplicates and repeated at least three times in cell lines or at least six independent primary cancer patient samples were used.
Randomization	The samples were randomly assigned for each assay based on avalaibility
Blinding	The data collections and data analysis was blinded without knowing specific characteristics in terms of treatment, stage or age of the patients that could confound the results.

Reporting for specific materials, systems and methods

We require information from authors about some types of materials, experimental systems and methods used in many studies. Here, indicate whether each material, system or method listed is relevant to your study. If you are not sure if a list item applies to your research, read the appropriate section before selecting a response.

Materials & experimental systems

n/a	Involved in the study
☐	☒ Antibodies
☐	☒ Eukaryotic cell lines
☒	☐ Palaeontology and archaeology
☒	☐ Animals and other organisms
☒	☐ Clinical data
☒	☐ Dual use research of concern
☒	☐ Plants

Methods

n/a	Involved in the study
☒	☐ ChIP-seq
☐	☒ Flow cytometry
☒	☐ MRI-based neuroimaging

Antibodies

Antibodies used

The following antibodies were used in the disclosed techniques: 1) In IHC staining: anti-HIF1α (Rabbit, HPA001275, Sigma Aldrich), anti-collagen I (Rabbit, HPA011795, Sigma Aldrich), anti-α-SMA (Mouse NBP2-33006, Novus), anti-fibronectin (Rabbit, ab 2413, Abcam), anti-CD8 (Rabbit SP16, Cell Marque), anti-TGF-β 3C11 (Mouse, sc 130348, Santa Cruz). 2) For immunofluorescent staining: APC-anti-MUC-1 (Mouse, 355608, BioLegend), BV605-anti-CD90 (Thy1) (Mouse, 328128, BioLegend), FITC-anti-CD45 clone HI30 (Mouse, 304038, BioLegend), Anti-PAN-cytokeratin (AE-1/AE-3) (Mouse, NBP2-29429, NOVUS), PE-anti-hFAP (Mouse, FAB3715P-100, R&D system), FITC-anti-CD45 (Mouse, 304038, Biolegend), APC-Goat-anti-mouse IgG (Goat 405308, Biolegend), Anti-vimentin (V9) (MA5-11883, Invitrogen). 3) For Western blot: anti-TGF-β (56E4) (Rabbit, 3709S, Cell Signaling), anti-SMAD 2/3 (D7G7) (Rabbit, 8685, Cell Signaling), anti-phospho-SMAD2 (Ser 465/467)/ SMAD3 (Ser423/425) (Rabbit, 8828, Cell signaling), anti-SMAD4 (Rabbit, 38454, Cell Signaling), anti-Akt (pan) (C67E7) (Rabbit, 4691S, Cell Signaling), anti-phospho-Akt (Ser473) (D9E) (Rabbit, 4060S, Cell Signaling), anti-p44/42 MAPK (Erk1/2) (137F5) (Rabbit, 4695S, Cell Signaling), anti-phospho-p44/42 MAPK (Erk1/2) (Thr202/Tyr204) (D13.14.4E) (Rabbit, 4370S, Cell Signaling), Anti-β-actin (13E5) (Rabbit, 4970S, Cell Signaling), Anti-rabbit IgG, HRP-linked secondary (Goat, 7074, Cell Signaling), Anti-MMP-9 (JA80-73, Rabbit, MA5-32705, ThermoScientific). 4) For imaging flow cytometry: anti-TGF-β (3C11) (Mouse, , sc130348, Santa Cruz), CF 488A anti-mouse IgG (H+L) secondary antibody (Goat, SAB4600042, Sigma-Aldrich), AF488-anti-collagen I [EPR7785] (Rabbit, ab275996, Abcam), APC-anti-FAP (AF3715, Mouse, FAB3715A, R&D Systems). 5) For flow cytometry: AF647-anti-HIF-1α clone 546-16 (Mouse, 359706, BioLegend), AF700-anti-CD3 clone UCHT1 (Mouse, 300424, BioLegend) FITC-anti-CD4 clone RPA-T4 (Mouse, 300538, BioLegend), Perc/Cy5.5-anti-CD8 clone SK1 (Mouse, 344710, BioLegend), AF647-anti-FOXP3 clone 206D(Mouse, 320114, BioLegend), APC-anti-CD69 clone FN50 (Mouse, 310910, BioLegend), BV605-anti-CD25 clone M-A251 (Mouse, 356142, BioLegend), BV421-anti-IFNγ clone AS.B3 (Mouse, 502532, BioLegend), PE/Dazzle 594 anti-IL-2 clone MQ1-17H12 (Rat, 500344, BioLegend), BV421-anti-CD223 (LAG3) clone 11C3C65 (Mouse, 369314, BioLegend), PE/Dazzle 594-anti-PD-1 clone EH12.2H7 (Mouse, 329940, BioLegend), PE-Cy7-anti-granzyme B clone QA16A02 (Mouse, 372214, BioLegend), BV421-anti-CD276 (B7-H3) clone 7-517 (Mouse 565829, BD Horizon), PE-anti-hFAP (Mouse, FAB3715P, R&D Systems), BV711-anti-CD3 clone UCHT1 (Mouse, 300464, BioLegend), PE-Cy5-anti-CD4 clone OKT4 (Mouse,317412, BioLegend), APC-Cy7-anti-CD8 clone SK1 (Mouse, 344714, BioLegend), BV785-anti-CD14 clone M5E2 (Mouse, 301840, BioLegend), APC-anti-CD19 clone HIB19 (Mouse, 302212, BioLegend), PE-Cy7-anti-CD56 clone HCD56 (Mouse, 318318, BioLegend), FITC-anti-CD11b clone ICRF44 (Mouse, 301330, BioLegend). 6) For apoptosis & cytotoxicity assays: BV510-anti-CD45 clone HI30 (Mouse, 304036, BioLegend), PerCP/Cy5.5-anti-CD8 clone SK1(Mouse, 344710, BioLegend), PE-Cy7-anti-granzyme B clone QA16A02 (Mouse, 372214, BioLegend).

Validation

All antibodies were validated for their use in the application and species according to manufacturer's websites.

Eukaryotic cell lines

Policy information about cell lines and Sex and Gender in Research

Cell line source(s)

KURAMOCHI (RRID:CVCL1345), OVSAHO (RRID:CVCL_3114), OVCAR-3 (ATCC HTB-161), OVCAR-8 (RRID:CVCL_1629), CAOV3 (ATCC HTB-75), OV81.2 and HEYA8 (RRID:CVCL_8878)

Authentication

All cell lines are routinely profiled by ATCC

Mycoplasma contamination

Cells were tested negative for mycoplasma

Commonly misidentified lines
(See ICLAC register)

N/A

Plants

Seed stocks

Report on the source of all seed stocks or other plant material used. If applicable, state the seed stock centre and catalogue number. If plant specimens were collected from the field, describe the collection location, date and sampling procedures.

Novel plant genotypes

Describe the methods by which all novel plant genotypes were produced. This includes those generated by transgenic approaches, gene editing, chemical/radiation-based mutagenesis and hybridization. For transgenic lines, describe the transformation method, the number of independent lines analyzed and the generation upon which experiments were performed. For gene-edited lines, describe the editor used, the endogenous sequence targeted for editing, the targeting guide RNA sequence (if applicable) and how the editor was applied.

Authentication

Describe any authentication procedures for each seed stock used or novel genotype generated. Describe any experiments used to assess the effect of a mutation and, where applicable, how potential secondary effects (e.g. second site T-DNA insertions, mosiacism, off-target gene editing) were examined.

Flow Cytometry

Plots

Confirm that:

☒ The axis labels state the marker and fluorochrome used (e.g. CD4-FITC).

☒ The axis scales are clearly visible. Include numbers along axes only for bottom left plot of group (a 'group' is an analysis of identical markers).

☒ All plots are contour plots with outliers or pseudocolor plots.

☒ A numerical value for number of cells or percentage (with statistics) is provided.

Methodology

Sample preparation

The 3D scaffolds were digested on the day of analysis and the isolated cells, unless otherwise stated, were stained with LIVE/DEAD Fixable blue dead cell stain kit (L23105, Invitrogen), fixed and permeabilized with the Cyto-FastTM Fix/Perm Buffer Kit (426803, BioLegend) to detect intracellular markers, blocked with the Human TruStain FcXTM (422302, BioLegend) and stained with specific antibodies disclosed in (Table 2). To detect HIF-1α expression and intracellular cytokines or activation and exhaustion surface markers in patient-derived cultures, primary HGSC cells were co-cultured with matching PBMCs for 2 days inside the PLR or HHR generated with autologous plasma. The PBMCs were pre-stimulated for 5 hours for immune functional characterization. In the immunophenotyping studies, not fixed cells were stained to detect cancer cells (B7H3+), CAFs (FAP+), CD3+, CD4+, CD8+ T lymphocytes, macrophages (CD14+), B cells (CD19+), NK cells (CD56+), and myeloid-derived suppressor cells (MDSC, CD11b+). To explore the cytotoxic effect of PBMCs on HGSC, KURAMOCHI cells were mono-cultured or co-cultured inside the PLR and HHR with stimulated PBMCs for 5 days and not fixed cells were stained with antibodies to detect PBMCs (CD45+) and KURAMOCHI (CD45-) and then with propidium iodide (PI) and Annexin V in Annexin V Binding Buffer kit (422201, BioLegend) to detect live, early apoptotic and necrotic cells. Alternatively, the cytotoxic effect of the immune cells on HGSC was evaluated in 2D cultures of KURAMOCHI cells stained with cell surface marker DiO (green) co-cultured with PBMCs at increasing ratios (1:1, 1:2, 1:3 and 1:4) in normoxia (21% O2) or hypoxia (1.5% O2) for 2 days.

Instrument

BD FACS Fortessa serial number H64717700065

Software

FACSDiva v6.1.2 software was used to collect the data and FlowJo v10 software was used to analyze the data

Cell population abundance

Minimum of a ten thousand Precision Count BeadsTM per sample was acquired.

Gating strategy

For immune functional assays and immunophenotyping studies, single live cells were analyzed by dimensionality reduction analysis using Phenograph v2.5.0 and UMAP v4.1.1. (default settings) plugins in the FlowJo software. The level of expression of the markers in functional assays was determined as the frequency (percentage of the CD4+ or CD8+ parent population) and the stacked charts expressed the percentage of each subpopulation in the total population of CD4+ or CD8+ T cells, with a total of 100%. The different cluster populations of infiltrated lymphocytes were identified with different colors and numbers and expressed as counts of cells positive for the selected marker. inside the 3D scaffold. The cytotoxic effect of PBMCs on KURAMOCHI cells in the 3D scaffolds was expressed as a percentage of KURAMOCHI cells in each stage (live, early apoptosis and necrosis), and the cytotoxic effect in 2D was determined as the fold change of live KURAMOCHI cells in the absence of PBMCs. For HIF-1a expression analysis, the cancer cells vs lymphocytes were visualized based on size using SSC vs FSC , then single cells, live cells, then gated CD3+ in lymphocytes and CD44+ HGSC cells and show histograms for HIF1a.

☒ Tick this box to confirm that a figure exemplifying the gating strategy is provided in the Supplementary Information.
