# Supplementary material for: Patient-derived model capturing hypoxia and extracellular matrix remodelling of immunologically cold high-grade serous tumours
Source: Nat Commun. 2026 Jul 11;17:8568. doi: 10.1038/s41467-026-75262-8 (PMC13482822; doi:10.1038/s41467-026-75262-8)
Supplement: Supplementary file 4 [file 41467_2026_75262_MOESM5_ESM.pdf]

nature portfolio

Peer Review File

Patient-derived model capturing hypoxia and extracellular matrix remodelling of immunologically cold high-grade serous tumours

Corresponding Author: Dr Pilar de la Puente

This file contains all reviewer reports in order by version, followed by all author rebuttals in order by version.

Version 0:

Reviewer comments:

Reviewer #1

(Remarks to the Author)
In their work "Mimicking physiologically relevant environments in patient-derived tumor-immune tunable models 1 to target immunologically cold high-grade serous tumors", Plesselova et al present an in vitro tumor model comprising tumor and immune cells in the context of hypoxia and ECM remodeling, both features characteristic of HGSCs with poor clinical outcome.

Overall, the work contributes to the field of hypoxia in immunologically cold tumors and the paper follows a linear logical path. However, specific point should be addressed, in order for this work to be fully interpretable and publishable:

Major comments:
- It seems that normal plasma was utilized to generate nECMPhys models, while plasma from cancer patients was used for tECMHyp. These two models are then kept at physician or hypoxia, respectively. However, at no point in the study do authors check the tumor plasma in physioxia or the normal plasma in hypoxia; therefore there is a major confounding factor present in this study. Please address this point.
- It is unclear what the authors mean by "tunable", as the work presented speaks about 2 distinct states (hypoxia vs normoxia), rather than oxygen levels that can be tuned/changed for example within a given experiment. Please elaborate.
- In several experiments, either HGSC cells from a cell line or from primary tissue are used; however it seems that whenever primary HGSC cells are used, the entire tissue digest is utilized, implying that numerous cell types present in the digest are applied to the model in this case. How do authors account for heterogeneity in the digest and what was the amount of tumor cells present in the digests?
- Fig 1: it is unclear whether scRNA-Seq data obtained from the in vitro models was compared head-to-head to tumors/tissues of origin and if not, why this was not done as a model benchmarking step. Please elaborate.
- Fig 1a: while in the text it was implied that normal ovaries have higher physiological oxygen levels than HGSC tumors, it appears from this figure that in fact both the nECMPhys and the tECMHypo models comprise tumor cells and CAFs - in other words, the non-tumor ovary was not modelled in this work. Please revise the text to clearly reflect this for clarity; specifically, please explain that "nECMPhys" is not a model of the normal ovary, or, if it is, please address in what way it is "normal", given that tumor cells and CAFs seem to have been used for both models.
- Fig 2: It is unclear why figure 2, which contains crucial details on the model development, is introduced after figure 1. Would it not make more sense to swap the order of these figures so as to linearly present the model characteristics and then show these lead to the phenotypes assessed in the scRNA-Seq? Please elaborate.
- Fig 2b: Do patient-derived tumor cells also show hypoxia by this fluorescent marker?
- Fig 2: for co/culture experiments with CAFs, were these primary CAFs sourced from digest? If not, please elaborate on the source and characteristics of these CAFs.
- Fig 3: infiltration of various immune cells was observed to be decreased in CAF co-cultures in hypoxia, compared to normoxia and authors conclude that this was mediated by a higher amount of ECM remodeling in hypoxic conditions. It would be interesting to make a distinction between this conclusion and the alternative, which is that in hypoxic conditions, co-culture cells do not secrete as many chemo/cytokines to attract immune cells, by performing a cytokine analysis on the supernatants in hypoxic vs normoxic co-cultures prior to adding PBMCs.
- Fig 4: Here the authors observed that treatment with galunisertib decreased collagen expression in hypoxic co-cultures but

increased it in normoxic co-cultures. This is surprising given that galunisertib is widely used to inhibit TGFb-mediated phenotypes across a number of models and biological settings. Please elaborate further the interpretation of these data.

Discussion: a discussion is needed on the confounding variable that plasma from cancer patients seems to only have been used only in the hypoxic model, despite conclusions being made on immune cell infiltration and ECM deposition being differentially observed in the hypoxic condition, compared to the normoxic condition. Conversely, if authors did indeed utilize plasma for normoxic and hypoxic conditions, this requires a much clearer explanation of which was used for what experiments and a discussion of what differences were found between these plasma sources which could confound the conclusions drawn.

Minor comments:
- In the abstract, the sentence “the models integrate cancer cells co-cultures with CAFs…” is too complicated and confusing. Please revise for clarity.
- Fig 1a is further confusing, as it seems to imply that ECM was added to the tumor model, but not to the normal model. Perhaps the authors mean that ECM is eventually deposited at higher levels in the tumor model? Please revise for clarity.

Reviewer #2

(Remarks to the Author)
This manuscript bioengineers patient-derived 3D tumor–stroma–immune models under differential oxygen tensions to interrogate CAF-driven ECM remodeling and immune infiltration, and tests TGF-βR1 inhibition (galunisertib) as a putative “rescue”. The topic is important and the platform is promising. However, multiple conceptual, methodological, and statistical issues substantially limit interpretability and translational relevance.
Major comments:
1. The authors present scRNA-seq/flow data for cellular composition but do not visually validate the 3D spatial architecture. Without multiplex immunofluorescence/immunohistochemistry (mIF/mIHC) or spatial imaging, it is impossible to ascertain whether the model reconstructs spatial patterns relevant to immune exclusion.
2. Panels 2h–i (T-cell functional phenotypes) are thematically disconnected from 2a–g (oxygen/ECM mechanics) and align more naturally with the immune-infiltration narrative in Figure 3, which complicates readability and causal logic.
3. Western blots in figure 4b–c appear overexposed and trends are not convincingly quantified. In Figure 4d (left panel), TGF-β in CAFs under tECMHyp appears lower than under nECMPhys, conflicting with Figure 4c where pathway activity is higher in hypoxia. Moreover, galunisertib targets TGF-βR1 signaling, not ligand production as shown in figure 4a, so why does intracellular TGF-β protein decrease after galunisertib?
4. In figure5, paired t-tests are applied to comparisons that do not reflect true within-donor pairing. The validity of key significances is uncertain; a correct framework (unpaired or mixed-effects with donor as a random factor and multiplicity control) may materially alter the conclusions.
5. Figure 6 appears to reconstruct a model distinct from earlier figures. The motivation, differences, and added value relative to the primary platform are not explained.
6. Discovery and “validation” both occur in vitro. There is no confirmation in human tumor tissues that TGF-β/CAF/COL1 axes and the proposed rescue signatures track with immune infiltration in patients. Please include orthogonal validation in clinical material linking CAF/TGF-β/COL1 to immune exclusion.

Minor comments:
1. The authors describe the result in the multi-culture as having “lower expression of collagen I” in line 197. This phrasing is problematic. I think “expression” is biologically imprecise for a deposited extracellular protein, maybe “level” or “deposition” would be more accurate.
2. The title of Figure 3 asserts that hypoxia-induced, CAF-driven ECM remodeling “causes” impaired immune infiltration. Given the largely correlative design and limited perturbations that decouple biochemical cues from biomechanical barriers, the data support association rather than causation.
3. The y-axis range is excessively broad in Figure 4d (right panel), the range of which is excessively broad.
4. Figure 2d–g suggests lower collagen I under tECMHyp in some contexts, yet stiffness is higher. A mechanistic explanation is missing.

Version 1:

Reviewer comments:

Reviewer #1

(Remarks to the Author)
The authors have made substantial efforts to clarify points raised in the review of their work, as well as adding considerable amounts of analysis and suggested experiments to clarify the model interpretability. Minor remaining comments to address at this point are:
- image and plot axis labels should, where possible, be increased in size
- fluorescence intensities should be, where possible increased for image viewability

Aside from these, the authors have satisfied the criteria for publication.

Reviewer #2

(Remarks to the Author)
The revised manuscript has addressed most of my previous concerns. The added spatial imaging strengthens the evidence that the HHR model recapitulates impaired immune infiltration, and the revised statistical analyses are more appropriate. The additional HGSC tissue validation, although limited in sample size, provides supportive evidence for the association between collagen deposition and immune exclusion. I have no further major concerns. I only suggest that the authors further temper statements implying that galunisertib definitively converts immunologically cold tumors into hot tumors, as the current data primarily support a model-based and hypothesis-generating conclusion.

Open Access This Peer Review File is licensed under a Creative Commons Attribution 4.0 International License, which permits use, sharing, adaptation, distribution and reproduction in any medium or format, as long as you give appropriate credit to the original author(s) and the source, provide a link to the Creative Commons license, and indicate if changes were made.
In cases where reviewers are anonymous, credit should be given to 'Anonymous Referee' and the source.
The images or other third party material in this Peer Review File are included in the article's Creative Commons license, unless indicated otherwise in a credit line to the material. If material is not included in the article's Creative Commons license and your intended use is not permitted by statutory regulation or exceeds the permitted use, you will need to obtain permission directly from the copyright holder.
To view a copy of this license, visit https://creativecommons.org/licenses/by/4.0/

REVIEWER COMMENTS

Reviewer #1, cancer 3D models (Remarks to the Author):

In their work “Mimicking physiologically relevant environments in patient-derived tumor-immune tunable models to target immunologically cold high-grade serous tumors”, Plesselova et al present an in vitro tumor model comprising tumor and immune cells in the context of hypoxia and ECM remodeling, both features characteristic of HGSCs with poor clinical outcome. Overall, the work contributes to the field of hypoxia in immunologically cold tumors and the paper follows a linear logical path. However, specific point should be addressed, in order for this work to be fully interpretable and publishable:

We appreciate the reviewers’ comments that have helped us to improve the writing of the manuscript in a clearer way and incorporate additional studies that further strengthen the relevance of the study. Specifically, we have incorporated assays to functionally characterize our models, improve our understanding of its cellular and transcriptomic composition, as well as a cytokine array to decipher the mechanisms of impaired immune infiltrations in the hypoxic environment.

Major comments:

1. It seems that normal plasma was utilized to generate nECMPhys models, while plasma from cancer patients was used for tECMHyp. These two models are then kept at physician or hypoxia, respectively. However, at no point in the study do authors check the tumor plasma in physioxia or the normal plasma in hypoxia; therefore there is a major confounding factor present in this study. Please address this point.

We thank the reviewer for their valuable comment and apologize for any confusion regarding the description of the methods and results related to the generation of the 3D model. However, the interpretation underlying this concern is not correct, and therefore the proposed confounding factor is not present in our study.

When **established cancer HGSC cell lines** were used, we incorporated **healthy donor plasma and PBMCs**, rather than plasma or PBMCs from unrelated cancer patients. This choice was made for biological validity reasons. Specifically, combining cancer cell lines with allogeneic PBMCs or plasma from unrelated cancer patients would introduce uncontrolled immune incompatibility, including HLA mismatch and non–tumor-specific immune activation, which could dominate the observed effects and obscure ECM- and oxygen-dependent mechanisms.

When **patient-derived tumor samples** were used, these were combined with their **matched autologous plasma and PBMCs**, thereby preserving patient-specific immune–tumor interactions without introducing allogeneic artifacts.

Importantly, **in both experimental settings**, regardless of plasma or PBMC origin, the 3D models were subsequently exposed to **both physoxia and hypoxia.** Therefore, plasma origin and oxygen condition were independently controlled variables, and the study does not suffer from the confounding factor suggested by the reviewer.

We have clarified this rationale in the revised Methods and Results sections to prevent further ambiguity.

Following changes have been made in the manuscript:

Results: Pages 6-7, Lines 147-155: "Both PLR and HHR 3D models were generated under two experimental settings. In the patient-derived setting, dissociated primary HGSC tumor cells were cultured in the presence of autologous plasma and peripheral blood mononuclear cells (PBMCs), preserving patient-specific tumor-immune interactions. In parallel, immortalized HGSC cancer cell lines and cancer-associated fibroblasts (CAFs) were incorporated into 3D scaffolds generated with healthy donor–derived plasma and PBMCs, reflecting the lack of autologous material for established cell lines and providing a controlled immune background. Importantly, all models, regardless of plasma origin, were cultured under both physoxic and hypoxic conditions, allowing direct comparison of oxygen-dependent effects across healthy and cancer plasma contexts."

Methods: Page 34, Lines 844-846: "For HGSC patient-derived models, dissociated primary tumor tissues (10–25 mg) were embedded in 3D scaffolds generated using autologous, patient-matched plasma and PBMCs, thereby preserving patient-specific tumor-immune interactions."

Methods: Page 34-35, Line 848-852: "In contrast, for models constructed with immortalized cancer cell lines ($2x10^5$ cells/ml), plasma and PBMCs from healthy donors were used, as autologous immune components corresponding to the original tumor donors are not available for established cell lines and the use of unrelated cancer patient immune components would introduce uncontrolled allogeneic immune effects."

Methods: Page 35, Line 859-860: "The 3D scaffolds, regardless of plasma or PBMC source, were subsequently cultured under both physoxia and hypoxia conditions."

2. It is unclear what the authors mean by "tunable", as the work presented speaks about 2 distinct states (hypoxia vs normoxia), rather than oxygen levels that can be tuned/changed for example within a given experiment. Please elaborate.

The authors appreciate the reviewer's comment and agree with the suggestion. To avoid further confusion, the word 'tunable" has been removed from the manuscript, including the title, abstract, introduction, results, methods and discussion, and can be tracked in the tracked changes revised manuscript.

3. In several experiments, either HGSC cells from a cell line or from primary tissue are used; however it seems that whenever primary HGSC cells are used, the entire tissue digest is utilized, implying that numerous cell types present in the digest are applied to the model in this case. How do authors account for heterogeneity in the digest and what was the amount of tumor cells present in the digests?

We thank the reviewer for this important question regarding cellular heterogeneity in patient-derived HGSC models. When primary HGSC tissues were used, the entire dissociated tumor digest was intentionally incorporated into the 3D models, rather than isolating or normalizing specific cell populations. This design choice was made to preserve the intrinsic cellular heterogeneity of the tumor microenvironment, including malignant epithelial cells, stromal cells, and resident immune and endothelial populations, which is a defining feature of HGSC. We acknowledge that the exact proportion of tumor cells may vary between patient samples. However, this variability is biologically meaningful and reflects patient-to-patient differences. Normalizing or predefining tumor cell numbers across patient-derived samples would require extensive manipulation (e.g., cell sorting or selective expansion), which would disrupt native cell–cell interactions and compromise the physiological relevance of the model. To control for input material across patient-derived experiments, a defined tissue mass (10–25 mg) was used for all dissociations, and identical enzymatic and processing protocols were applied. This approach standardizes the overall cellular input while preserving sample-specific composition. Importantly, study endpoints were based on functional, phenotypic, and molecular outputs of the reconstructed tissue, rather

than assumptions about initial tumor cell fraction. These readouts capture the integrated behavior of the whole tumor microenvironment, which is the primary focus of this model. In parallel, we also included experiments using established cell lines, where the cellular composition and number were tightly controlled. This complementary design allowed us to distinguish tumor-intrinsic effects from those arising due to cellular heterogeneity in the tissue context. Ideally, as discussed in the future directions of this research, future studies could include other cell type populations in our 3D model to accurately represent the heterogenous tumor microenvironment.

We have clarified this rationale in the Methods section to better emphasize the intentional preservation of tumor heterogeneity in patient-derived models.

Methods: Page 34, Line 846-848: "Whole tumor digests were used without further cellular fractionation to preserve native tumor heterogeneity; cellular input was standardized by tissue mass and uniform dissociation protocols rather than by predefined tumor cell numbers."

4. - Fig 1: it is unclear whether scRNA-Seq data obtained from the in vitro models was compared head-to-head to tumors/tissues of origin and if not, why this was not done as a model benchmarking step.

In the current study, scRNA-seq profiling was performed on the in vitro 3D models shown in Fig. 2. Direct, head-to-head comparison with the corresponding tumors was not initially performed, as sufficient primary tumor material was not available to both generate the 3D models and independently process matched fresh tissue. Given this limitation, we prioritized single cell transcriptomic characterization of the engineered models themselves to ensure adequate depth and quality of sequencing. We agree with the reviewer that benchmarking against native tissue is an important validation step. To address this, we are now incorporating additional benchmarking analyses, including comparison of molecular subtype and important tumor microenvironmental pathways (hypoxia, matrisome and TGF-β signaling) fingerprints for similarities between HGSC tumor grown in our 3D models benchmarked to the tissue of origin detected by bulk RNA sequencing, which are included in the revised manuscript. These analyses are intended to assess the extent to which the engineered models recapitulate key cellular states and transcriptional programs observed in primary HGSC tumors.

We have clarified this point in the revised Methods section to explicitly state the scope and limitations of the initial scRNA-seq comparison. We have added the new single-sample Gene Set Enrichment Analysis (ssGSEA) of gene sets for molecular subtypes of HGSC defined by Tothill et al. (2008) and hypoxia, matrisome and TGF-β signaling pathways and the similarities calculated by Pearson's correlation between the original tumor and tumor grown in 3D models. Overall, we demonstrated that our models benchmark the original HGSC tissue and conserve these important molecular fingerprints.

Figures: 1 b,c,d,e,f, g and Supplementary Fig. 1 a-b

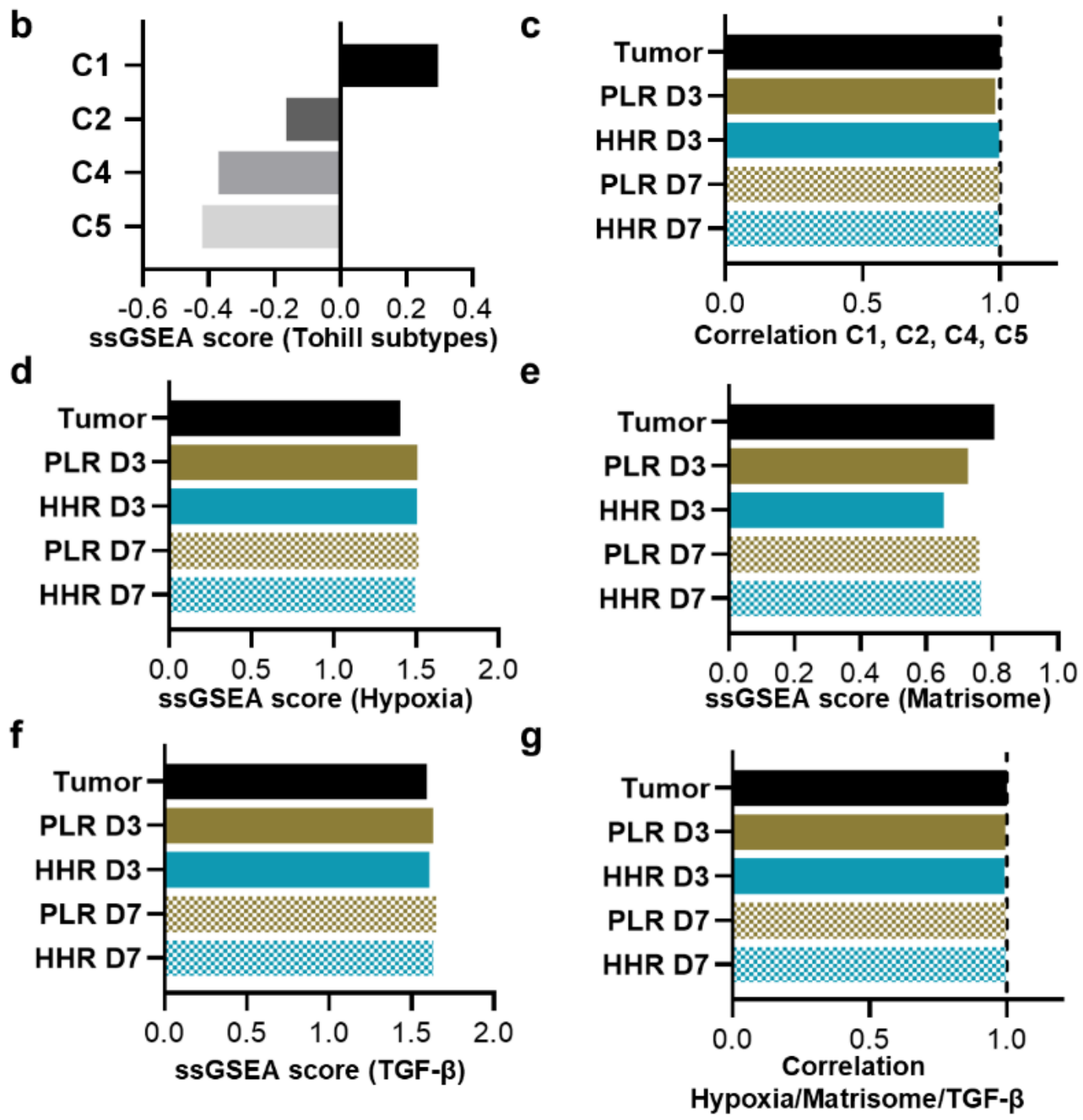

Fig. 1. Transcriptomic concordance and functional divergence across hypoxia and ECM remodeling in patient-derived 3D models. b Single-sample Gene Set Enrichment analysis (ssGSEA) scores for molecular subtypes of HGSC defined by Tothill et. al (2008): C1 (mesenchymal), C2 (immunoreactive), C4 (differentiated) and C5 (mesenchymal-like) in HGSC tumor detected by bulk RNA-seq. **c** Similarity of gene set expression for Tothill subtypes in fresh tumor and dissociated tumor grown in PLR or HHR for 3 (PLR&HHR D3) or 7 days (PLR&HHR D7) expressed by Pearson correlation. **d** ssGSEA scores for hypoxia, **e** matrisome, **f** TGF-β signaling gene sets enrichment and **g** similarity of gene enrichment for the three pathways between the tumor and tumor grown in the 3D models.

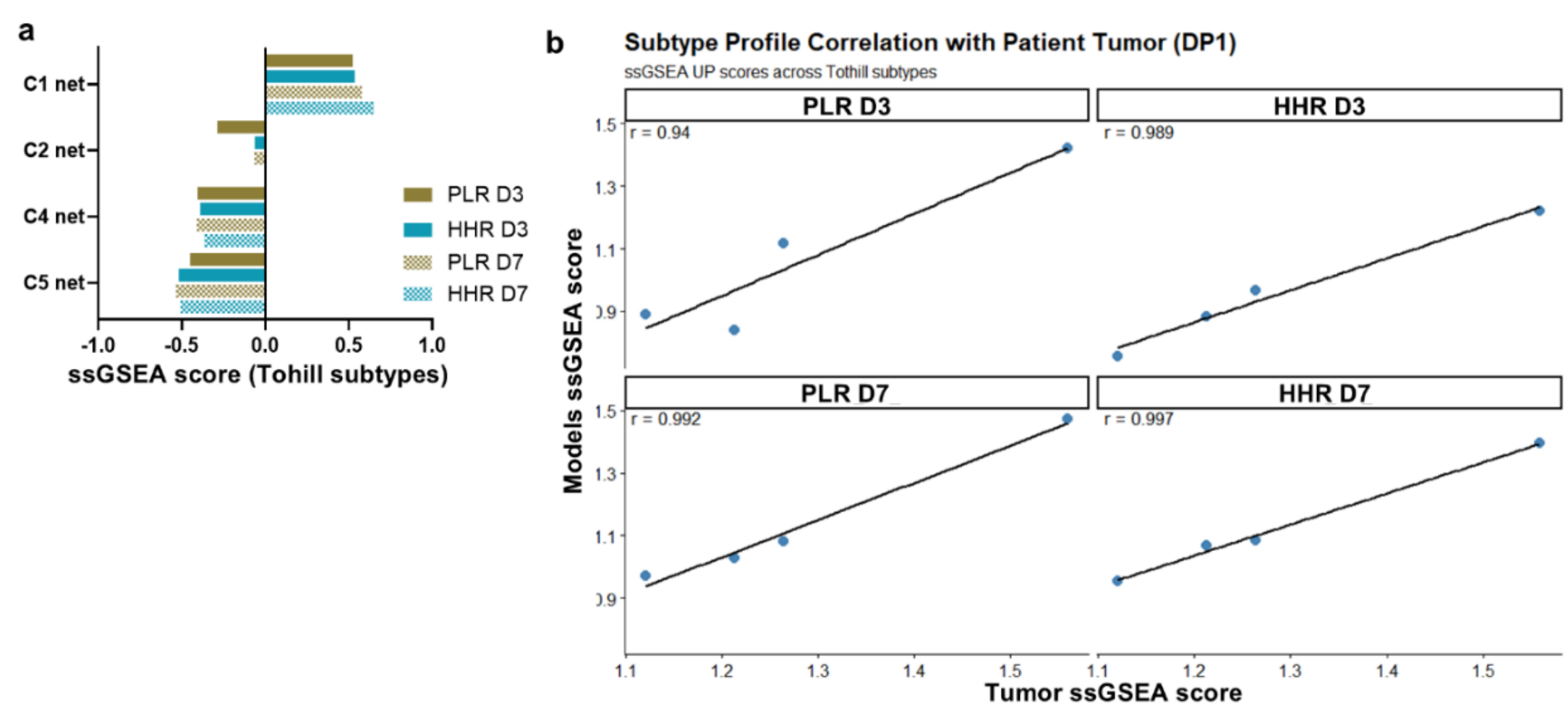

Supplementary Fig. 1. PLR and HHR models conserve the molecular fingerprints of HGSC tumor and recapitulate the physiologically relevant oxygen levels in early vs advanced HGSC. a Single-sample Gene Set Enrichment analysis (ssGSEA) of net scores for molecular subtypes of HGSC defined by Tothill et. al (2008): C1 (mesenchymal), C2 (immunoreactive), C4 (differentiated) and C5 (mesenchymal-like) in HGSC fresh tumor and the tumor grown in PLR or HHR for 3 days (PLR&HHR D3) or 7 days (PLR&HHR D7) detected by bulk RNA-seq. **b** Pearson correlation of gene scores for upregulated gene set across Tothill subtypes in 3D models (PLR and HHR, D3 and D7) correlated with fresh tumor.

Results: Page 7, Line 156-173: First, transcriptomic profiling of a dissociated HGSC tumor grown in PLR or HHR for 3 or 7 days was performed using bulk RNA sequencing, using fresh native HGSC tumor (not grown in 3D scaffolds) as a control for benchmarking analysis. Simple sample gene enrichment analysis (ssGSEA) revealed that both, the native HGSC tumor (Fig. 1b) and the tumor grown in the 3D models (Supplementary Fig. 1a), presented the highest net score for molecular HGSC subtype C1 (mesenchymal) compared to C2 (immunoreactive), C4 (differentiated) or C5 (mesenchymal-like) defined by Tothill[32]. All 3D patient models exhibited strong positive correlation (Pearson $r \geq 0.98$) with the native tumor, indicating that the overall pattern of subtype-specific transcriptional activity observed in the native tumor is highly conserved across the 3D models (Fig. 1c and Supplementary Fig. 1b). In addition, gene signatures for key microenvironmental features in the TME related to hypoxia, matrisome, and transforming growth factor-β (TGF-β) signaling were evaluated across all samples showing consistently high enrichment in both the native tumor and the 3D models. Hypoxia (Fig. 1d), matrisome (Fig. 1e), and TGF-β signaling (Fig. 1f) ssGSEA scores present in the patient tumor were broadly preserved in the PLR and HHR models at both time points. All 3D tumor models showed strong positive correlation with native tumor for these features (Pearson $r \geq 0.99$), indicating that hypoxia/matrisome/ TGF-β signaling transcriptomic profiles were conserved (Fig. 1g). Together, these findings demonstrate that the molecular fingerprints defining the HGSC subtype and key microenvironmental and signaling pathways are consistently maintained across the 3D tumor models compared to native tissue.

Discussion: Page 27, Line 656-659: "Here we demonstrated that the PLR and HHR 3D models benchmark the molecular fingerprints of the native tumor tissue by conserving the enriched gene signatures of the tumor molecular subtype and key microenvironmental features such as hypoxia, matrisome and transforming growth factor-β (TGF- β) signaling pathway."

Methods: Page 36-37, Line 898-899: "scRNA-seq profiling was performed on the engineered 3D models; matched primary tumor tissue from the same specimens was not available for parallel sequencing."

Methods: Pages 35-36, Lines 868-894: "**Bulk RNA sequencing** Primary cells isolated from dissociated HGSC stage III tumor were cultured in PLR or HHR for 3 or 7 days. Fresh tumor on day 0, not grown in the 3D model, was used as reference. Total RNA was extracted on respective days using Qiagen RNeasy Mini Kit following the manufacturer's instructions. Briefly, the tumor and the 3D scaffolds were lysed using RLT lysis buffer supplemented with β-mercaptoethanol and automatic tissue homogenizer. The RNA was eluted in nuclease-free water, and 400 ng were sent for sequencing with Illumina NovaSeq X Plus (Novogen). Messenger RNA was purified from total RNA using poly-T oligo-attached magnetic beads. After fragmentation, the first strand cDNA was synthesized using random hexamer primers followed by the second strand cDNA synthesis. The library was prepared after end repair, A-tailing, adapter ligation, size selection, amplification, and purification. Libraries were quantified using a Qubit fluorometer, with size distribution monitored on a representative subset of samples via the Agilent 5400 Fragment Analyzer. For Illumina sequencing, libraries were pooled based on the required data amount and effective concentration.

Raw gene-level count data were imported into DESeq2 (v1.44.0) in R software. Library size normalization was performed via size-factor estimation, followed by variance stabilization using the DESeq2 variance stabilizing transformation (VST) with design awareness (blind = FALSE).

To analyze gene enrichment scores for each individual sample reflecting the within-sample biology, Single-sample Gene Set Enrichment Analysis (ssGSEA) was performed in GSVA (v1.52.3) on the variance-stabilized expression matrix as input and gene sets defining the molecular subtypes of HGSC, namely C1 (mesenchymal), C2 (immunoreactive), C4 (differentiated) and C5 (mesenchymal-like)[32] . In addition, gene enrichment scores for hypoxia signatures, matrisome and TGF-β signaling were assessed using the publicly available HALLMARK_HYPOXIA, NABA_MATRISOME and HALLMARK_TGF_BETA-signaling gene set collections (GSEA database). Similarity among the native

HGSC tissue and tumor grown in 3D model was assessed comparing the ssGSEA enrichment profiles between each sample and the native tissue using Pearson correlation. For each sample, a vector of ssGSEA scores across C1 subtype or hypoxia/TGF-β signaling/ NABA Matrisome pathways were correlated with the corresponding vector from native tissue."

5. - Fig 1a: while in the text it was implied that normal ovaries have higher physiological oxygen levels than HGSC tumors, it appears from this figure that in fact both the nECMPhys and the tECMHypo models comprise tumor cells and CAFs - in other words, the non-tumor ovary was not modelled in this work. Please revise the text to clearly reflect this for clarity; specifically, please explain that "nECMPhys" is not a model of the normal ovary, or, if it is, please address in what way it is "normal", given that tumor cells and CAFs seem to have been used for both models.

We thank the reviewer for this comment and apologize for the lack of clarity in the original text. We agree that nECMPhys should not be interpreted as a model of the normal ovary. Importantly, both nECMPhys and tECMHyp (now renamed PLR and HHR, respectively) incorporate HGSC tumor cells. Non-tumor ovarian tissue was not modeled in this study. Rather, these models were designed to recapitulate distinct microenvironmental states relevant to HGSC progression, specifically differences in oxygen tension and ECM remodeling. The term nECMPhys was originally used to denote a physiologically organized ECM state under physoxic oxygen conditions, representative of the microenvironment in which early-stage HGSC lesions arise within the ovary or fallopian tube. It was not intended to imply the absence of tumor cells. To avoid further ambiguity, we have revised the terminology throughout the manuscript. The models are now referred to as PLR (physoxia–low ECM remodeling) and HHR (hypoxia–high ECM remodeling). These terms more accurately reflect the defining biological features of each condition and avoid the unintended implication that PLR represents a normal, non-tumor ovary.

Similarly, the decellularized human ovaries are now referred to as dPLR that stands for decellularized physoxia-low remodeling of ECM model and dHHR for decellularized hypoxia-high remodeling of ECM model.

The following changes have been made:

Introduction: Page 4, Lines 89-90: "Importantly, tumors initiate in tissue characterized by physoxic oxygen levels, low ECM remodeling and higher levels of immune infiltration[1, 26]"

Introduction: Page 4, Lines 95-101: "Such comparisons fail to account for the physoxic oxygen levels present in the tissue microenvironment in which HGSC initiation and early progression occur, as well as the progressive hypoxic conditions characteristic of advanced-stage disease. As a result, critical oxygen-dependent tumor–stroma–immune interactions are often inadequately modeled. To more accurately study these processes, hypoxic conditions representative of late-stage ovarian cancer should be evaluated in comparison to physoxic oxygen levels that reflect the early-stage HGSC microenvironment, rather than to atmospheric oxygen."

Introduction: Page 5, Lines 102-111: "In this study, we bioengineered 3D HGSC models that recapitulate disease-relevant oxygen tensions and ECM remodeling states associated with early and advanced stages of tumor progression, enabling the investigation of cell-cell, cell-ECM interactions, and immune infiltration patterns in a controlled yet physiologically informed context."

Introduction: Page 6 Lines 128-134 " Our work provides a paradigm-shifting understanding of intratumoral hypoxia in advanced HGSC by contrasting hypoxic tumor microenvironments with physoxic oxygen levels characteristic of early-stage disease. This approach provides mechanistic insight into

hypoxia-induced CAF-driven ECM remodeling and its impact on immune evasion, cellular interactions, and facilities screening of targeting vulnerabilities to reprogram immunologically cold HGSC tumors into hot ones."

Results: Page 6, Lines 138-147: "To investigate the role of physiologically relevant oxygen levels and ECM remodeling states in regulating immune infiltration and immune-cancer-stroma interactions across high-grade serous carcinoma (HGSC) progression, we bioengineered a pair of patient-derived 3D models, termed PLR (physoxia–low ECM remodeling) and HHR (hypoxia–high ECM remodeling). These models are based on the crosslinking of fibrinogen into fibrin naturally present in peripheral blood plasma, generating biologically relevant extracellular matrices (ECM) that recapitulate key features of HGSC tissue contexts[30, 31]. HHR is a 3D scaffold designed to model the aberrant, highly remodeled ECM characteristic of advanced-stage HGSC tumor microenvironments when cultured under hypoxic conditions. In contrast, PLR reflects features of early-stage HGSC microenvironments with low ECM remodeling and physoxic oxygen levels when incubated in a standard incubator (Fig. 1a). "

Figures: the acronyms in all Figures and Figure legends have been changed

Results: Page 8, Lines 192-194: "Therefore, this functional analysis revealed that PLR and HHR biomimetic models can recapitulate the physiologically relevant oxygen levels in the site of tumor origin and the intratumoral hypoxia in advanced stage HGSC-TME, respectively."

Results: Page 9, Lines 216-221: "By modulating oxygen availability and ECM composition, we were able to generate culture conditions that align transcriptomically to HGSC tumors and functionally recapitulate oxygen and ECM remodeling associated with early and advanced stages of ovarian cancer initiation and progression. This system therefore enables us to systematically study how oxygen levels and ECM remodeling influence tumor cell behavior and tumor-immune interactions within a physio- and pathologically relevant 3D context."

Results: Page 24, Lines 578-581: "Therefore, we refer to them as, decellularized physoxia-low remodeling of ECM model (dPLR) and decellularized hypoxia-high remodeling of ECM model (dHHR)."

Discussion: Page 24, Lines 597-606: "In this study, we bioengineered patient-derived tumor-immune models that capture biologically representative microenvironments, enabling translational investigation of tumor-stroma-immune dynamics during tumor initiation and progression. By integrating physiologically relevant oxygen levels and extracellular matrix (ECM) cues, these models overcome fundamental limitations of existing preclinical immune models, which fail to reproduce key tumor characteristics such as physoxia, hypoxia, and stromal-driven matrix remodeling. Specifically, our approach reconstructs the oxygen and ECM landscapes of physoxic early-stage and hypoxia-induced ECM remodeling in advanced high-grade serous carcinoma (HGSC)."

Discussion: Page 25, Lines 616-619: "This study demonstrates that hypoxia-induced CAF-driven ECM remodeling impairs immune infiltration, which is in line with the literature[37-40], while using physoxic ovarian environments as a better control for modeling the gradient between the tumor initiation site towards advanced hypoxic tumors."

Discussion: Page 26, Lines 624-625: "Advanced-stage tumors are also characterized by an aberrant stiffer ECM, with a distinct composition from normal tissue where tumor initiates[22, 45]"

Discussion: Page 26, Lines 637-642: "To the best of our knowledge, these human-relevant tumor-immune models uniquely integrate oxygen conditions of the hypoxic advanced-stage tumor microenvironment alongside the physoxic milieu of tumor initiation, while integrating CAFs and patient-derived materials, including tumor biopsies, peripheral blood mononuclear cells (PBMCs), and plasma, enabling interrogation of HGSC tumor microenvironment interactions that are largely inaccessible in existing systems."

Discussion: Page 32, Lines 791-794 "In summary, we have shown that recapitulation of the hypoxic tumor levels in advanced stage in comparison to the physoxic oxygen levels in tumor initiation as a more physiologically relevant control are essential to gain insights into tumor-stroma-immune interactions and model immunologically cold HGSC tumors."

Methods: Page 35, Lines 860-865: The "Specifically, scaffolds were incubated either in 1.5% O_2 hypoxic incubator to recreate the oxygen levels in the hypoxic advanced stage HGSC-TME with high ECM remodeling originating the HHR model (hypoxia-high remodeling of ECM) or in 21% O_2 incubator recreating the physoxic oxygen levels in the site of tumor initiation or early stage of the disease characterized by physoxic oxygen levels and low ECM remodeling generating the PLR model (physoxia-low remodeling). When needed, the cells were isolated for further analysis by digesting the 3D scaffolds with 2000 U/ml collagenase I for two hours at 37ºC."

6. - Fig 2: It is unclear why figure 2, which contains crucial details on the model development, is introduced after figure 1. Would it not make more sense to swap the order of these figures so as to linearly present the model characteristics and then show these lead to the phenotypes assessed in the scRNA-Seq? Please elaborate.

The authors appreciate the reviewer's comment and would like to thank them for the valuable suggestion. The authors agree and have made the following changes:

Figures: Figure 2 a-g were changed to Figure 1 as Figure 1 panels "h-k" and Previous Figure 1 b-j was transferred to Figure 2 a-i. Similarly Supplementary Figure 4 was transferred as Supplementary Figure 1 and 2.

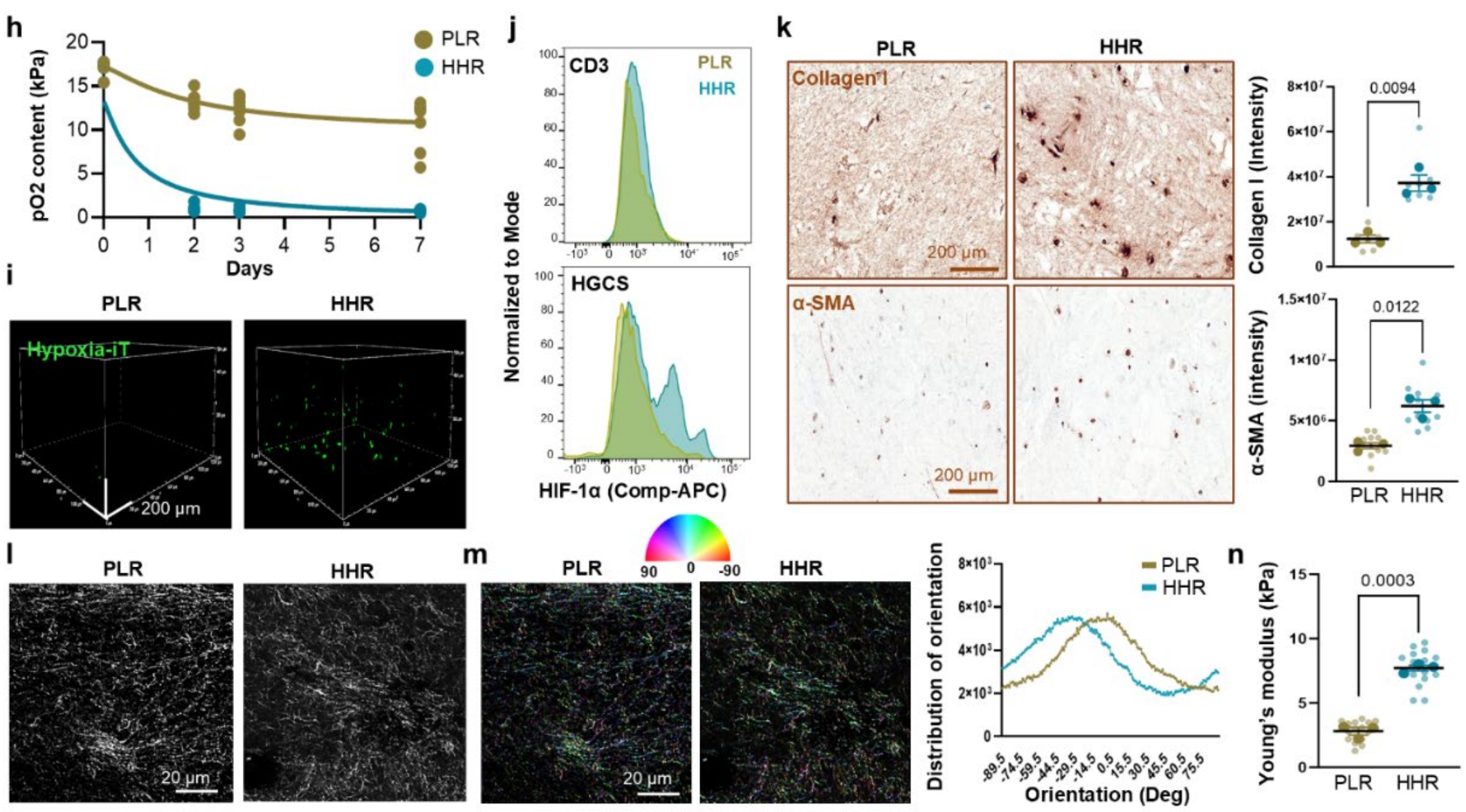

Fig. 1. Transcriptomic concordance and functional divergence across hypoxia and ECM remodeling in patient-derived 3D models. h Oxygen levels measured inside PLR and HHR models (0, 2, 3, and 7 days) by an optical sensor. n=7. **i** Confocal microscopy images in 3D view of hypoxic cells detected with Image-iT™ Green Hypoxia dye. z=500 μm, scale=200μm. **j** Histograms of HIF-1α expression in co-cultured primary HGSC tissue with autologous PBMCs. n=1 **k** Representative IHC images for collagen I and α-SMA in co-culture of KURAMOCHI with CAFs and their quantification, scale=200μm. n=3 (3 ROIs). **l** SHG images of collagen fibers in co-culture and **m** quantification of distribution of orientation. Scale=20μm. **n** Stiffness quantification by AFM represented by Young's Modulus (kPa) in co-cultures n=3 (5 measurements). k, n Mean±SD, t-test.

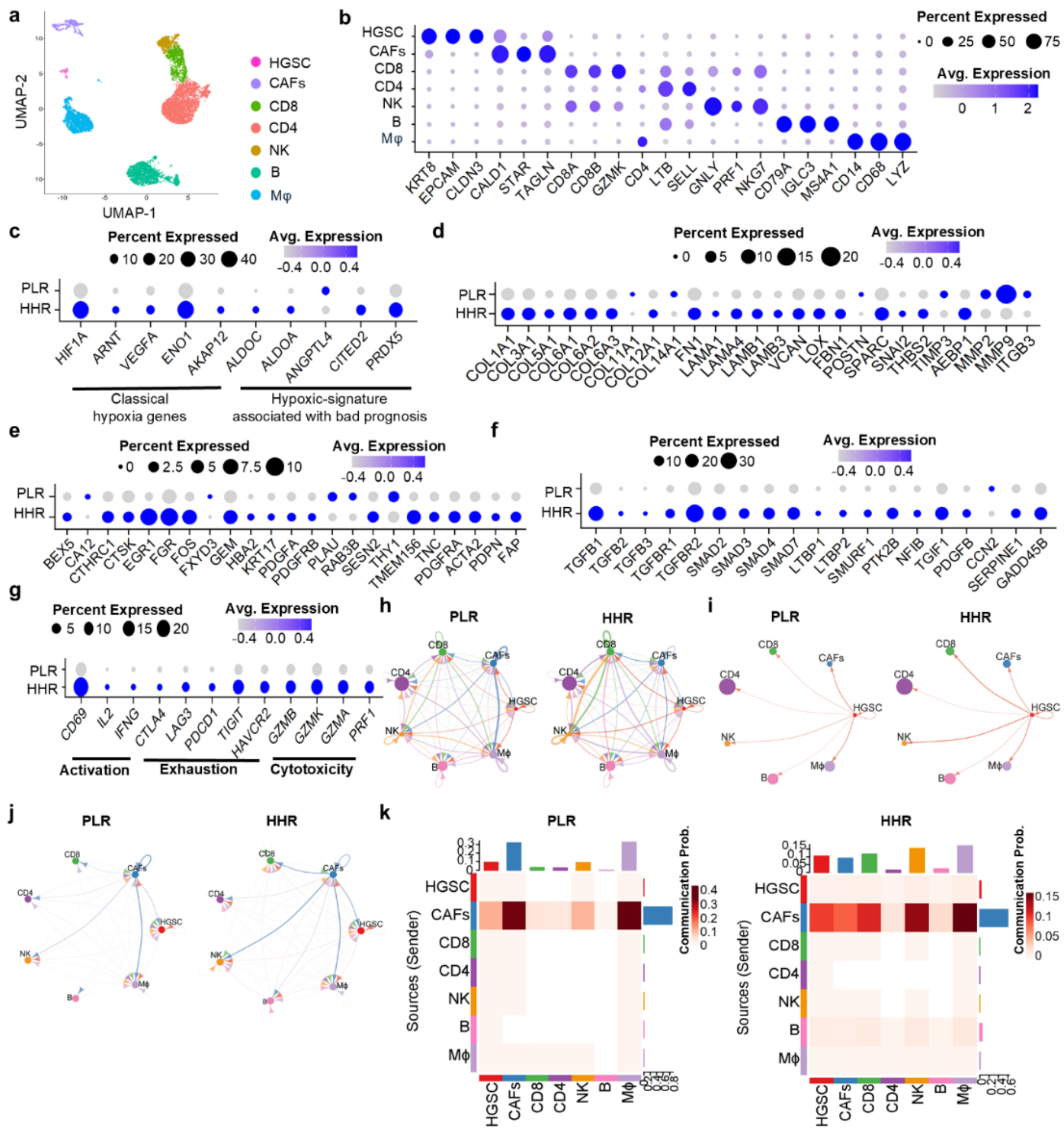

Fig.2. Single-cell RNA sequencing identifies intratumoral hypoxia as a driver of ECM remodeling, immune functionality, and tumor-immune interactions. a UMAP plot showing clusters of annotated cell populations. **b** Bubble plot representing the expression of specific marker genes in each detected cell cluster. **c** Bubble plots showing the gene expression of classical hypoxia genes and hypoxic signature correlated with bad HGSC prognosis, **d** matrisome, **e** CAF-associated genes , **f** TGF-β signaling genes, and **g** T cell functionality genes (activation, exhaustion, cytotoxicity) in co-culture of HGSC tumors with PBMCs inside the PLR vs HHR. **h** Circle plots of interactions (weights/strength) among all cell populations, and **i** HGSC cells with the rest. **j** Circle plots of interactions (weights/strength) among cells in the collagen pathway. **k** Heatmap representing the probability of communication among cells in the collagen pathway.

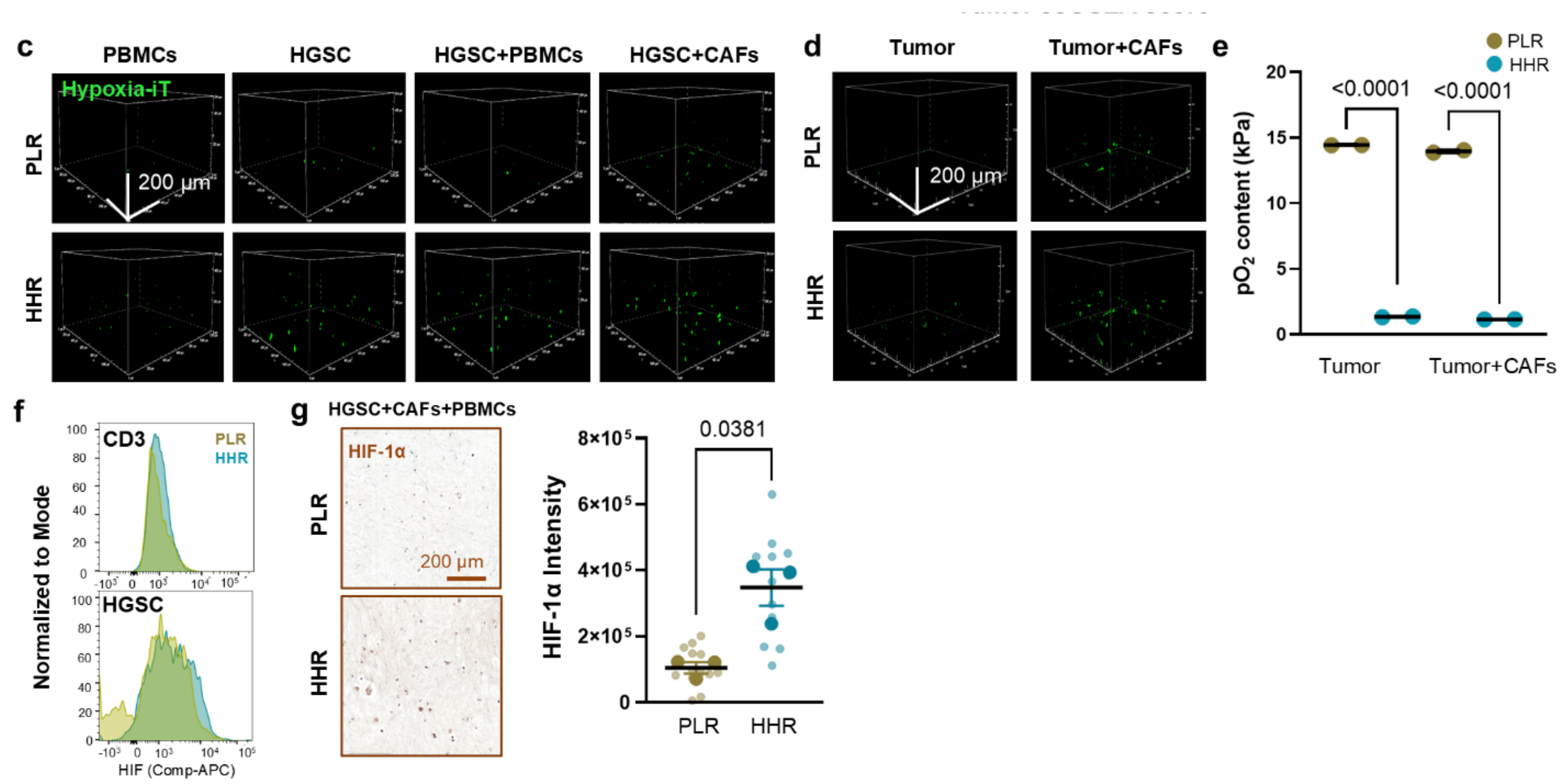

Supplementary Fig. 1. PLR and HHR models conserve the molecular fingerprints of HGSC tumor and recapitulate the physiologically relevant oxygen levels in early vs advanced HGSC. c Representative confocal microscopy images in 3D view of hypoxic cells (green) in monoculture of PBMCs, and HGSC (KURAMOCHI) or in co-cultures of HGSC+PBMCs, HGSC+CAFs or **d** dissociated HGSC tumor in the presence or absence of CAFs in PLR and HHR. Scale=200µm**. e** Oxygen levels measured at day 7 by an optical sensor in HGSC tumor in monoculture or co-culture with CAFs inside PLR and HHR. Mean±SD, n=2, ANOVA. **f** HIF-1α detected by flow cytometry in primary HGSC tissue or PBMCs in monocultures and **g** by IHC in multi-culture of KURAMOCHI, CAFs and PBMCs. Scale= 200µm**.** Mean±SD, n=3 (4 ROIs), t-test.

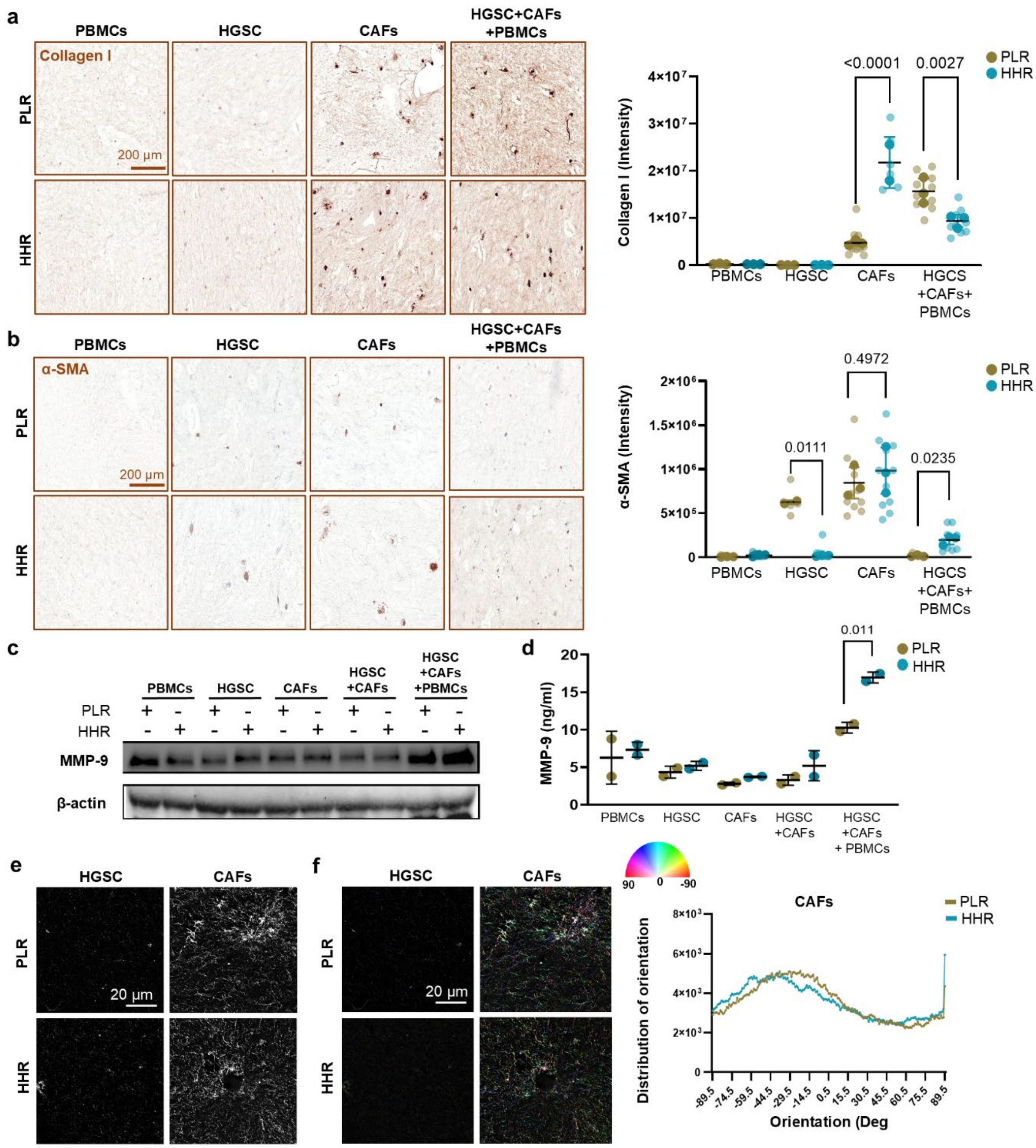

Supplementary Fig. 2. HHR mimics hypoxia-induced CAF-driven ECM remodeling. a-b Representative IHC images and quantification of collagen I and α-SMA intensity in monoculture of PBMCs, HGSC (KURAMOCHI), CAFs or their multi-culture. Mean±SD n=3 (4 ROIs). **c** MMP-9 detected by western blot or **d** ELISA, Mean±SD, n=2. **e** SHG microscopy images in monocultures and **f** the quantification of the distribution of orientation of collagen fibers. a,b,d ANOVA.

Results Page 8-9, Lines 174-221**:** The order of the corresponding results sections was also changed.

7. - Fig 2b: Do patient-derived tumor cells also show hypoxia by this fluorescent marker?

The authors would like to thank the reviewer for this comment and the suggestion of this experiment. Dissociated tumors in presence or absence of CAFs were grown for 7 days in our 3D models in physoxia

compared to hypoxia and stained with the hypoxia-sensitive dye. Green hypoxic cells were only observed in the hypoxic 3D model, similar to the results in our cell lines. Moreover, O_2 levels inside of the 3D scaffolds recapitulating physoxia vs hypoxia were confirmed using an optical sensor.

The following Figure has been included in the manuscript:

Figures: Supplementary Fig 1d,e + Legends

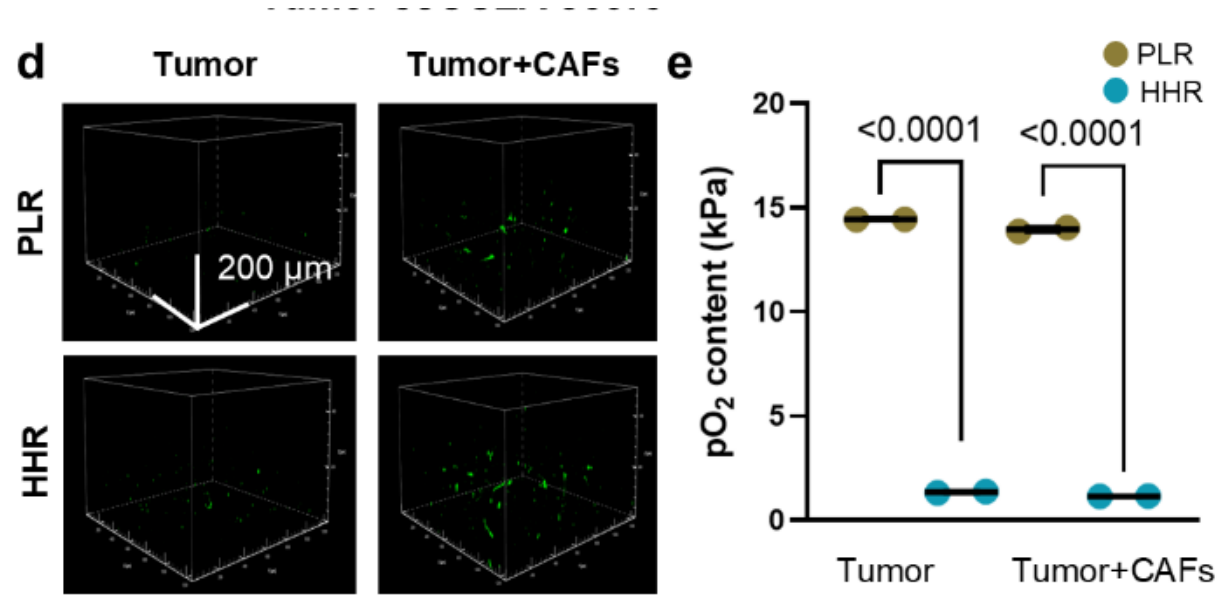

Supplementary Fig. 1. PLR and HHR models conserve the molecular fingerprints of HGSC tumor and recapitulate the physiologically relevant oxygen levels in early vs advanced HGSC d dissociated HGSC tumor in the presence or absence of CAFs in PLR and HHR. Scale=200µm**. e** Oxygen levels measured at day 7 by an optical sensor in HGSC tumor in monoculture or co-culture with CAFs inside PLR and HHR. Mean±SD, n=2, ANOVA.

Results: Page 8, Lines 184-187: "Similar results were observed when dissociated HGSC tumor was grown in monoculture or co-culture with CAFs using hypoxia-sensitive green dye (Supplementary Fig. 1d) and physiologically relevant O_2 levels were confirmed by the optical sensor reaching approximately 14 kPa in PLR and 1.2 kPa in HHR (Supplementary Fig. 1e)."

Methods: Page 39, Lines 948-949: "Alternatively, dissociated HGSC tumors were grown in both 3D models."

Methods: Pages 41-42, Lines 1017-1022: "Hypoxic status of the cells grown in PLR or HHR was detected by using Image-iT^{TM} Green Hypoxia Reagent (5 µM,) which fluoresces green in environments with oxygen levels lower than 5%. KURAMOCHI, CAFs, or PBMCs in monoculture or their combinations in multi-culture KURAMOCHI:CAFs, or KURAMOCHI:CAFs:PBMCs or alternatively, dissociated HGSC tumors were cultured in PLR or HHR for 7 days and the hypoxia-sensitive dye was added 24 h prior to imaging."

8. - Fig 2: for co/culture experiments with CAFs, were these primary CAFs sourced from digest? If not, please elaborate on the source and characteristics of these CAFs.

The authors would like to thank the reviewer for their comments. We apologize that we reported the source of CAFs in the Methods but did not elaborate on their characteristics. The CAFs used in this article were previously developed in our laboratory by exposing primary normal human fibroblasts to conditioned media from KURAMOCHI cells. Phenotypic and functional characterization demonstrated that the conditioned CAFs expressed a CAF-like phenotype, strengthened proliferation, secretory, contractility, and ECM remodeling properties when compared to resting normal fibroblasts, consistent with an activated fibroblast status. Moreover, the reprogrammed CAFs significantly enhanced drug resistance and tumor progression. This was the first publication that provided a comprehensive functional characterization of reprogrammed CAFs using tumor-derived conditioned media and that mechanistically

linked the reprogramming to non-vesicular paracrine signaling. However, we acknowledge that future studies should evaluate the use of primary CAFs.

The authors have incorporated the reviewer's suggestions and included the following changes:

Methods: Page 33, Lines 808-813: **"**Ovarian cancer-associated fibroblasts (CAFs) were developed in our laboratory by reprogramming primary human uterine fibroblasts (ATCC PCS-460-010) with conditioned media from KURAMOCHI cells[56]. The reprogrammed CAFs were phenotypically and functionally validated demonstrating an activated CAF-like state characterized by enhanced proliferative, secretory, contractile, and ECM-remodeling properties relative to normal fibroblasts, as well as the ability to promote tumor progression and drug resistance via non-vesicular paracrine signaling."

Discussion: Page 31, Lines 756-761: **"**We used fully characterized reprogrammed HGSC-CAFs generated in our lab[56] that resemble primary CAFs while exhibiting high CAF and ECM/matrisome-scoring which were linked to the mesenchymal subtype with the worst prognosis in ovarian cancer[32]. However, future studies should confirm the results using primary CAFs, bearing in mind they constitute a heterogeneous population that can lead to pleiotropic responses and present limited cell passage number and growth rate[57]."

9.- Fig 3: infiltration of various immune cells was observed to be decreased in CAF co-cultures in hypoxia, compared to normoxia and authors conclude that this was mediated by a higher amount of ECM remodeling in hypoxic conditions. It would be interesting to make a distinction between this conclusion and the alternative, which is that in hypoxic conditions, co-culture cells do not secrete as many chemo/cytokines to attract immune cells, by performing a cytokine analysis on the supernatants in hypoxic vs normoxic co-cultures prior to adding PBMCs.

We thank the reviewer for this insightful comment and agree that reduced immune infiltration under hypoxic conditions could, in principle, arise either from increased physical barriers due to ECM remodeling or from altered secretion of immune-attractant chemokines by tumor and stromal cells. To directly address this alternative explanation, we performed a Luminex on conditioned media and digested matrices under PLR and HHR conditions in the absence of PBMCs, prior to immune infiltration assays. We focused on key chemokines implicated in immune cell recruitment, including CXCL10, CCL2, and MIF. All three chemokines were significantly increased in tumor–CAF co-cultures compared to either tumor or CAF monocultures, indicating that co-culture enhances immune-attractant potential. Importantly, CXCL10, a critical chemoattractant for T cells and NK cells, was significantly elevated in HHR compared to PLR co-cultures, indicating that hypoxic co-cultures retain, and even augment, their capacity to produce immune-recruiting signals.

These findings indicate that the reduced immune infiltration observed under hypoxic conditions is not due to diminished chemokine secretion, but rather is consistent with hypoxia-induced, CAF-driven ECM remodeling acting as a physical and functional barrier to immune cell infiltration. The new data are presented in Supplementary Fig. 7d and have been incorporated into the Results and discussion sections.

Figure: Supplementary Fig. 7d

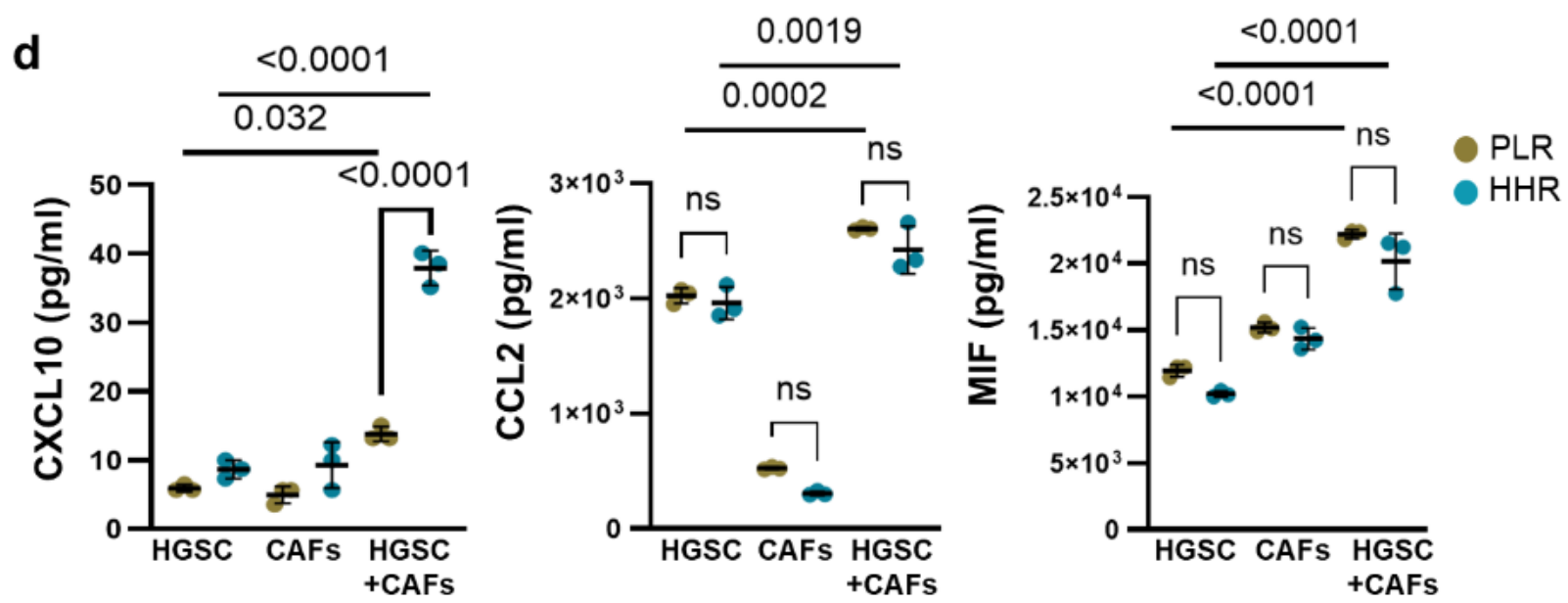

Supplementary Fig. 7. Intratumoral hypoxia induces impaired immune infiltration.

d Quantification of secreted chemokines from KURAMOCHI, CAFs and their co-culture in PLR or HHR on day 7. Mean±SD, n=3. d ANOVA , ns=not significant.

Results: Page 17 Lines 409-420: "To distinguish between impaired immune infiltration due to reduced chemotactic signaling versus increased physical barriers, we quantified immune-attractant chemokines secreted by tumor and stromal cells under PLR and HHR conditions prior to PBMC addition. CXCL10, CCL2, and MIF were significantly increased in tumor-CAF co-cultures compared to either monoculture, indicating that tumor-stromal interactions enhance immune-attractant potential. Notably, CXCL10, a key chemoattractant for T cells and NK cells[34], was significantly elevated in HHR relative to PLR co-cultures, despite reduced immune infiltration under hypoxic conditions (Supplementary Fig. 7d). These results indicate that hypoxic tumor-CAF co-cultures retain, and in the case of CXCL10 enhance, the production of immune-attractant chemokines despite reduced immune infiltration. Together with the increased ECM remodeling observed under HHR conditions, these findings are consistent with a model in which hypoxia-associated, CAF-driven ECM remodeling contributes to impaired immune cell infiltration independently of chemokine availability."

Discussion: Page 28 Lines 688-692: "Even though hypoxia has been reported to suppress immune-attractant chemokine production in some contexts, our data indicate that in HGSC tumor-CAF co-cultures in hypoxia does not diminish, and may enhance, CXCL10 secretion. This suggests that physical and biochemical properties of the remodeled ECM, rather than loss of chemotactic signaling, represent a dominant barrier to immune infiltration in advanced-stage disease."

Methods: Page 47 Line 1160: ProcartPlex Immunoassays were renamed to Luminex Immunoassays

Methods: Page 47 Lines 1164-1166: "Customized Human Luminex®Discovery Assay (R&D Systems) (Table 2) was used to detect chemokines in supernatants of digested 3D scaffolds and culture media mixed in a 1:1 ratio and collected from KURAMOCHI, CAFs and their co-culture."

References: Added new reference #34: Li, W., Ma, J.A., Sheng, X. & Xiao, C. Screening of CXC chemokines in the microenvironment of ovarian cancer and the biological function of CXCL10. *World J Surg Oncol* **19**, 329 (2021).

10. Fig 4: Here the authors observed that treatment with galunisertib decreased collagen expression in hypoxic co-cultures but increased it in normoxic co-cultures. This is surprising given that galunisertib is widely used to inhibit TGFb-mediated phenotypes across a number of models and biological settings. Please elaborate further the interpretation of these data.

We thank the reviewer for highlighting this unexpected observation. We have expanded the Discussion to explicitly address the divergent effects of galunisertib under physoxic versus hypoxic conditions and propose a hypothesis based on oxygen-dependent differences in pro-fibrotic signaling regulation and pathway integration. These revisions are now included in the Discussion section.

Discussion: Page 30, Lines 730-736: "In contrast, under physoxic conditions Gal treatment was associated with increased collagen I levels in tumor–CAF co-cultures, indicating a context-dependent response to TGF-β receptor I inhibition. We hypothesize that under hypoxic conditions collagen production is more tightly coupled to TGF-β–dependent signaling through HIF-1α, rendering it more sensitive to galunisertib treatment, whereas under physoxic conditions compensatory pro-fibrotic responses may offset TGF-β receptor inhibition, resulting in a net increase in collagen expression[35, 81]."

References: Added new reference #81: Qin, Z., Xia, W., Fisher, G.J., Voorhees, J.J. & Quan, T. YAP/TAZ regulates TGF-beta/Smad3 signaling by induction of Smad7 via AP-1 in human skin dermal fibroblasts. Cell Commun Signal 16, 18 (2018).

11. Discussion: a discussion is needed on the confounding variable that plasma from cancer patients seems to only have been used only in the hypoxic model, despite conclusions being made on immune cell infiltration and ECM deposition being differentially observed in the hypoxic condition, compared to the normoxic condition. Conversely, if authors did indeed utilize plasma for normoxic and hypoxic conditions, this requires a much clearer explanation of which was used for what experiments and a discussion of what differences were found between these plasma sources which could confound the conclusions drawn.

We thank the reviewer for raising this important point regarding potential differences between plasma sources. As clarified in our response to Major Comment #1, matched cancer-patient plasma was used for all patient-derived HGSC models under both physoxic and hypoxic conditions, eliminating plasma source as a confounding variable for oxygen-dependent comparisons within these models. Healthy-donor plasma was used exclusively in cell line–based assays, where matched patient plasma is not available.

With respect to potential intrinsic differences between healthy and cancer-derived plasma, we note that in our previous work using human plasma–derived 3D cultures in breast cancer models, we systematically compared healthy donor and cancer patient plasma and found no significant differences in fibrinogen content, the key structural component governing fibrin crosslinking, nor in global cytokine profiles assessed by human cytokine arrays (Cancers, 2020). While this prior analysis was performed in a different cancer context, it supports the notion that plasma-based matrix formation and baseline cytokine composition are not inherently altered in a manner that would confound ECM assembly or immune infiltration assays.

Importantly, all conclusions in the present study are drawn from within-model comparisons (i.e., physoxic vs hypoxic conditions using the same plasma source), and not from comparisons across models generated with different plasma origins. Therefore, differences in plasma source do not confound the conclusions regarding oxygen-dependent ECM remodeling or immune cell infiltration.

Please note all changes reported in major comment #1 and new discussion content about differences across plasma sources included below:

Discussion: Page 31 Line 768-773: "While the present study does not directly compare plasma composition between healthy donors and HGSC patients, our prior work using human plasma–derived 3D

cultures demonstrated comparable fibrinogen content and baseline cytokine profiles between healthy and cancer patient plasma [31] supporting the robustness of plasma-based matrices and minimizing the likelihood that plasma source alone confounds ECM formation or immune infiltration outcomes."

Minor comments:

1. - In the abstract, the sentence "the models integrate cancer cells co-cultures with CAFs…" is too complicated and confusing. Please revise for clarity.

The authors would like to thank the reviewer for their suggestion and apologize for the complexity of the sentence. The authors have made the following changes in the abstract, introduction and discussion of the manuscript:

Abstract: Page 3, Lines 53-54: "Cancer cells were co-cultured with cancer-associated fibroblasts within human plasma-3D matrices or grown on decellularized human ovaries. Immune cells were either included within the 3D constructs to study multi-cellular interactions or challenged to infiltrate the matrices."

Introduction: Page 5, Lines 105-111: "Peripheral blood mononuclear cells (PBMCs) were incorporated into the system using two distinct and independent experimental configurations. In the first configuration, PBMCs were included within the 3D construct as a triculture, together with HGSC tumor cells and CAFs, allowing direct assessment of tumor–immune interactions within the engineered microenvironment. In the second configuration, PBMCs were challenged to infiltrate either the engineered 3D HGSC models or decellularized human ovarian ECM, enabling analysis of immune infiltration dynamics and immune–ECM interactions"

Discussion: Page 5, Lines 105-111: "The platforms incorporate cancer cells co-cultured with cancer-associated fibroblasts (CAFs) within three-dimensional matrices engineered using autologous patient plasma or decellularized human ovaries, while immune cells are either embedded as multicultures or challenged to infiltrate the matrices."

2. - Fig 1a is further confusing, as it seems to imply that ECM was added to the tumor model, but not to the normal model. Perhaps the authors mean that ECM is eventually deposited at higher levels in the tumor model? Please revise for clarity.

The authors appreciate the reviewer's comment and would like to thank them for the suggestions. The authors meant to express that there was higher and aberrant ECM remodeling in the hypoxic model compared to physoxic model. The authors renamed the 3D models to PLR (physoxia-low remodeling of ECM) and HHR (hypoxia-high remodeling of ECM) to better reflect these changes (*see response to Major comment #5) and Figures 1a and 4a and Supplementary Fig. 10a have been revised accordingly.

Figure 1a

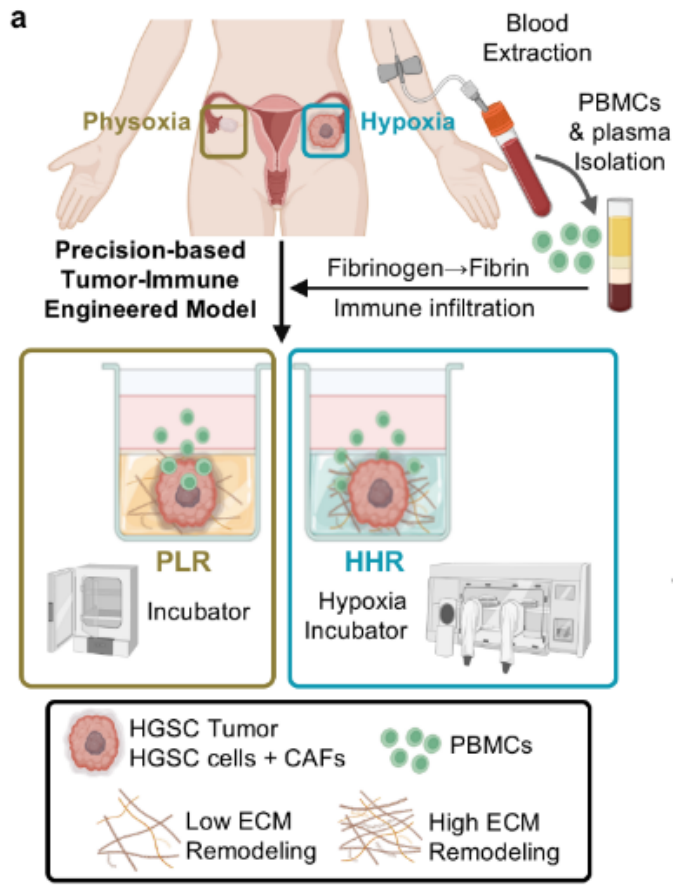

Fig. 1. Transcriptomic concordance and functional divergence across hypoxia and ECM remodeling in patient-derived 3D models. a Schematic of patient-derived 3D models PLR (physoxia-low remodeling of ECM) and HHR (hypoxia-high remodeling of ECM) mimicking O_2 levels and ECM biomechanical cues in the early and late stage HGSC, respectively

Figure 4a

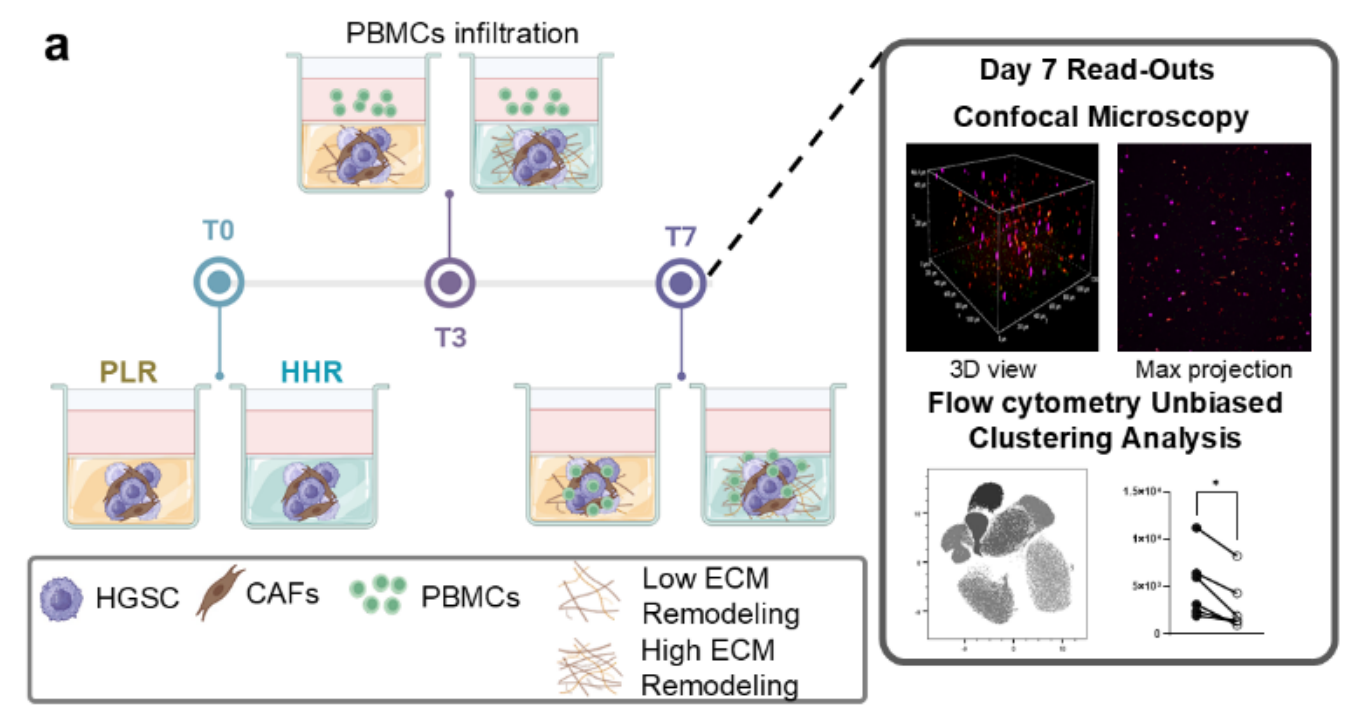

.

Fig. 4. Hypoxia-induced CAF-driven ECM remodeling preserved in HHR model is associated with impaired immune infiltration. a Schematic representation of methods used in immune infiltration studies.

Supplementary Fig. 10a

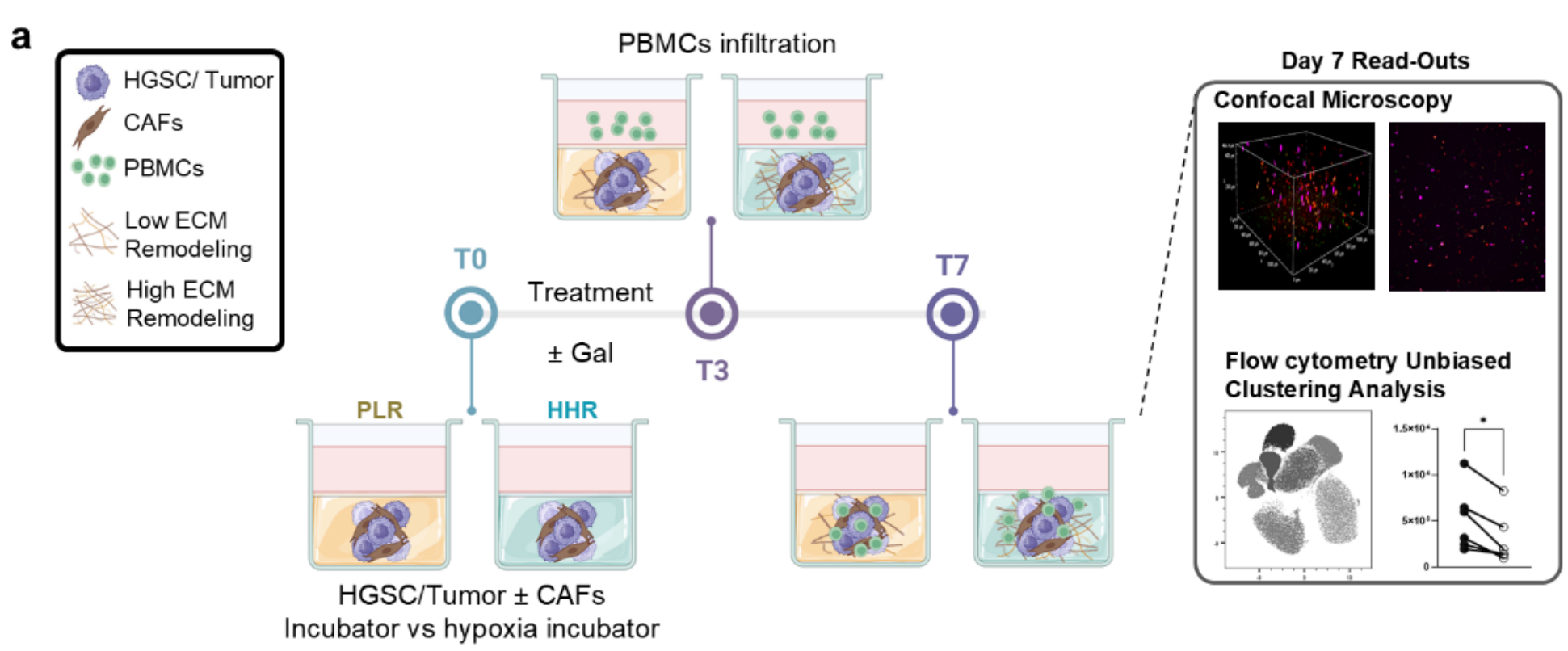

Supplementary Fig. 10. Assessment of galunisertib preincubation in the HGSC patient-derived model. a Schematic representation of infiltration studies in the presence or absence of galunisertib (Gal).

Reviewer #2, Ovarian cancer conventional and immunotherapy (Remarks to the Author):

This manuscript bioengineers patient-derived 3D tumor–stroma–immune models under differential oxygen tensions to interrogate CAF-driven ECM remodeling and immune infiltration, and tests TGF-βR1 inhibition (galunisertib) as a putative "rescue". The topic is important and the platform is promising. However, multiple conceptual, methodological, and statistical issues substantially limit interpretability and translational relevance.

The authors would like to thank the reviewer for their valuable feedback that helped us to improve the manuscript clarity, statistical analysis and data interpretation and to improve the translational relevance.

Major comments:

1. The authors present scRNA-seq/flow data for cellular composition but do not visually validate the 3D spatial architecture. Without multiplex immunofluorescence/immunohistochemistry (mIF/mIHC) or spatial imaging, it is impossible to ascertain whether the model reconstructs spatial patterns relevant to immune exclusion.

We thank the reviewer for raising this important point regarding validation of 3D spatial architecture. To address this concern, we performed additional multiplex immunofluorescence and confocal z-stack imaging to benchmark the spatial organization of HGSC tumors with low and high infiltrated areas against the engineered 3D models. These analyses demonstrate that PLR and HHR models recapitulate spatial immune-infiltrated and immune-excluded architectures, respectively. The new data are presented in Supplementary Fig. 8a–b and have been incorporated into the Results, methods and discussion sections.

The following changes have been incorporated into the manuscript:

Figures: Supplementary Fig. 8 a,b

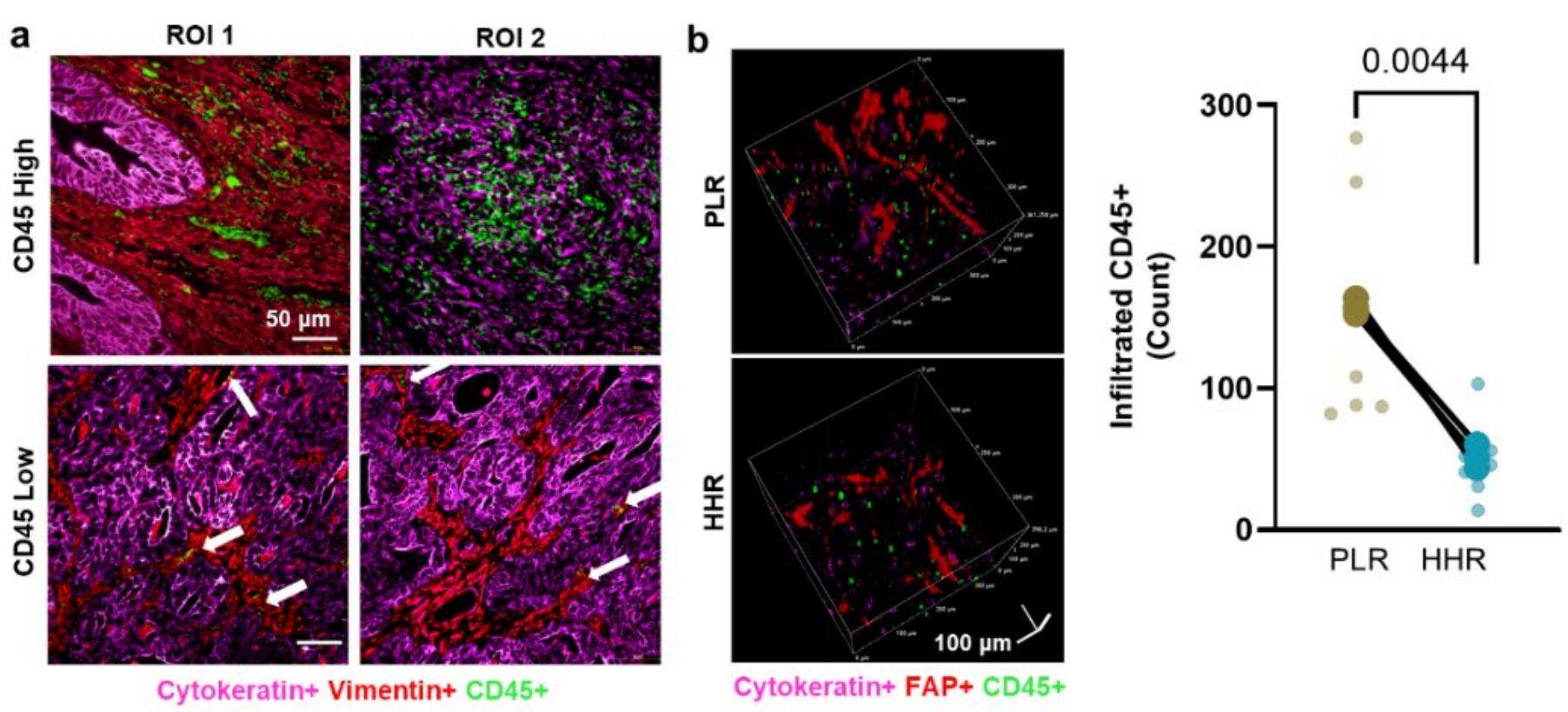

Supplementary Fig. 8. Spatial organization of immune infiltration in HGSC tumors is recapitulated in PLR and HHR 3D models. a Representative immunofluorescence images of two regions of interest (ROIs) from HGSC tumors with areas of low and high CD45 infiltration. Sections were stained for cytokeratin^{+} tumor cells (magenta), vimentin^{+} stromal cells (red), and CD45^{+} immune cells (green). White arrows indicate sparse CD45^{+} immune cell infiltration. Scale=50 µm. **b** Confocal 3D view reconstructions showing infiltration of CD45^{+} immune cells (green) within PLR and HHR 3D models generated from dissociated HGSC tumors, containing cytokeratin^{+} tumor cells (magenta) and FAP^{+} fibroblasts (red) and corresponding quantification of CD45+ cells (counts)**.** Scale=100µm**.** Donor-paired superplot n=3 (3 ROIs), t-test.

Results: Pages 17-18, Lines 421-431: "Once reduced immune infiltration was established by flow cytometry, we next assessed whether these effects were reflected in the spatial organization of immune cells using imaging-based analyses in both HHR/PLR models and HGSC patient tumors. Multiplex immunofluorescence analysis of HGSC tumors with high or low immune infiltration revealed distinct spatial organization of $CD45^+$ immune cells (green) relative to $cytokeratin^+$ tumor cells (magenta) and $vimentin^+$ stromal compartments (red), with increased immune infiltration observed in immunologically hot tumor areas (Supplementary Fig. 8a). To benchmark the engineered 3D models, dissociated HGSC tumor tissues were cultured under PLR or HHR conditions and challenged with autologous PBMCs. Confocal z-stack imaging for CD45 (green), cytokeratin (magenta), and FAP (red) demonstrated significantly impaired immune infiltration in HHR compared to PLR models, recapitulating spatial features observed in immunologically cold tumor areas (Supplementary Fig. 8b)."

Discussion: Page 28, Lines 692-698: "Consistent with the spatial organization observed in HGSC tumors with high or low immune infiltration areas, imaging analysis revealed that the engineered PLR and HHR models recapitulate immune-infiltrated and immune-excluded architectures, respectively, supporting their relevance for studying spatial determinants of immune exclusion. Although limited in sample size, the observed association between collagen-rich regions and reduced $CD8^+$ T-cell infiltration in HGSC patient tissues supports the clinical relevance of the CAF-driven ECM remodeling mechanisms identified *in vitro*."

Methods: Page 33, Lines 818-819: "Paraffin-embedded HGSC tissues were provided by Sanford Biobank, Sioux Falls, SD."

Methods: Page 40, Lines 984-987: "The paraffin-embedded HGSC tissues were processed in similar way. The sections were stained with the Discovery Ultra automated slide staining system with specific antibodies for immunohistochemistry (IHC) or immunofluorescence (IF) (Table 2)."

Methods: Page 41, Lines 1005-1010: "For spatial patterns of immune infiltration, HGSC tissue sections were stained by multiplex IF staining in sequential cycles with target specific primary antibody, then HRP conjugated anti-species secondary antibody and then fluorophores. Epithelial, stromal and immune cells were detected with anti-cytokeratin (magenta), anti-vimentin (red) and anti-CD45+ (green) antibodies (Table 2), respectively. The primary-secondary antibodies complex was removed by heat denaturation using BenchMark CC2 buffer. Finally, the slides were counterstained with Hoechst for nuclei detection."

2. Panels 2h–i (T-cell functional phenotypes) are thematically disconnected from 2a–g (oxygen/ECM mechanics) and align more naturally with the immune-infiltration narrative in Figure 3, which complicates readability and causal logic.

The authors would like to thank the reviewer for their suggestion and agree on moving the panels h-i (T cell functional phenotypes) from the original Figure 2. The authors have created a new Figure 3 and a new section in the results specifically for T cell functional phenotypes in order to allow a better understanding. The following changes have been made:

Figures: Fig. 3 + Supplementary Fig. 6 have been modified

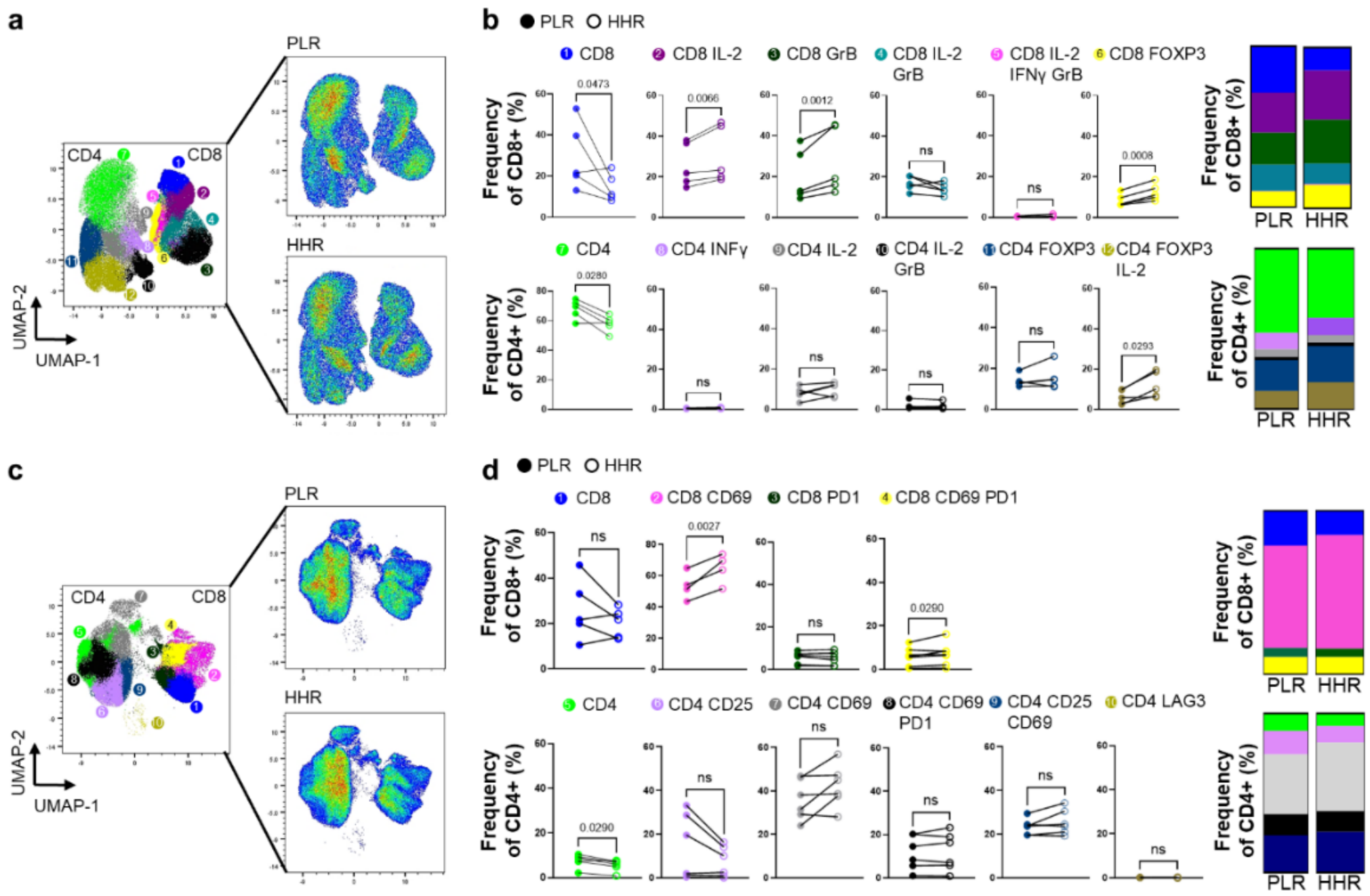

Fig. 3. HHR model recapitulates T cell effector functions of HGSC tumors. a UMAP plot showing clusters of detected populations and density plots comparing PLR vs HHR in co-culture of HGSC tumors with autologous PBMCs inside the 3D models. **b** Donor-paired dot plots representing the expression of intracellular markers of activation (IL-2, IFNγ), exhaustion (FOXP3) and cytotoxicity (granzyme B, GrB) represented as frequency (percentage of parent) in CD4+ and CD8+ T cells. **c** UMAP, density plots, and **d** quantification of activation (CD25, CD69) and exhaustion (PD1, LAG3) surface markers represented as frequency (percentage of parent) of CD4+ or CD8+ T cells. n=5. Paired t-test. ns=not significant.

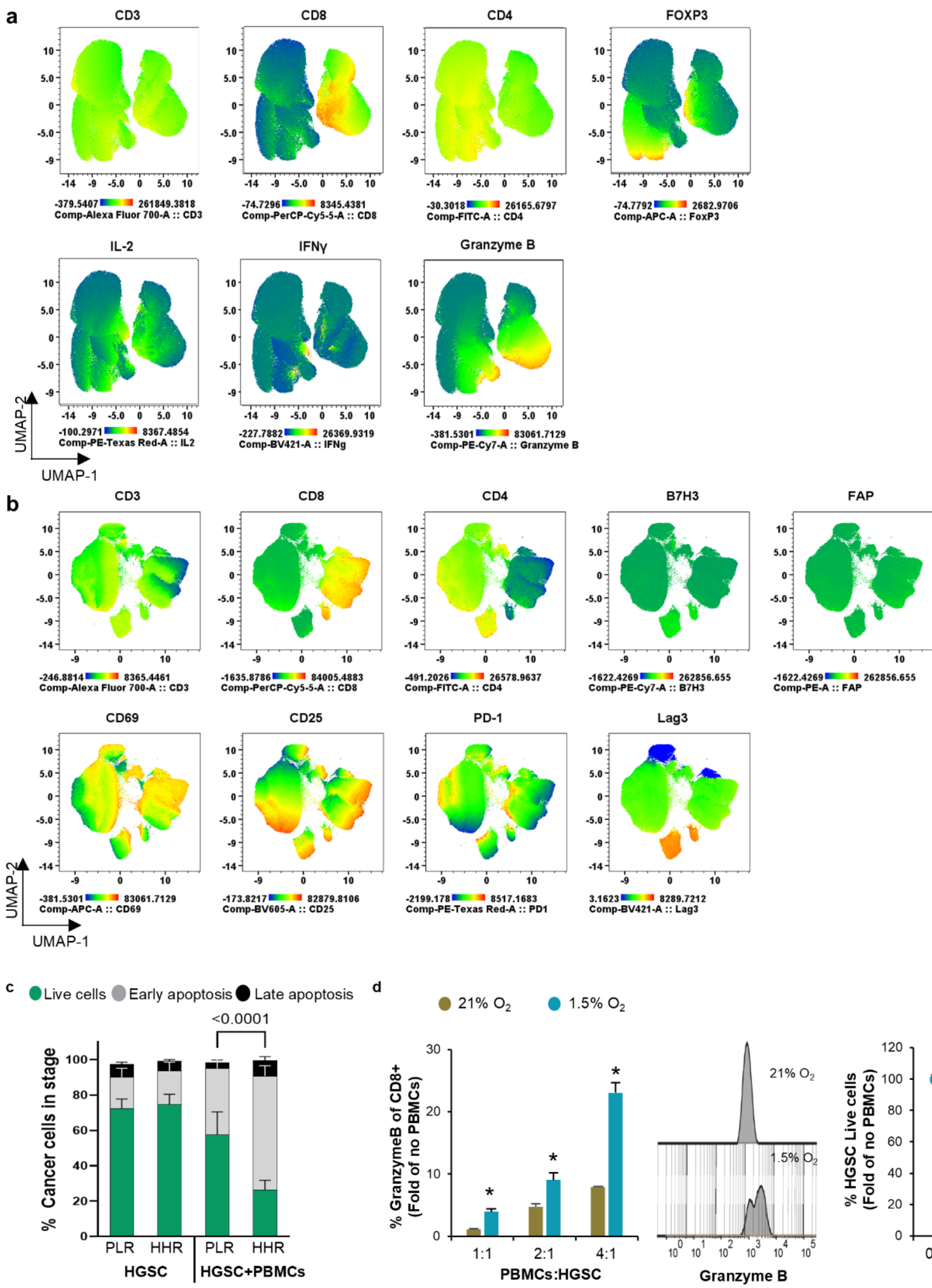

Supplementary Fig. 6. Intratumoral hypoxia induces higher functionality and cytotoxicity of PBMCs. a-b Heatmaps of functionality markers expression. **c** Apoptosis evaluation of KURAMOCHI in the presence or absence of PBMCs in HHR compared to PLRs. **d** Cytotoxicity evaluation and granzyme B expression in PBMCs cocultured with KURAMOCHI in 2D by flow cytometry. n=3. ANOVA, *p<0.05.

Results: Page 13 Line 303: "The HHR model recapitulates T cell effector functions of HGSC tumors"

3. Western blots in figure 4b–c appear overexposed and trends are not convincingly quantified. In Figure 4d (left panel), TGF-β in CAFs under tECMHyp appears lower than under nECMPhys, conflicting with Figure 4c where pathway activity is higher in hypoxia. Moreover, galunisertib targets TGF-βR1 signaling, not ligand production as shown in figure 4a, so why does intracellular TGF-β protein decrease after galunisertib?

We thank the reviewer for these comments and apologize for the inclusion of overexposed western blot images in the original submission. The membranes corresponding to Figures 5b–c and Supplementary Fig. 9a-b were re-stained and re-imaged using appropriate exposure settings, and the updated images have been replaced in the revised manuscript.

With respect to the apparent discrepancy between Figures 5c and 5d, we performed more replicates for this experiment where the discrepancy between the western blot and imaging flow cytometry no longer exists and the intracellular TGF-β expression in CAFs was similarly increased in hypoxia compared to physoxia.We also note that these assays measure different pools of TGF-β. The western blots in Fig. 5c were performed on whole lysates of the 3D constructs, including both cellular and extracellular matrix–associated protein, whereas the imaging flow cytometry analysis in Fig. 5d quantifies only intracellular TGF-β. Therefore, differences in relative signal intensity between these approaches are expected.

Although galunisertib targets TGF-β receptor I rather than ligand production, inhibition of receptor signaling is known to disrupt autocrine and feedback mechanisms that regulate TGF-β expression and intracellular availability. We have revised the Discussion to provide a concise, hypothesis-driven interpretation of why intracellular TGF-β levels decrease following galunisertib treatment.

Figure 5. was modified including new membranes for western blot with lower exposure times and new data for imaging flow cytometry.

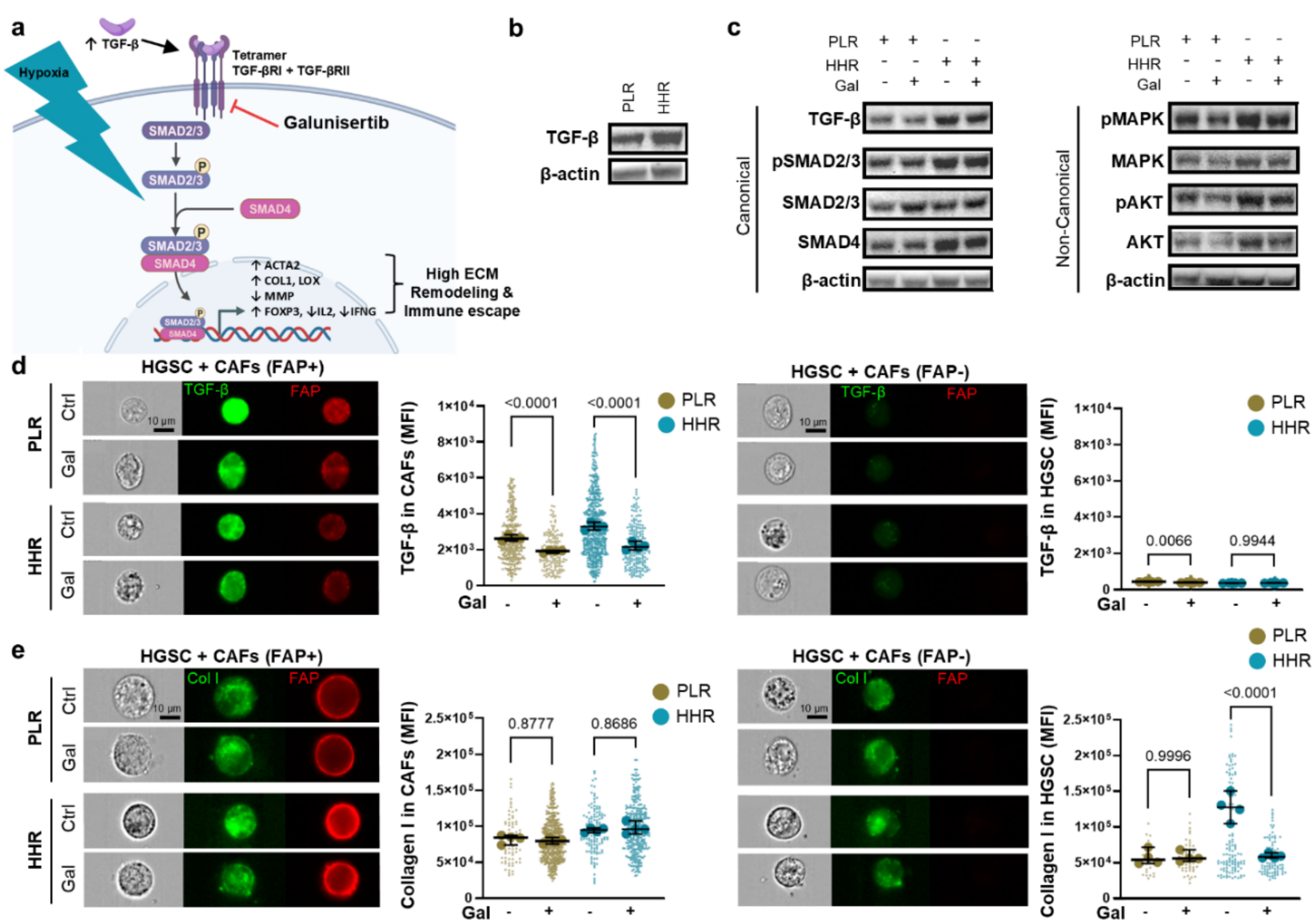

Fig. 5. Preincubation with galunisertib reduces hypoxia-induced CAF-driven TGF-β signaling and collagen I expression recapitulated in HHR model. **a** Schematic of TGF-β signaling pathway involved in ECM remodeling in the hypoxic TME and the mechanism of action of TGF-βRI inhibitor, galunisertib. **b** Representative images of TGF-β expression detected by western blot in co-culture. n=3. **c** Expression of signaling molecules involved in canonical (left) and non-canonical (right) TGF-β signaling pathway in the presence or absence of galunisertib (Gal) detected by western blot in co-culture. n=3. **d** Representative images of intracellular TGF-β (green) and **e** collagen I (green) in imaging flow cytometry in co-culture of CAFs (FAP+, red) and HGSC (FAP-), and quantification of green mean fluorescence intensity (MFI). Scale=10 µm. n=4 (10.000 images). d, e Median±CI95%, ANOVA.

Results: Page 19, Lines 459-462: "Of note, while western blot analysis reflects total TGF-β levels in whole 3D construct lysates including cellular and extracellular matrix-associated protein, imaging flow cytometry represents intracellular TGF-β expression."

Discussion: Page 29, Lines 721-728: "Although galunisertib directly inhibits TGF-β receptor I rather than ligand production, treatment was associated with reduced intracellular TGF-β levels. We hypothesize that this effect reflects disruption of TGF-β–dependent feedback loops and autocrine signaling mechanisms that normally sustain ligand expression and intracellular availability[37, 77, 78]. In addition, differences between whole-lysate western blotting and intracellular imaging measurements likely reflect distinct cellular versus extracellular pools of TGF-β within the 3D constructs. Together, these observations are consistent with context-dependent regulation of TGF-β signaling following receptor inhibition."

References: New References #77 and #78 were added:

77. Serova, M. et al. Effects of TGF-beta signalling inhibition with galunisertib (LY2157299) in hepatocellular carcinoma models and in ex vivo whole tumor tissue samples from patients. Oncotarget 6, 21614-21627 (2015).

78. Akhurst, R.J. Targeting TGF-beta Signaling for Therapeutic Gain. Cold Spring Harb Perspect Biol 9 (2017).

4.In figure5, paired t-tests are applied to comparisons that do not reflect true within-donor pairing. The validity of key significances is uncertain; a correct framework (unpaired or mixed-effects with donor as a random factor and multiplicity control) may materially alter the conclusions.

We thank the reviewer for this valuable suggestion. We have reanalyzed the data using linear mixed-effects models with donor as a random factor and applied multiplicity correction. While some effect sizes were modestly attenuated, **the overall trends and conclusions are preserved**, and the Results and figure legends have been revised accordingly.

The following changes have been made in the manuscript:

Figures:

Figures 6 b,c,e,g,h, Fig. 7, Supplementary Fig. 10c, Supplementary Fig. 11 a,b,d, f,g,h have been modified.

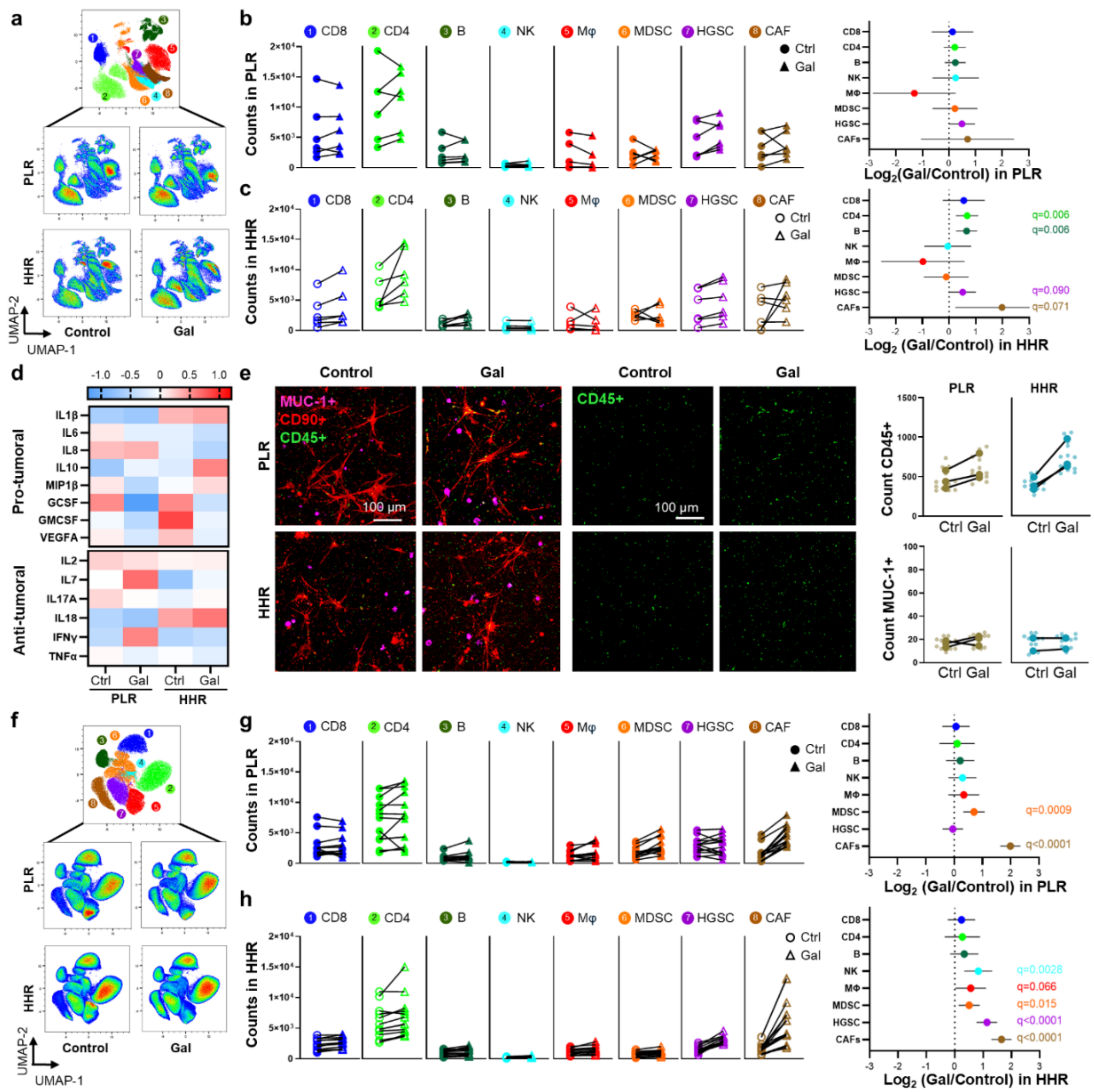

Fig. 6. Preincubation with galunisertib attenuates the hypoxia-induced CAF-driven impaired immune infiltration. **a** UMAP plot, density plots, and **b,c** donor-paired dot plots representing the number (counts) of infiltrated PBMCs cell types (CD3+, CD4+, CD8+ T cells, Mφ (CD14+), NK (CD56+), B (CD19+) and MDSC (CD11b+)), and HGSC cells from dissociated tumors in co-culture with CAFs inside PLR (b) or HHR (c) in the presence or absence of galunisertib (Gal) in dimensionality reduction analysis by flow cytometry. n=6. Forest plots show the $\log_2$ fold changes with 95% confidence intervals of Gal/Control in PLR or HHR. **d** Heatmaps showing the expression of secreted pro-tumoral and anti-tumoral cytokines in HGSC patient samples. Median of z-score for each analyte. n=6. **e** Confocal microscopy images of z-stack view as a maximum projection of infiltration of PBMCs (CD45+, green) into the scaffold with KURAMOCHI (MUC-1+, magenta) and CAFs (CD90+, red) in the presence or absence of galunisertib and quantification of the number of CD45+ and HGSC. Scale=100µm. Donor-paired superplots n=3 (4 ROIs). **f** UMAP, density plots, and **g** donor-paired dot plots showing the number (counts) of infiltrated PBMCs phenotypes and KURAMOCHI and CAFs inside the scaffold in the presence or absence of galunisertib in dimensionality reduction analysis by flow cytometry and forests plots representing $\log_2$ fold changes with 95% confidence intervals of Gal/Control in PLR or **h** HHR. n=13. b, c, e, g, h linear mixed-effect model analysis with Benjamini–Hochberg FDR.

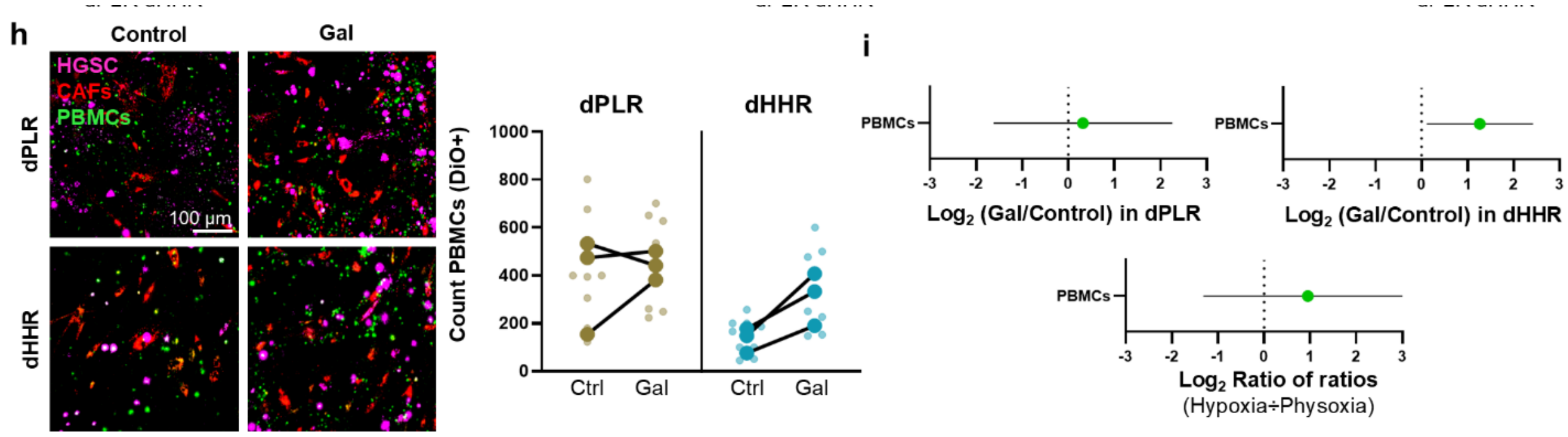

Fig. 7. Galunisertib promotes partial recovery of impaired immune infiltration in ovarian native dECM. g Confocal microscopy images and the quantification of the number of infiltrated PBMCs (green) into the dECM recellularized with KURAMOCHI (magenta) and CAFs (red) in the absence and **h** in the presence of galunisertib. Scale=100µm. Donor-paired superplots n=3 (3 ROIs). **i** Forest plots showing $\log_2$ fold changes with 95% confidence intervals of Gal/Control and $\log_2$ ratio of ratios of interactions model x treatment (hypoxia/physoxia). e, f Mean±SD. e, f, g, t-test; h linear mixed-effect model analysis with Benjamini–Hochberg FDR.

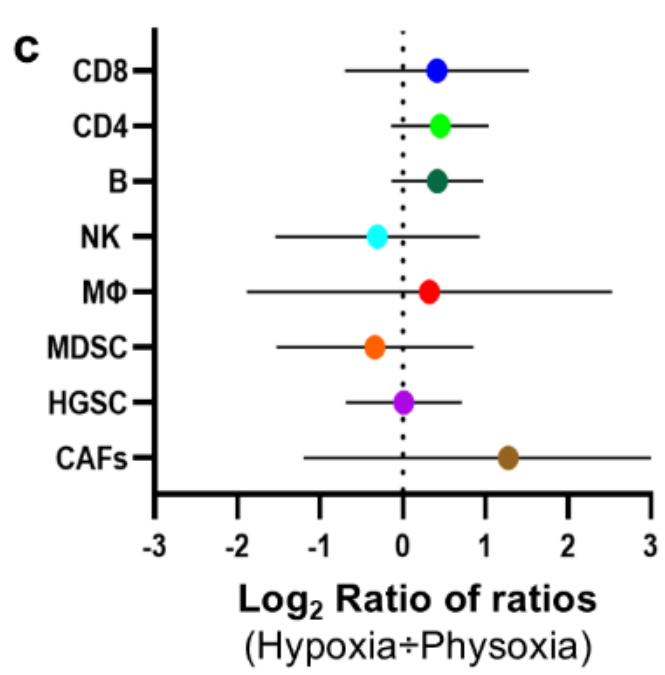

Supplementary Fig. 10. Assessment of galunisertib preincubation in the HGSC patient-derived model.

c Forests graphs representing $\log_2$ ratio of ratios of interactions model x treatment (hypoxia/physoxia) determined by linear mixed-effect model statistical analysis with donor as a random interceptor. Benjamini–Hochberg FDR.

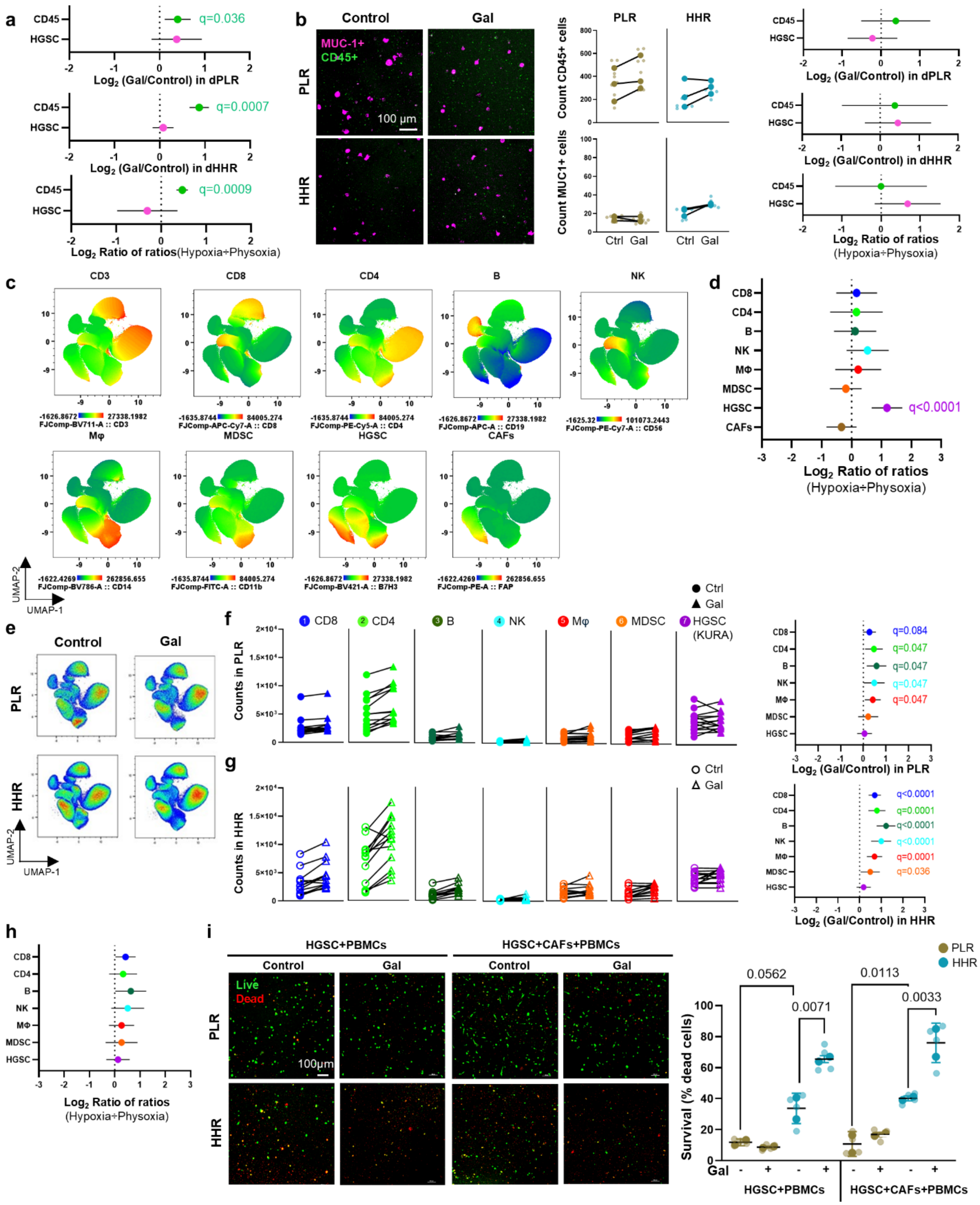

Supplementary Fig.11. Galunisertib attenuates the impaired immune infiltration in the HGSC cell line model. a-b Effects of galunisertib (Gal) on immune infiltration in KURAMOCHI co-culture (a) and monoculture (b) in PLR and HHR assessed by confocal microscopy. Forest plots show $\log_2$ fold changes (Gal/Control) and model × treatment interaction effects (hypoxia/physoxia). Representative confocal images and donor-paired quantifications of infiltrated $CD45^+$ PBMCs. Scale=100 µm; $n = 3$ (3 ROIs). **c-d,** Effects of Gal on immune infiltration in KURAMOCHI co-culture and monoculture in PLR and HHR assessed by flow cytometry heatmaps (c) and forest

plot of interaction in co-cultures (d). **e-h** Effects of Gal on immune infiltration in KURAMOCHI assessed by flow cytometry density plots (e), donor paired plots, forest plots log2 fold changes in PLR(f) and HHR (g), and forest plot of interaction (h). **i** Representative confocal images of Live/dead stain of infiltrated PBMCS in KURAMOCHI or KURAMOCHI co-cultures and survival quantification. Scale=100 µm; Mean±SD, $n = 2$ (2 ROIs). i ANOVA a,b,d,f,g,h linear mixed-effect model with Benjamini–Hochberg FDR.

Tables: Supplementary Table 1 was generated with the results of statistical analysis.

Results: Page 20, Line 487-490: "Treatment with galunisertib significantly increased CD4$^+$ cells and B cells in HLR while the effect was smaller in the rest of immune cells and in PLR (Fig. 6 b,c and Supplementary Table 1). No significant model x treatment interaction was observed (Supplementary Fig. 10c and Supplementary Table 1) demonstrating model-independent drug activity rather than hypoxia/ECM-specific enhancement."

Results: Page 21, Lines 507-510: "Confocal microscopy results showed that preincubation with Gal significantly increased the infiltration of CD45$^+$ immune cells into the 3D matrix under PLR and HHR having significant model x treatment interaction while the cancer signal showed modest, non-significant shifts in both PLR and HHR and no significant interaction (Fig. 6e, Supplementary Fig. 11a, Supplementary Table 1)."

Results: page 21**,** Lines 512-517: "Similar directional effects were observed in CD45$^+$ T cells infiltration in KURAMOCHI monocultures although lacking statistical significance while cancer cells showed a trend toward a hypoxia-enhanced drug effect with higher interaction (Supplementary Fig. 11b)."

Results: Pages 21-22, Lines 518-529: **"**Immunophenotyping results by flow cytometry in KURAMOCHI cells co-cultured with CAFs showed that galunisertib pre-treatment significantly increased the infiltration of MDSCs in PLR and NK cells, macrophages and MDSCs in HHR (Fig. 6f,g,h and Supplementary Fig. 11c, Supplementary Table 1). In KURAMOCHI monocultures the treatment with Gal significantly increased the infiltration CD8$^+$, CD4$^+$, B cells, NK cells, Mφ and MDSC in HHR with more modest but still significant effects in PLR in the same immune cell populations, except MDSC, suggesting hypoxia-permissive environment rather than a cell-type restricted effect (Supplementary Fig. 11e,f,g, and Supplementary Table 1). Nonetheless, no cell population showed a significant model x treatment interaction in either condition (Supplementary Fig. 11d,h). These results suggest that preincubation with galunisertib attenuates the impaired immune infiltration of adaptive immune cells (CD4+ and B cells) in HGSC patients and innate lineage (NK, Mφ and MDSC) in healthy PBMCs in an oxygen/ECM-independent manner."

Results: Page 24, Lines 584-589: "Then, preincubation with galunisertib increased the immune infiltration in hypoxia (q=0.12) with smaller, non-significant effect in physoxia in alignment with our previous results (Fig. 7h,i and Supplementary Table 1). The model×treatment interaction analysis indicated a larger hypoxia/ECM effect but did not reach FDR significance with three patients. Replicate-aware plots confirm parallel control-galunisertib shifts within donors, and ICCs document replicate reliability per condition (Supplementary Table 1)."

Discussion: Pages 30-31, Lines 743-755: "Importantly, parallel to other studies[82, 83], our data indicate that pre-treatment with Gal attenuates the hypoxia-induced CAF-driven impaired immune infiltration of CD45$^+$ immune cells in our plasma 3D models mainly the adaptive immune cells in HGSC-patient derived model and innate lymphoid and myeloid lineage in healthy PBMCs using cell lines within the matrices. Even under physoxic oxygen tension and low ECM remodeling, the drug had detectable immune-modulatory activity, but effect sizes were smaller than under hypoxia and high ECM remodeling across all populations acting through oxygen/ECM-agnostic mechanisms. The differences in the responses between PBMCs from healthy donors and HGSC patients can be correlated with acquired immune resistance in primary cells and an altered cell behavior due to previous chemotherapeutic and

immunotherapeutic clinical exposure in the HGSC patients[84]. Similar trends were observed in decellularized human ovaries, although the limited sample availability constrained the number of replicates and immunophenotyping studies."

Methods: Page 49, Lines 1193-1208: "Paired-t test was used for statistical analysis in immune infiltration assays comparing PPR vs HHR. Linear mixed-effects model was used to assess the statistical significance of the treatment with galunisertib compared to control in i) PLR, ii) HHR and iii) the model x treatment interaction (difference-in-differences, ratio of ratios, ROR). In confocal microscopy dataset (multiple ROIs per donor; n = 3 donors), replicate fields within each model and treatment condition were aggregated on the log scale using a 10% trimmed mean of log(1+abundance) to reduce the influence of outliers and analyzed using a replicate-aware, donor-paired 2×2 factorial framework. Replicate reliability was assessed using intraclass correlation coefficients (ICCs) from random-intercept models.For flow cytometry (one measurement per patient), log-transformed abundances [log(1+abundance)] were analyzed using a 2×2 factorial linear mixed-effects model with donor as a random intercept. Multiple testing was controlled using the Benjamini–Hochberg false discovery rate (FDR) procedure. Effect sizes were reported on the natural-log scale and as back-transformed ratios and $\log_2$ fold changes with 95% confidence intervals and represented on paired donor-level dot plots and forest plots of $\log_2$ fold changes (Gal/control) in PLR or HHR and $\log_2$ ROR (HHR ÷ PLR for interactions model x treatment. In all studies p-value lower than 0.05 or q-value lower than 0.10 were considered significant."

5. Figure 6 appears to reconstruct a model distinct from earlier figures. The motivation, differences, and added value relative to the primary platform are not explained.

We thank the reviewer for this comment and appreciate the opportunity to clarify the rationale for including the decellularized ovary model shown in current Figure 7. Both the plasma-derived 3D model and the decellularized ovary platform can be cultured under defined physoxic or hypoxic conditions and therefore allow investigation of oxygen- and ECM-dependent features of HGSC. The primary distinction between these systems is not oxygen control, but rather availability and experimental scalability. The plasma-based 3D model represents our primary experimental platform due to its reproducibility and accessibility, enabling systematic analyses across multiple conditions and donors. In contrast, decellularized human ovarian tissue preserves native ECM architecture and biochemical complexity but is limited by donor availability, making it less suitable for large-scale or iterative studies. We therefore incorporated the decellularized ovary model as a complementary validation system to confirm that key findings observed in the plasma-derived platform are maintained in a native ovarian ECM context. To improve clarity, we have revised the manuscript text and aligned the terminology of the decellularized models with the primary platform, referring to them as dPLR and dHHR. Additionally, we have included new picrosirius red staining to support the preservation of collagen alignment in the decellularized model. Corresponding changes have been made in the Results section.

Results: Page 23, Lines 558-561: "To evaluate whether key observations from the plasma-derived 3D system are preserved in a native ECM context, we employed a complementary 3D culture platform based on decellularized human ovarian ECM tissue (dECM). Both platforms were cultured under physoxic or hypoxic conditions, enabling assessment of oxygen- and ECM-dependent effects across systems.

Results: Page 23, Lines 568-573: "Histological assessment included confirmation of loss of nuclei by H&E staining and preservation of ECM by Masson's trichrome, collagen I, fibronectin and picrosirius red (PSR) staining in the decellularized matrix compared to native tissue (Fig. 7c). Collagen fibers orientation in PSR staining showed that dECM and native tissue had similar alignment with dominant orientation in

0º and displayed moderate dispersion of the peaks consistent with structurally organized but not rigid collagen network in native ovary (Fig. 7d)."

Results: Page 24, Lines 578-581**: "**Therefore, we refer to them as, "decellularized physoxia-low remodeling of ECM" model (dPLR) and "decelluarized hypoxia-high remodeling of ECM" model (dHHR)."

Results: Page 24, Lines 590-591: "Together, these complementary models enabled evaluation of HGSC tumor–immune interactions in both a scalable plasma-derived matrix and a biologically complex native ovarian ECM."

Discussion: Page 27, Lines 651-653: **"**While both platforms support oxygen-dependent modeling, the plasma-derived system enables broader experimental scalability, whereas decellularized ovarian ECM provides a native validation context constrained by tissue availability."

6.Discovery and "validation" both occur in vitro. There is no confirmation in human tumor tissues that TGF-β/CAF/COL1 axes and the proposed rescue signatures track with immune infiltration in patients. Please include orthogonal validation in clinical material linking CAF/TGF-β/COL1 to immune exclusion.

We thank the reviewer for this important suggestion and agree that orthogonal validation in clinical material strengthens the translational relevance of the study. To address this, we performed immunohistochemical analysis of HGSC patient tumor sections, assessing $CD8^+$ T cells, collagen, and TGF-β. Regions with low $CD8^+$ T-cell infiltration exhibited increased collagen deposition, consistent with immune-excluded tumor architecture. These data are presented in Supplementary Fig. 12, and several sections have been revised accordingly.

Introduction: Page 5-6, Lines 124-128: "Validation studies confirmed low $CD8^+$ T cells presence in areas with high collagen expression in HGSC patients, as well as, significantly impaired immune infiltration associated with hypoxia-induced CAF-driven ECM remodeling in decellularized human ovaries, which was reduced by preincubation with galunisertib."

Figures: Supplementary Fig. 12 has been included

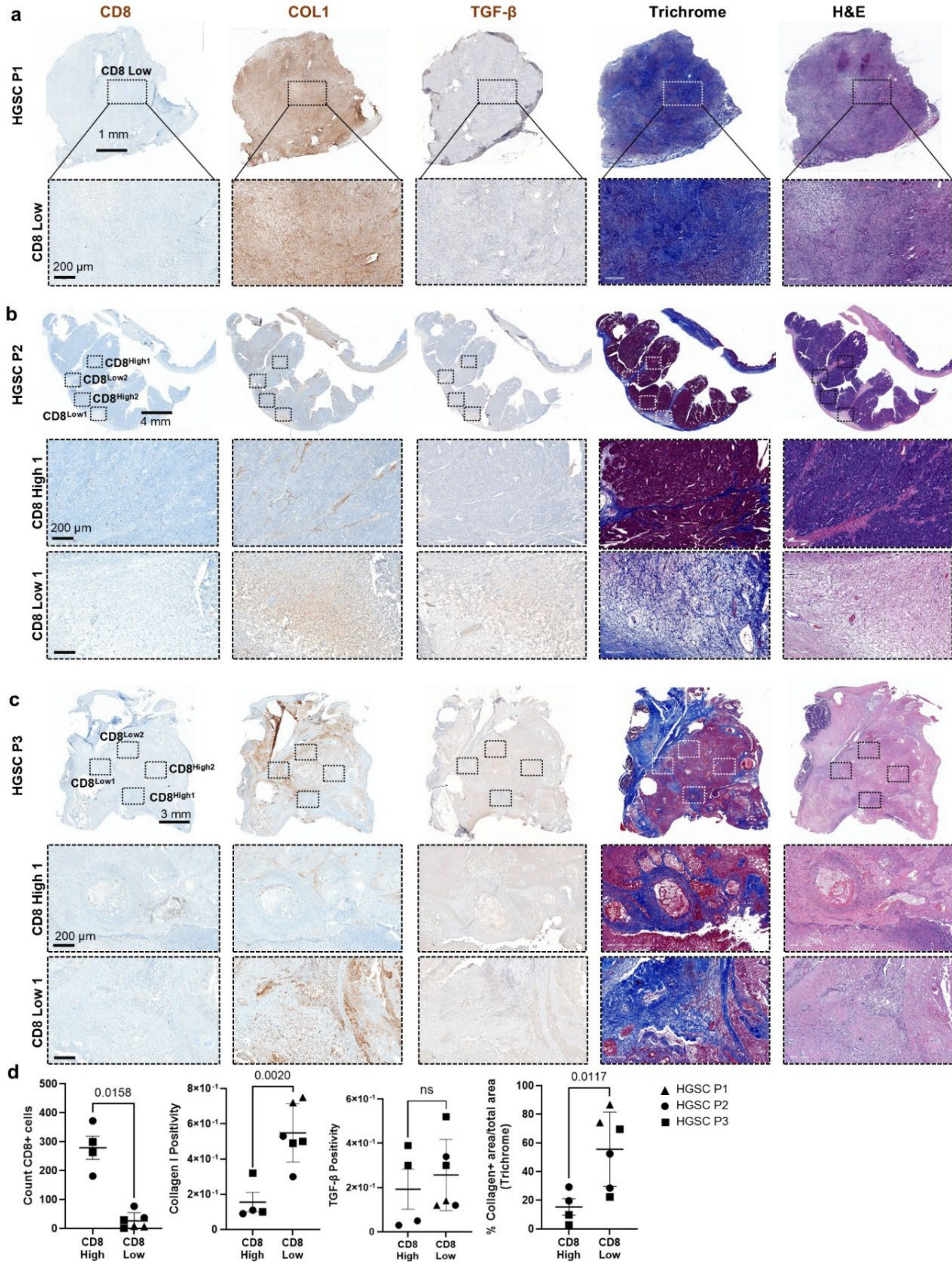

Supplementary Fig. 12. Reduced CD8⁺ T-cell infiltration is associated with increased collagen I deposition in HGSC tumors. a,b,c Representative IHC images of HGSC tumor sections for 3 patients (HGSC P1-3) stained for CD8+ T cells, collagen I, TGF-β, Masson's trichrome and H&E with magnified view of one Region of Interest (ROIs) with low CD8+ and high CD8+ T cell abundance/patient are shown, except for immunologically cold HGSC P1, in which only CD8 low ROI was identified. Scale= 200μm. **d** Quantification of counts (CD8+ T cells), positivity (collagen I and TGF-β), and area of coverage (% blue (collagen)/total area) in $CD8^{Low}$ and $CD8^{High}$ ROIs/patient. n=3, t-test. ns=not significant.

Results: Pages 22-23, Lines 542-554: **"**Once demonstrated that hypoxia-induced CAF-driven TGF-β signaling and collagen I expression is associated with impaired immune infiltration, and that these effects can be disrupted by TGF-β inhibition, we next asked whether these relationships are preserved within the spatial architecture of human HGSC tumors. Immunohistochemistry was performed on tissue sections from three HGSC patients, staining for $CD8^+$ T cells, collagen I, TGF-β, Masson's trichrome, and H&E (Supplementary Fig. 12 a-c). For each patient, two regions of interest (ROIs) with low $CD8^+$ T-cell abundance and two ROIs with high $CD8^+$ T-cell abundance were quantified, except for HGSC patient 1, which exhibited an immunologically deserted tumor phenotype and lacked regions with high $CD8^+$ infiltration. Across samples, regions with low $CD8^+$ T-cell presence were associated with significantly higher collagen deposition compared to immune-infiltrated regions. Although no statistically significant difference in TGF-β expression was observed, an increasing trend in TGF-β levels was detected in collagen-rich, immune-excluded areas (Supplementary Fig. 12d). These observations are consistent with an association between extracellular matrix deposition and immune exclusion in HGSC tumors."

Discussion: Page 28, Lines 695-698: **"**Although limited in sample size, the observed association between collagen-rich regions and reduced $CD8^+$ T-cell infiltration in HGSC patient tissues supports the clinical relevance of the CAF-driven ECM remodeling mechanisms identified *in vitro.*"

Methods: Page 33, Lines 818-819: "Paraffin-embedded HGSC tissues were provided by Sanford Biobank, Sioux Falls, SD."

Methods: Page, 40 Lines 984-985: "The paraffin-embedded HGSC tissues were processed in similar way."

Methods: Page 40, Lines 993-994: "HGSC tumor tissues were stained for CD8+ T cells, collagen I and TGF-β."

Methods: Page 41, Lines 999-1004**:** "Signal intensity and positivity (area of positive staining divided by total tissue area) were measured using the positive pixel count v9 algorithm in at least five randomly picked regions of interest (ROI) per section in cell lines or in two ROIs with high or low CD8 T cells abundance in HGSC patients. The positivity for stromal staining (blue) in Masson's trichrome staining was determined in ImageJ (FIJI) applying first the color deconvolution plugin and same threshold for all ROIs."

Minor comments:

1. The authors describe the result in the multi-culture as having "lower expression of collagen I" in line 197. This phrasing is problematic. I think "expression" is biologically imprecise for a deposited extracellular protein, maybe "**level" or "deposition**" would be more accurate.

The authors would like to thank the reviewer for the suggestion and apologize for the imprecise wording. The expression of "lower expression of collagen I" was changed to "lower deposition of collagen I". The following change has been made in the manuscript:

Results: Page 8 Lines 195-198: "Third, to assess the impact of oxygen tension on ECM remodeling, immunohistochemical analysis revealed significantly higher deposition of collagen I and increased expression of α-SMA, a marker of CAF activation, in KURAMOCHI cells co-cultured with CAFs under HHR conditions compared to PLR (Fig. 1k)."

2. The title of Figure 3 asserts that hypoxia-induced, CAF-driven ECM remodeling “causes” impaired immune infiltration. Given the largely correlative design and limited perturbations that decouple biochemical cues from biomechanical barriers, the data support association rather than causation.

The authors appreciate the reviewer’s suggestion and agree on changing the word “causes” to “is associated with” . The previous Fig. 3 was changed to Fig. 4 during revisions of the manuscript and the title of the new Figure 4 was changed.

The following changes have been made:

Results: Page 16 Line 381-382: **“The HHR model preserves hypoxia-induced CAF-driven ECM remodeling associated with impaired immune infiltration”**

3. The y-axis range is excessively broad in Figure 4d (right panel), the range of which is excessively broad.

The authors would like to thank the reviewer for their comment. The y-axis in the right panel in the previous Figure 4d (current Fig. 5d because of the changes in the manuscript) was set to the same scale as the graph on the left in the CAFs to demonstrate the very low expression of TGF-β in HGSC cells compared to CAFs. Both cells were cultured and detected together in the same experimental tube and therefore we consider that they should have the same scale on y-axis.

The authors apologize to the reviewer, but they consider that no changes should be done in order to better represent the levels of expression of the molecule in both cell types for better comparison.

4. Figure 2d–g suggests lower collagen I under tECMHyp in some contexts, yet stiffness is higher. A mechanistic explanation is missing.

We thank the reviewer for this comment and apologize for the confusion. The apparent discrepancy arises from differences in cellular composition across conditions. Matrix stiffness measurements were performed in co-cultures of HGSC cells and CAFs in the absence of PBMCs, whereas some collagen I measurements initially included multicultures containing PBMCs.

In HGSC–CAF co-cultures, hypoxic conditions consistently resulted in increased collagen I deposition and higher matrix stiffness, demonstrating a direct relationship between CAF-driven collagen production and biomechanical stiffening. In contrast, the addition of PBMCs reduced net collagen accumulation under hypoxia, which we attributed to increased matrix remodeling activity (Supplementary Fig. 2a).

To avoid further confusion, we revised the figure organization and updated Fig. 1e (current FIf. 1k) to display collagen I data from HGSC–CAF co-cultures, which now aligns with the stiffness measurements shown in Fig. 1n (previous Fig. 1h). The Results section has been revised accordingly.

Figures: Figure 1k and Supplementary Figure 2a,b have been modified

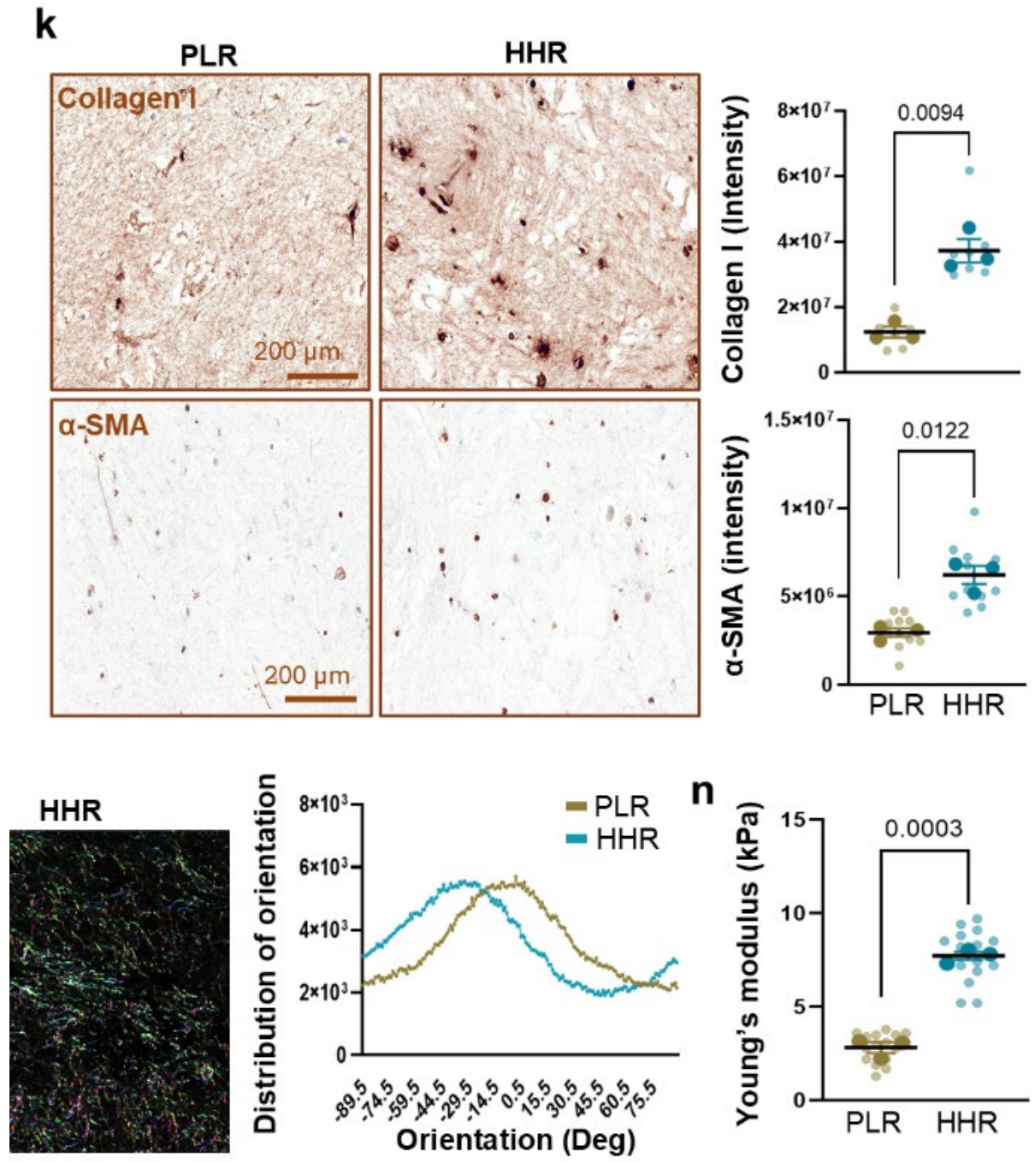

Fig. 1. Transcriptomic concordance and functional divergence across hypoxia and ECM remodeling in patient-derived 3D models. k Representative IHC images for collagen I and α-SMA in co-culture of KURAMOCHI with CAFs and their quantification, scale=200µm. n=3 (3 ROIs). **n** Stiffness quantification by AFM represented by Young's Modulus (kPa) in co-cultures n=3 (5 measurements). k, n Mean±SD, t-test.

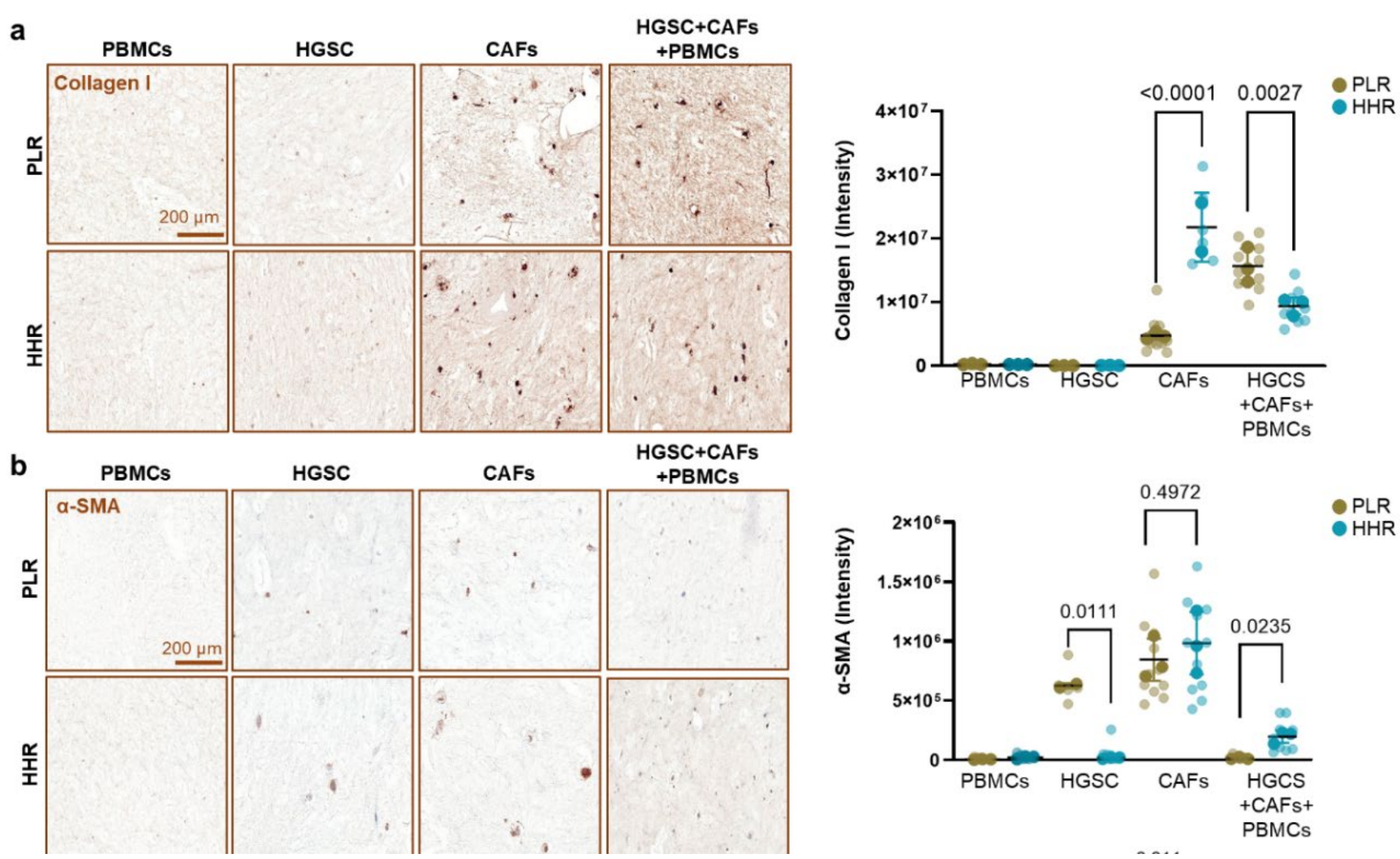

Supplementary Fig. 2. HHR mimics hypoxia-induced CAF-driven ECM remodeling. a-b Representative IHC images and quantification of collagen I and α-SMA intensity in monoculture of PBMCs, HGSC (KURAMOCHI), CAFs or their multi-culture. Mean±SD n=3 (4 ROIs).

Results: Pages 8-9, Lines 195-215: **"**Third, to assess the impact of oxygen tension on ECM remodeling, immunohistochemical analysis revealed significantly higher deposition of collagen I and increased expression of α-SMA, a marker of CAF activation, in KURAMOCHI cells co-cultured with CAFs under HHR conditions compared to PLR (Fig. 1k). CAF monocultures also exhibited increased collagen I deposition under HHR, although at lower levels than observed in co-cultures, while no collagen I or α-SMA expression was detected in KURAMOCHI monocultures. In contrast, when PBMCs were included in multicultures, collagen I deposition was reduced despite sustained CAF activation (Supplementary Fig. 2a,b). Consistent with increased matrix remodeling activity, MMP-9 expression was significantly elevated in hypoxic multicultures containing PBMCs, but not in monocultures or HGSC–CAF co-cultures (Supplementary Fig. 2c,d). Second harmonic generation microscopy confirmed that collagen fiber formation occurred only in the presence of CAFs, independent of oxygen condition. Collagen fibers in co-cultures displayed differences in the orientation with preferred fiber direction in HHR defined by the shift of the peak towards approximately -35° compared to PLR (0°) and slightly more aligned organization in HHR defined by broader distribution of orientation in PLR (Fig. 1l,m). No differences were observed in the distribution of collagen fibers in CAF monocultures between PLR and HHR (Supplementary Fig. 2e,f). Atomic force microscopy measurements performed on HGSC–CAF co-cultures demonstrated significantly higher matrix stiffness under HHR compared to PLR (Fig. 1n), consistent with increased collagen deposition in the absence of immune-mediated matrix remodeling. Together, these results indicate that hypoxia promotes CAF activation and collagen-driven ECM stiffening, while the presence of immune cells introduces additional matrix remodeling dynamics that modulate net collagen accumulation without abrogating hypoxia-induced stiffening."

REVIEWERS' COMMENTS

Reviewer #1 (Remarks to the Author):

The authors have made substantial efforts to clarify points raised in the review of their work, as well as adding considerable amounts of analysis and suggested experiments to clarify the model interpretability. Minor remaining comments to address at this point are:
- image and plot axis labels should, where possible, be increased in size
- fluorescence intensities should be, where possible increased for image viewability

Aside from these, the authors have satisfied the criteria for publication.

We thank the reviewer for their positive assessment of our revisions and for acknowledging the additional analyses and improvements made to enhance model interpretability.

In response to the remaining comments, we have, whenever possible, increased label size and adjust fluorescence intensities to improve viewability.

Reviewer #2 (Remarks to the Author):

The revised manuscript has addressed most of my previous concerns. The added spatial imaging strengthens the evidence that the HHR model recapitulates impaired immune infiltration, and the revised statistical analyses are more appropriate. The additional HGSC tissue validation, although limited in sample size, provides supportive evidence for the association between collagen deposition and immune exclusion. I have no further major concerns. I only suggest that the authors further temper statements implying that galunisertib definitively converts immunologically cold tumors into hot tumors, as the current data primarily support a model-based and hypothesis-generating conclusion.

We thank the reviewer for their thoughtful evaluation of our revised manuscript and for recognizing the improvements in spatial imaging, statistical analyses, and additional HGSC tissue validation.

We appreciate the reviewer's suggestion regarding the interpretation of galunisertib's effects. In response, we have revised the text to temper statements implying a definitive conversion of immunologically "cold" tumors into "hot" tumors via galunisertib. The language has been adjusted to more appropriately reflect that our findings are model-based and hypothesis-generating, while still supporting a potential role for galunisertib in modulating the tumor immune microenvironment.

We believe this revision improves the accuracy and balance of our conclusions and thank the reviewer for this important recommendation.
